# Synthesis of Nonsymmetrical 3,3′-Bicoumarins: Total Synthesis of Arteminorin C, 3,3′-Biisofraxidin, and Biscopoletin

**DOI:** 10.1021/acs.orglett.5c01119

**Published:** 2025-05-20

**Authors:** Mario Castañón-García, Pedro López-Mendoza, Dazaet Galicia-Badillo, Luis D. Miranda

**Affiliations:** † 7180 Instituto de Química, Universidad Nacional Autónoma de México, Circuito Exterior S.N., Ciudad Universitaria, Coyoacán, Ciudad de México 04510, Mexico; ‡ Postdoctoral Associate CONAHCyT, Centro de Investigación de la Facultad de Ciencias Químicas, Benemérita Universidad Autónoma de Puebla (BUAP), 14 Sur Esq. San Claudio, Col. San Manuel, 72570 Puebla, Mexico

## Abstract

A strategy to access nonsymmetrical 3,3′-bicoumarins employing two consecutive Perkin reactions is reported. This approach is based on the initial formation of coumarin acetic acid from a salicylaldehyde derivative through a Perkin reaction. A second Perkin condensation between the previously formed coumarin acetic acid and a new functionalized salicylaldehyde allows the formation of nonsymmetrical 3,3′-bicoumarins. Using this approach, 17 nonsymmetrical and two symmetrical 3,3′-bicoumarins were prepared in yields ranging from 30% to 86%, and natural products arteminorin C, 3,3′-biisofraxidin, and biscopoletin were obtained.

## Graphical abstract

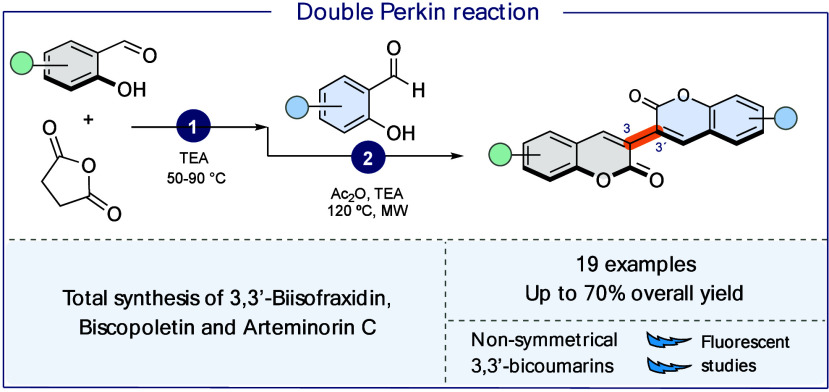

Coumarins are a family of oxygen-based heterocycles that exhibit relevant biological properties^1^ and exciting applications as fluorescent dyes,^2^ organic light-emitting diodes^3^ (OLEDs), optical brighteners,^4^ nonlinear optical chromophores,^5^ and fluorescent markers for physiological measurements.^6^ Interestingly, these organic compounds can form dimeric structures that consist of two identical or different coumarin units covalently connected at different ring positions.^7^ Naturally occurring bicoumarins isolated from different sources possess interesting biological properties.^7^ For example, arteminorin C (**1**), a nonsymmetrical 3,3′-bicoumarin, isolated from the Chinese plant *Artemisia minor*, which is used in traditional medicine to treat fever, rheumatism, dysentery, scabies, and bruising, has shown xanthine oxidase (XOD) inhibition activity (Figure 1).^8^ 3,3′-Biisofraxidin (**2**) isolated from the herb *Sarcandra glabra* induces the *in vitro* and *in vivo* apoptosis of human gastric cancer BGC-823 cells.^9^ Biscopoletin is another 3,3′-bicoumarin isolated from a Chinese plant of the genus *Crossostephium chinense*, and its whole herbs are used for the treatment of diabetes, wind-cold type of common cold, carbuncle, and furuncle.^10^

**Figure 1:**
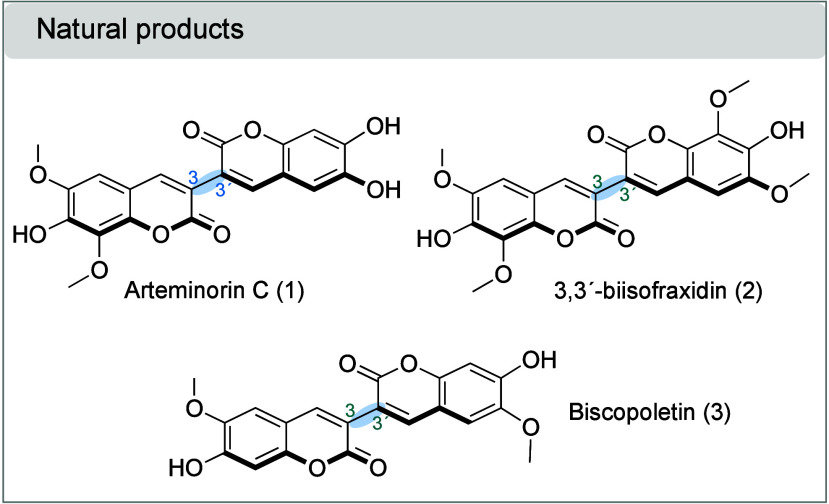
Naturally occurring 3,3′-bicoumarins with biological activity.

Due to their unique biological properties, some laboratories have focused on the synthesis of these compounds,^11^ and the total syntheses for the symmetric 3,3′-bicoumarins biscopoletin^11a^ and 3,3′-biisofraxidin^11b^ have been reported. On the other hand, arteminorin C has not yet been synthesized, which can be attributed to the lack of general approaches to access nonsymmetric 3,3′-bicoumarins. The available methods to construct symmetric 3,3′-bicoumarins, mainly based on transition metal-promoted oxidative dimerization of the monomeric unit,^12^ cannot be applied for the preparation of nonsymmetric 3,3′-bicoumarins.

To the best of our knowledge, only one example for the synthesis of nonsymmetric 3,3′-bicoumarins has been described in the literature. In 2015, Alami and colleagues^13^ reported a Pd-catalyzed C­(sp^2^)–C­(sp^2^) decarboxylative coupling to biheterocycles derived from quinolinones, chromones, and coumarins. By employing 3-coumarin carboxylic acids **4** and 3-bromo-coumarin **5** as coupling partners, they obtained the corresponding nonsymmetric 3,3′-bicoumarins **6**; however, this work reported only two examples (Scheme 1a). Due to their intriguing properties, developing novel and general strategies to access nonsymmetrical 3,3′-bicoumarins in a simple and efficient way is a needed line of investigation.

**Scheme 1:**
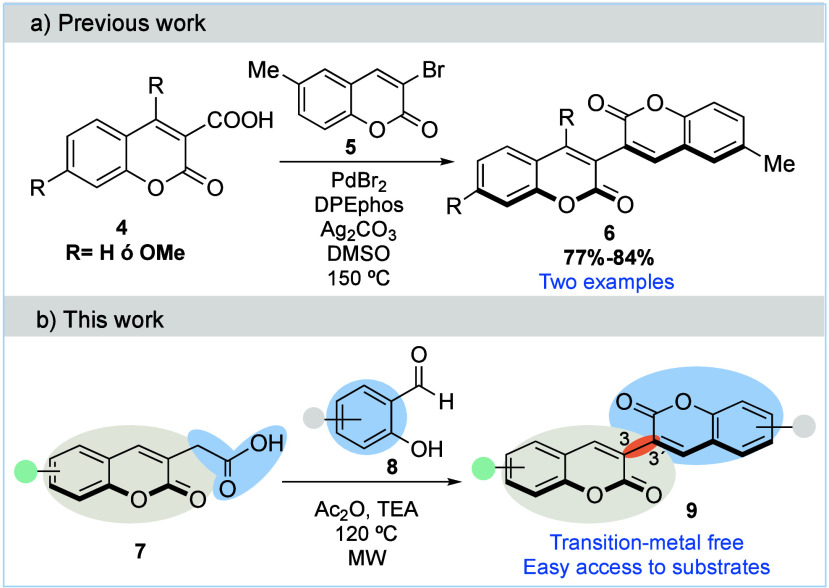
Synthetic Approaches to Nonsymmetrical 3,3′-Bicoumarins

Herein, we report a synthetic approach for the construction of nonsymmetrical 3,3′-bicoumarins **9** through a Perkin reaction between substituted 2-(2-oxo-2*H*-chromium-3-yl)­acetic acids **7**, also prepared by a Perkin condensation, and substituted salicylaldehydes **8** (Scheme 1b) without the use of an external solvent and under metal-free conditions.

We began our study by preparing a series of functionalized 2-(2-oxo-2*H*-chromen-3-yl)­acetic acids **7** through a Perkin reaction following the conditions reported by Zhou and co-workers in 2013.^14^ Then, by employing various substituted salicylaldehydes (1.0 equiv), succinic anhydride (3.2 equiv), and triethylamine (2.6 equiv) under solvent-free conditions and conventional heating (method A), compounds **7a**–**f** were obtained in low to moderate yields (Scheme 2). To improve the yield of coumarin acetic acid **7**, a modification of the reaction conditions was tested. Accordingly, the use of dimethylformamide (DMF) as the solvent and heating under microwave irradiation using the same stoichiometric ratio (method B) resulted in an increased yield in most cases, giving the corresponding 2-(2-oxo-2*H*-chromen-3-yl)­acetic acids **7** in 36–97% yields. Using method B, substrates **7i** and **7j**, the precursors of biscopoletin, 3,3′-biisofraxidin, and arteminorin C, were also prepared in good yields. The lower yield observed for compounds **7d** and **7e** using method B might be due to the formation of a gummy solid that hindered the proper stirring of the reaction mixture and prevented complete consumption of the starting material.

**Scheme 2:**
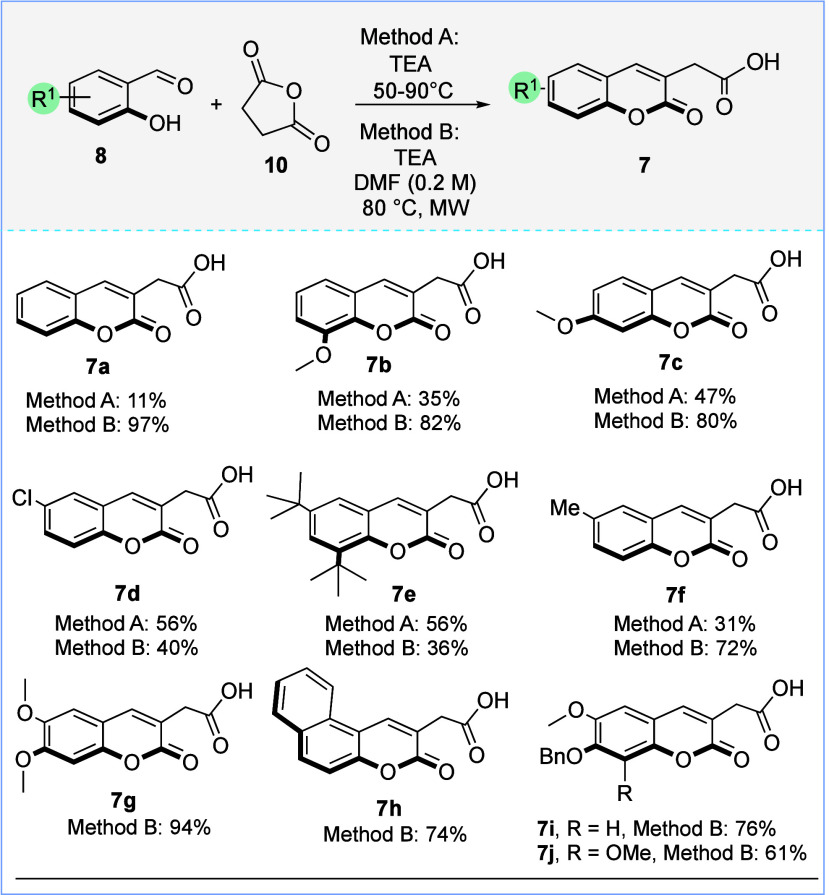
Synthesis of Functionalized 2-(2-Oxo-2*H*-chromen-3-yl)­acetic Acids **7**

With the method for the preparation of 2-(2-oxo-2*H*-chromen-3-yl)­acetic acids **7** established, we then explored the construction of 3,3′-bicoumarins by the reaction between coumarin acetic acids **7** and salicylaldehyde derivatives **8** considering two different routes. In the first approach, we proposed to use a coupling reagent to generate ester intermediate **11**, which would cyclize through an intramolecular Knoevenagel condensation to form 3,3′-bicoumarin **9** (Scheme 3a). An intermediate similar to **11** has been previously suggested by Phakhodee and co-workers in the Ph_3_P/I_2_-mediated synthesis of 3-arylcoumarins from salicylaldehyde derivatives and arylacetic acids.^15^ In a second approach, we proposed that a Perkin reaction using acetic anhydride would generate **12**, the common intermediate in the accepted mechanism for the Perkin reaction, which finally would give the corresponding 3,3′-bicoumarin after cyclization (Scheme 3b).^16^

**Scheme 3:**
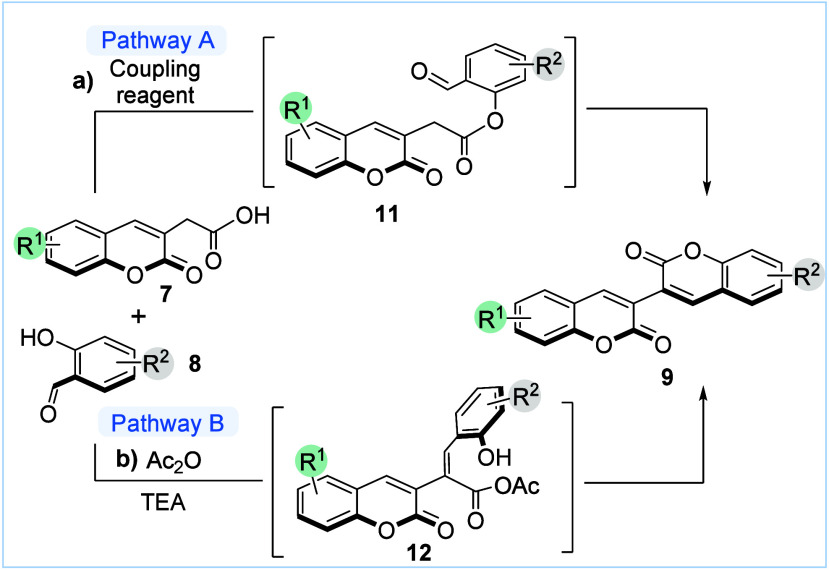
Approaches for the Construction of 3,3′-Bicoumarins

We started the synthesis of 3,3′-bicoumarins using 2-(7-methoxy-2-oxo-2*H*-chromen-3-yl)­acetic acid (**7c**) and 3,5-di-*tert*-butyl-2-hydroxybenzaldehyde (**8a**) as model substrates with different coupling agents (Table 1). Thus, when the Mukaiyama reagent was used in the presence of DIPEA in DCM at 40 °C, 3,3′-bicoumarin **9a** was obtained although in a modest 15% yield (Table 1, entry 1). The structure of **9a** was confirmed by the X-ray crystallographic analysis of a single crystal. The expected compound was obtained with almost the same chemical yield by changing the solvent to 1,2-dichloroethane (1,2-DCE) and increasing the temperature to 80 °C under microwave irradiation (Table 1, entry 2).

**Table 1:**
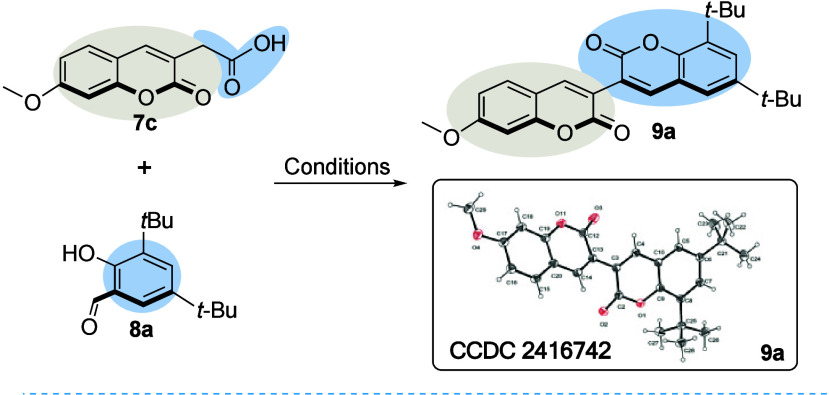
Optimization of the Reaction Conditions^a^

entry	coupling reagent	solvent	base	*T* (°C)	*t* (h)	yield (%)
1	Mukaiyama	DCM	DIPEA	40	16	15
2^b^	Mukaiyama	1,2-DCE	DIPEA	80 (MW)	2	14
3^d^	Mukaiyama	1,2-DCE	DBU	80 (MW)	2	46
4^c^	CDI	MeCN	DIPEA	60	16	trace
5	EDCI	DCM	DMAP	rt	12	18
6^d^	PPh_3_/I_2_	DCM	TEA	rt	20	13
7^d^	PPh_3_/NBS	DCM	TEA	rt	20	0
8	Ac_2_O	–	TEA	80 (MW)	1	61
9	Ac_2_O	–	TEA	80 (MW)	2	51
10	Ac_2_O	–	TEA	100 (MW)	1	48
11^e^	Ac_2_O	–	TEA	120 (MW)	1	62

a Reaction conditions: 1.2:1 **7c**:**8a** molar ratio, solvent. Ellipsoids shown at the 50% probability level.

b 1,2-DCE.

c MeCN.

d At a 1:1.1 molar ratio.

e With 18 equiv of Ac_2_O.

When a stronger base such as DBU was used, **9a** was obtained in 46% yield after the reaction mixture was heated at 80 °C under microwave irradiation (Table 1, entry 3). Subsequently, by changing the coupling reagent to carbonyldiimidazole (CDI) and using DIPEA in acetonitrile (MeCN) at 60 °C, only traces of compound **9a** were obtained (Table 1, entry 4). Afterward, *N*-(3-(dimethylamino)­propyl)-*N*′-ethylcarbodiimide (EDCI) was tested in the presence of DMAP, affording **9a** in 18% yield (Table 1, entry 5). Next, we proceeded to test the reaction conditions reported by Phakhodee,^15^ but we isolated only 13% of **9a** (Table 1, entry 6). On the other hand, when NBS replaced molecular iodine, the reaction did not proceed (Table 1, entry 7). We speculated that the low yield obtained for the construction of the second bicoumarin unit was attributed to the decreased nucleophilic reactivity of the OH group in salicylaldehyde due to the presence of the carbonyl electron-withdrawing group at the *ortho* position, making the esterification reaction inefficient. With this in mind, we then proceeded to evaluate a Perkin reaction for the building of the second coumarin unit to target 3,3′-bicoumarin **9a**. Employing the same model substrates **7c** and **8a**, a Perkin reaction was tested using acetic anhydride and TEA as the base. After heating at 80 °C under microwave irradiation for 1 h, 3,3′-bicoumarin **9a** was obtained in 61% yield (Table 1, entry 8).

The increase in the reaction time to 2 h was not beneficial for the reaction outcome (Table 1, entry 9). On the other hand, an increase in reaction temperature to 120 °C afforded product **9a** in a slightly better 62% yield (Table 1, entry 10). After evaluating both approaches, we determined that the best reaction conditions were as follows: 1.2 equiv of acid **7c**, 1.0 equiv of salicylaldehyde derivative **8a**, 3 equiv of triethylamine, and 18.0 equiv of acetic anhydride at 120 °C under microwave irradiation for 1 h (Table 1, entry 11).

Having established the strategy for the construction of 3,3′-bicoumarins through two consecutive Perkin reactions, we proceeded to evaluate the scope of this approach (Scheme 4). First, we evaluated the effect of the substituent at salicylaldehyde derivative **8** using 2-(7-methoxy-2-oxo-2*H*-chromen-3-yl)­acetic acid **7c**. Under the standard conditions, nonsymmetrical 3,3′-bicoumarins **9b**–**g** were obtained in moderate to good yields. We observed a remarkable electronic effect of the substituent on the yield. For instance, the highly electron-withdrawing nitro group at salicylaldehyde derivative **8** gave the best results, generating product **9e** in 73% yield. In contrast, the presence of an electron-donating group, such as the methyl group, resulted in a moderate 30% yield (**9d**). Next, we explored the use of different coumarin acetic acids **7** in combination with 3,5-di-*tert*-butyl-2-hydroxybenzaldehyde (**8a**). In general, good yields were obtained for the corresponding 3,3′-bicoumarins **9h**–**j**. Subsequently, we explored a modular approach by the combination of different coumarin acetic acids **7** and functionalized salicylaldehydes **8** to obtain a series of structurally diverse nonsymmetrical 3,3′-bicoumarins. Some functional groups such as methoxy, methyl, *tert*-butyl, nitro, and chloro were well tolerated, and products **9l**–**p**, respectively, were obtained in yields ranging from 48% to 86%. Interestingly, when we tried to synthesize compound **9b** again, but with the use of 2-(2-oxo-2*H*-chromen-3-yl)­acetic acid (**7a**) and 2-hydroxy-4-methoxybenzaldehyde (**8c**), product **9b** was not formed. These results clearly demonstrated the effect of the substituents of the salicylaldehyde in the second Perkin reaction. Nevertheless, the modular nature of our approach allows us to interchange the components in both Perkin reactions to obtain the desired product, as was demonstrated with the preparation of **9b** from 2-(7-methoxy-2-oxo-2*H*-chromen-3-yl)­acetic acid (**7c**) and the salicylaldehyde (**8a**).

**Scheme 4:**
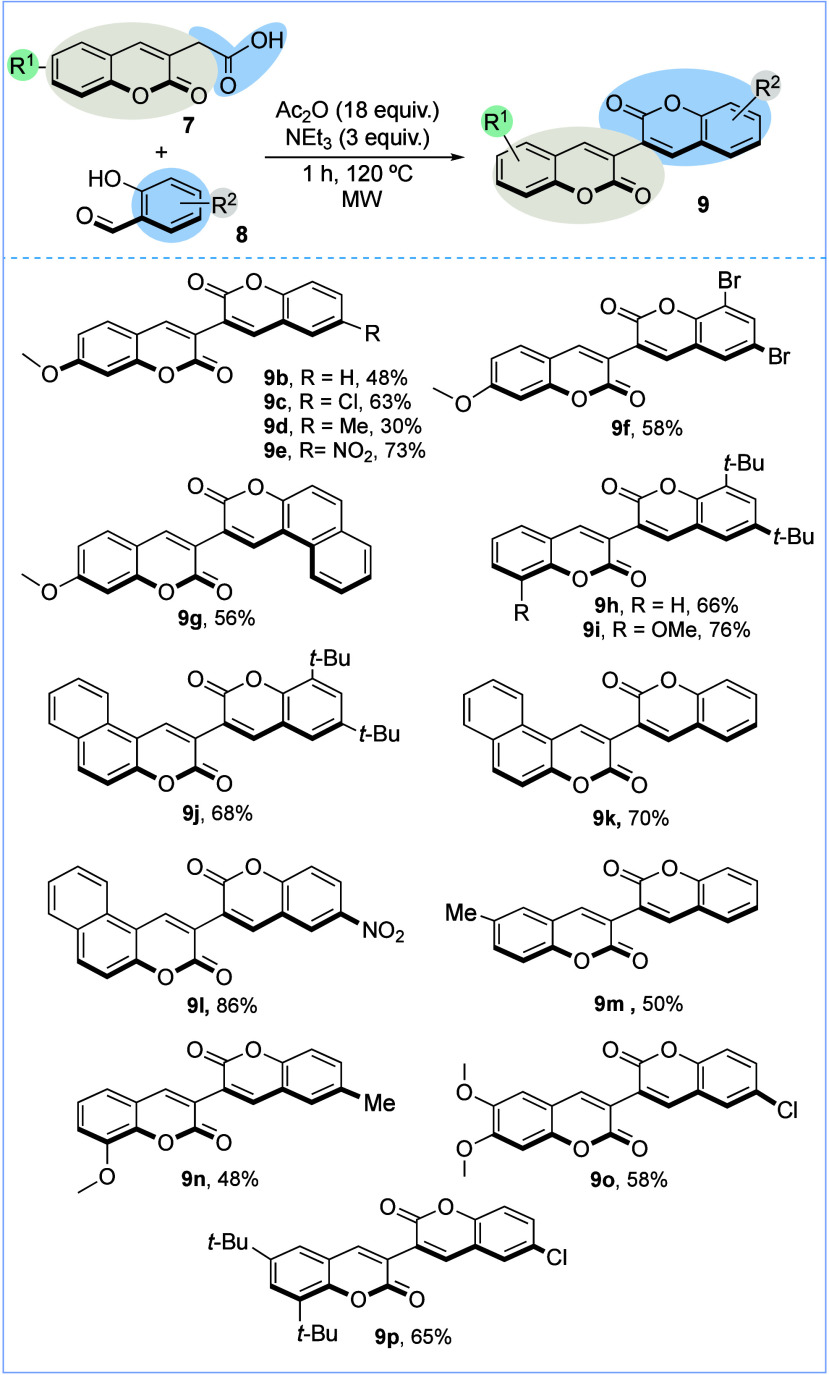
Synthesis of 3,3′-Bicoumarins

We then demonstrated the synthetic utility of the approach with the total synthesis of arteminorin C, 3,3′-biisofraxidin, and biscopoletin (Scheme 5). To this end, 3,3′-bicoumarin **9q** was prepared from **7i** and **8i**. On the other hand, using coumarin acetic acid **7j**, compounds **9r** and **9s** were obtained under the standard conditions using salicylaldehydes **8j** and **8k**, respectively (for the preparation of salicylaldehydes, see the Supporting Information). Finally, catalytic hydrogenolysis of **9q**–**s** with Pd/C and H_2_ gave biscopoletin, 3,3′-biisofraxidin, and arteminorin C, respectively.

**Scheme 5:**
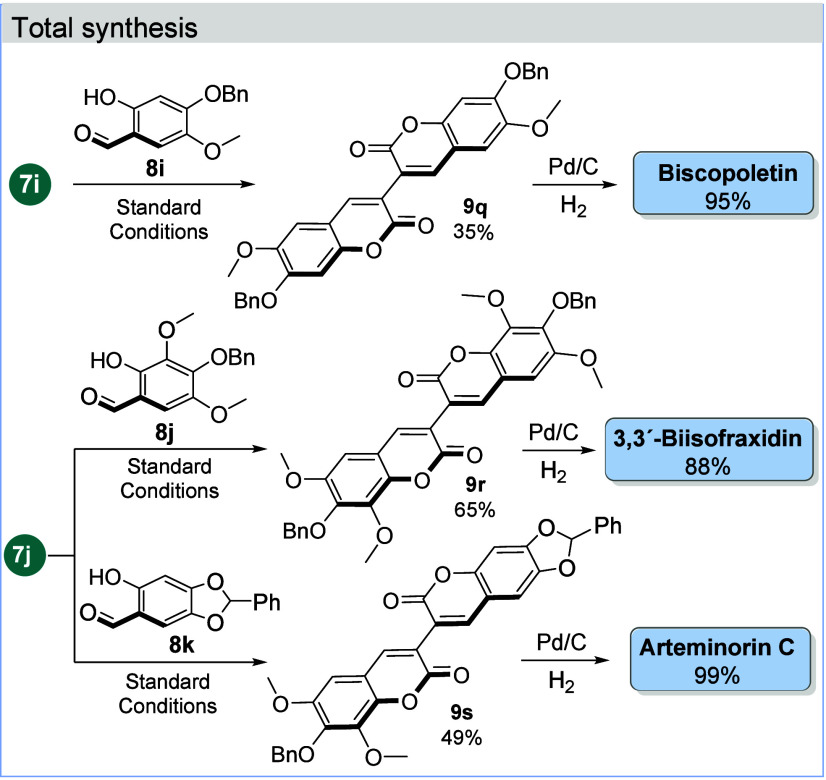
Total Synthesis of Biscopoletin, 3,3′-Biisofraxidin, and Arteminorin C

Lastly, we investigated the steady-state absorption and emission profiles of compounds **9a**–**p** to corroborate their fluorescence behavior. Absorption and emission spectra were recorded in toluene, dioxane, THF, and DMF at a concentration of 1.5 × 10^–5^ M to analyze their photophysical properties in different media. As expected, all compounds exhibited maximum absorption between 361 and 394 nm and maximum emission between 413 and 455 nm in toluene (complete data in the Supporting Information).

Furthermore, moderated solvatofluorochromism behavior was observed for compounds **9n** and **9o** as shown by bathochromic shifts of 47 and 53 nm, respectively, between their emission in toluene and DMF (Figure 2). These results demonstrate that this synthetic protocol can be employed to obtain new fluorescent probes^17^ or as dual-state emitters (DSEgens).^18^

**Figure 2:**
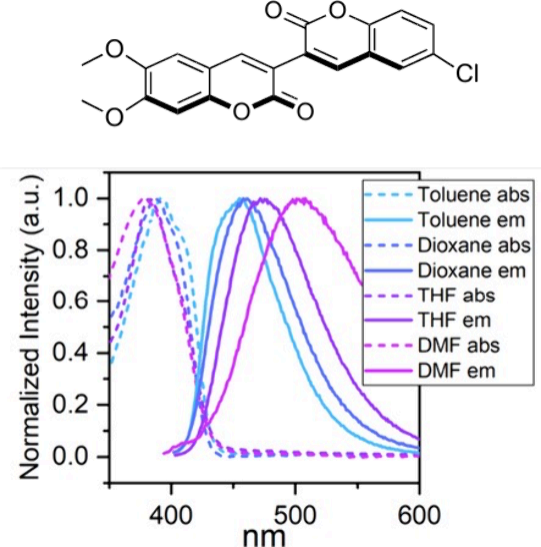
Normalized absorption and emission spectra of **9o** in different solvents. **9o** collected at a concentration of 1.5 × 10^–5^ M. In toluene, λ_abs_ = 366 nm and λ_em_ = 455 nm. In dioxane, λ_abs_ = 362 nm and λ_em_ = 461 nm. In THF, λ_abs_ = 361 nm and λ_em_ = 472 nm. In DMF, λ_abs_ = 347 nm and λ_em_ = 508 nm.

In summary, we have developed a strategy to access nonsymmetrical 3,3′-bicoumarins by iterative Perkin reactions. First, a Perkin condensation between a salicylaldehyde derivative and succinic anhydride affords 2-(2-oxo-2*H*-chromen-3-yl)­acetic acid. A successive Perkin reaction between the coumarin acetic acid derivative and a second substituted salicylaldehyde in the presence of acetic anhydride allows the formation of nonsymmetrical 3,3′-bicoumarins. This novel approach might allow access to naturally occurring and synthetic biologically relevant nonsymmetrical 3,3′-bicoumarins, as demonstrated with the total synthesis of biscopoletin, 3,3′-biisofraxidin, and arteminorin C. In addition, the luminescent properties of the synthesized 3,3′-bicoumarins may permit exploration for further applications. Such investigations are currently underway in our laboratory.

## Supplementary Material



## Data Availability

The data underlying this study are available in the published article and its Supporting Information.

## Notes

Experimental procedures and ^1^H and ^13^C NMR spectra of products (PDF)
