# Supplementary material for: Synthesis of Nonsymmetrical 3,3′-Bicoumarins: Total Synthesis of Arteminorin C, 3,3′-Biisofraxidin, and Biscopoletin
Source: Org Lett. 2025 May 20;27(21):5349–54. doi: 10.1021/acs.orglett.5c01119 (PMC12131221; doi:10.1021/acs.orglett.5c01119)
Supplement: Supplementary file 1 [file ol5c01119_si_001.pdf]

Supporting Information

Synthesis of Non-Symmetrical 3,3'-Bicoumarins: Total Synthesis of Arteminorin C, 3,3'-Biisofraxidin and Biscopoletin

Mario Castañón-García[a], Pedro López-Mendoza[b], Dazaet Galicia-Badillo[a], Luis D. Miranda[a*].

[a]*Instituto de Química, Universidad Nacional Autónoma de México, Circuito Exterior S.N., Ciudad Universitaria, Coyoacán, Ciudad de México, 04510, Mexico.*
[b] *Instituto de Química, Universidad Nacional Autónoma de México, Circuito Exterior S.N., Ciudad Universitaria, Coyoacán, Ciudad de México, 04510, México.*
Actual affiliation: Postdoctoral Associate CONAHCyT, Centro de Investigación de la Facultad de Ciencias Químicas, Benemérita Universidad Autónoma de Puebla (BUAP), 14 Sur Esq. San Claudio, Col. San Manuel 72570, Puebla, México. orcid.org/0000-0002-5958-6013
*Email: lmiranda@unam.mx

Table of Contents

1. General information 1
2. Experimental procedures 1
2.1. Synthesis of 2-(2-oxo-2*H*-chromen-3-yl)acetic acid derivates 1
2.1.1. General procedure 1
2.1.2 Experimental data for 2-(2-oxo-2*H*-chromen-3-yl)acetic acid derivates 2
2.1.2.1. Synthesis of salicylaldehyde 8i: 4
2.1.2.2 Synthesis of salicylaldehyde 8j: 5
2.1.2.3 Synthesis of salicylaldehyde 8k: 6
2.2. Synthesis of non-symmetrical 3,3´-bicoumarins 6
2.2.1. General procedure 6
2.2.2. Experimental data for non-symmetrical 3,3´-bicoumarins 6
2.3. Synthesis of Arteminorin C, 3,3´-biisofraxidin and Biscopoletin 12
2.3.1 Deprotection of benzyl ether. 12
2.3.2 Experimental data for Arteminorin C, 3,3´-biisofraxidin and Biscopoletin 12
2.4 Comparison of the NMR Data of Natural and Synthetic Products. 13
3. NMR spectra 17
4. X-ray Crystallographic data 53
5. Absorption and emission in different solvents of bicoumarins. 55
6. References. 56

1. General information

All salicylaldehydes, chemicals and solvents were purchased from Sigma-Aldrich or Tecsiquim. The reactions were heated by microwave irradiation and no inert systems or anhydrous solvents were required. Reactions progress was monitored by analytical thin layer chromatography (TLC) using silica gel 60 F254 plates purchased from Merck. Monitoring was achieved by shortwave UV light (254 nm). Chromatographic purification of the products was accomplished by flash chromatography on silica gel technical grade (Merck, 230-400 mesh). Melting points were determined on a Fisher-Johns apparatus and are uncorrected. The compounds with high melting point were determined on Differential Scanning Calorimetry (DSC). ^{1}H and ^{13}C NMR spectra were recorded on a Jeol Eclipse-300 MHz, Bruker Avance III 400 MHz and 700 MHz model spectrometers using DMSO-d_6 and $CDCl_3$ as solvents. NMR coupling constants are reported in Hertz (Hz). Chemical shifts (δ) are reported in parts per million (ppm) and the residual solvent peak was used as an internal reference. Multiplicity was indicated as follows: s (singlet), d (doublet), t (triplet), q (quartet), p (quintet), m (multiplet), dd (doublet of doublet), bs (broad singlet). The MS-DART spectra were obtained on a JEOL DART AccuTOF JMS-T100CC and the FAB spectrum was obtained with JEOL JMS-700 MStation. X-ray diffraction studies were performed on a Bruker Smart APEX II CCD diffractometer with graphite-monochromatic Mo Kα irradiation.

2. Experimental procedures

2.1. Synthesis of 2-(2-oxo-2*H*-chromen-3-yl)acetic acid derivates

2.1.1. General procedure

Method A: In a round-bottom flask with a magnetic stir-bar were added salicylaldehyde (15.0 mmol, 1.0 equiv) and succinic anhydride (48.0 mmol, 3.2 equiv). The mixture was stirred at 50 °C for 10 minutes. Then, triethylamine (39.0 mmol, 2.6 equiv) was added dropwise and the reaction was heated at 90 °C for 2 h. After this time, the mixture was acidified with concentrated hydrochloric acid until pH=4. The precipitate was filtered under vacuum and then redissolved in a saturated $NaHCO_3$ solution. Activated carbon was added and the mixture was heated for 15 minutes before hot filtration. The mother liquor was acidified with concentrated hydrochloric acid until pH=3. The precipitate obtained was filtered and washed with cold water to obtain the corresponding 2-(2-oxo-2H-chromen-3-yl)acetic acid derivates.

Method B: In a microwave-type glass vial with a magnetic stir-bar were added salicylaldehyde derivate (1.0 mmol, 1.0 equiv) and succinic anhydride (3.2 mmol, 3.2 equiv). The mixture was dissolved in DMF (5.0 mL, 0.2 M) and TEA were finally added (2.6 mmol, 2.6 equiv). Then, the reaction was heated at 80 °C (42 W) under microwave irradiation and stirred for one hour. After this time, 10 mL of 10% aqueous solution of HCl was added and the mixture was stirred for 10 minutes. The precipitate was filtrated under vacuum and washed with 3 mL of water, followed by 3 mL of chloroform. Subsequently, the filtrate was extracted with chloroform (6 × 20 mL) and the combined

organic phase was washed with a saturated $NaHCO_3$ aqueous solution (4 × 20 mL). Then, the aqueous phase was acidified with concentrated HCl until pH=2. Finally, the aqueous phase was extracted with ethyl acetate (3 × 50 mL) and combined organic phase was dried with anhydrous Na_2SO_4 and filtrated. Finally, the solvent was evaporated under reduced pressure to obtain the corresponding 2-(2-oxo-2H-cromen-3-yl)acetic acid.

2.1.2 Experimental data for 2-(2-oxo-2*H*-chromen-3-yl)acetic acid derivates

2-(2-oxo-2*H*-chromen-3-yl)acetic acid (7a):

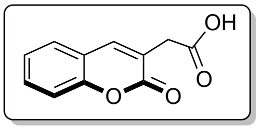

Yellow solid, Method B (199 mg, 97%), m.p. 157-158 °C, (lit. 157-158 °C). **^{1}H NMR** (400 MHz, $CDCl_3$) δ 8.72 (s, 1H), 7.69 (s, 1H), 7.52 – 7.44 (m, 2H), 7.38 – 7.19 (m, 2H), 3.58 (s, 2H). **^{13}C NMR** (100 MHz, $CDCl_3$) δ172.6, 161.6, 153.5, 141.6, 131.3, 127.7, 124.5, 122.8, 119.2, 116.6, 36.0. **HRMS** (ESI) m/z calcd. for $C_{11}H_9O_4$ $[M + H]^+$: 205.0500, found 205.0509. **FT-IR** (ATR): ν (cm^{-1}) 3075, 3066, 2921, 2849, 2737, 2621, 2545, 1719, 1684, 1608, 1580, 1484, 1385, 1332, 1275, 1109, 876, 788, 774, 737, 536.

2-(8-methoxy-2-oxo-2*H*-chromen-3-yl)acetic acid (7b):

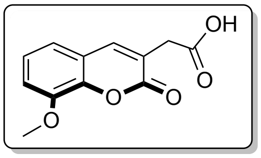

White solid, Method B (193 mg, 82%), m.p. 178-181 °C. **^{1}H NMR** (400 MHz, DMSO-d_6) δ 12.48 (s, 1H), 7.95 (s, 1H), 7.29 (d, *J* = 2.4 Hz, 1H), 7.27 (s, 1H), 7.22 (dd, *J* = 5.7, 3.5 Hz, 1H), 3.91 (s, 3H), 3.50 (s, 2H). **^{13}C NMR** (100 MHz, DMSO-d_6) δ171.3, 160.4, 146.4, 142.2, 141.9, 124.6, 123.3, 119.6, 119.2, 113.7, 56.1, 35.8. **HRMS** (DART) m/z calcd. for $C_{12}H_{11}O_5$ $[M + H]^+$: 235.0606, found 235.0613. **FT-IR** (ATR): ν (cm^{-1}) 3075, 3066, 2921, 2849, 2737, 2621, 2545, 1719, 1684, 1608, 1580, 1484, 1385, 1332, 1275, 1109, 876, 788, 774, 737, 536.

2-(7-methoxy-2-oxo-2*H*-chromen-3-yl)acetic acid (7c):

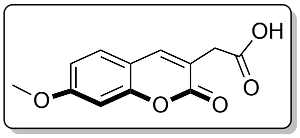

Yellow solid, Method B (187 mg, 80%), m.p. 171-173 °C. **^{1}H NMR** (300 MHz, DMSO-d_6) δ 12.34 (s, 1H), 7.90 (s, 1H), 7.60 (d, *J* = 8.6 Hz, 1H), 7.01 (d, *J* = 2.5 Hz, 1H), 6.95 (dd, *J* = 8.6, 2.4 Hz, 1H), 3.85 (s, 3H), 3.44 (s, 2H). **^{13}C NMR** (75 MHz, DMSO-d_6) δ 171.6, 162.0, 161.0, 154.7, 141.9, 129.1, 119.4, 112.5, 100.6, 55.9, 35.7. **HRMS** (DART) m/z calcd. for $C_{12}H_{11}O_5$ $[M + H]^+$: 235.0606, found 235.0615. **FT-IR** (ATR): ν (cm^{-1}) 2966, 2932, 1718, 1668, 1606, 1414, 1296, 1199, 1176, 1158, 1136, 910, 878, 814, 783, 637, 548.

2-(6-chloro-2-oxo-2*H*-chromen-3-yl)acetic acid (7d):

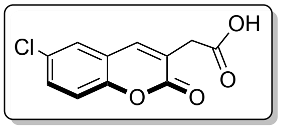

Gray solid, Method B (96 mg, 40%), m.p. 217-220 °C. **^{1}H NMR** (400 MHz, DMSO-d_6) δ 12.53 (s, 1H), 7.93 (s, 1H), 7.82 (d, *J* = 2.5 Hz, 1H), 7.62 (dd, *J* = 8.9, 2.5 Hz, 1H), 7.45 (d, J = 8.9 Hz, 1H), 3.51 (s, 2H). **^{13}C NMR** (100 MHz, DMSO-d_6) δ 171.1, 160.2, 151.5, 140.4, 131.0, 128.4, 127.2, 124.4, 120.4, 118.1, 35.9. **HRMS** (DART) m/z calcd. for $C_{11}H_8ClO_4$ $[M + H]^+$: 239.0111, found 239.0113. **FT-IR**

(ATR): ν (cm^{-1}) 3108, 3054, 2930, 2738, 2629, 1716, 1689, 1602, 1479, 1426, 1337, 1239, 1073, 818, 797.

2-(6,8-di-*tert*-butyl-2-oxo-2*H*-chromen-3-yl)acetic acid (7e):

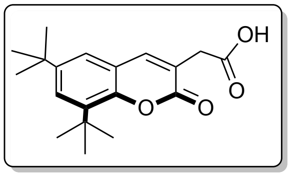

White solid, Method B (114 mg, 36%), m.p. 217-220 °C.**^{1}H NMR** (300 MHz, DMSO-d_6) δ 12.63 (s, 1H), 7.95 (s, 1H), 7.53 (m, 2H), 3.48 (s, 2H), 1.45 (s, 9H), 1.32 (s,9H). **^{13}C NMR** (75 MHz, DMSO-d_6) δ 171.5, 160.5, 149.5, 146.3, 143.0, 135.8, 125.8, 122.9, 122.0, 119.0, 35.7, 34.6, 34.4, 31.1, 29.6. **HRMS** (DART) m/z calcd. for $C_{19}H_{25}O_4$ $[M + H]^+$: 317.1752, found 317.1754. **FT-IR** (ATR): ν (cm^{-1}) 1953, 2806, 2870, 1713, 1583, 1393, 1363, 1233, 1200, 1084, 933, 889, 798, 576.

2-(6-methyl-2-oxo-*2H*-chromen-3-yl)acetic acid (7f):

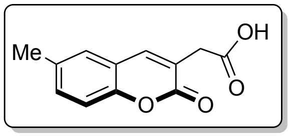

Yellow solid, Method B (158 mg, 72%), m.p: 187-188 °C. **^{1}H NMR** (300 MHz, DMSO-d_6) δ 12.51 (s, 1H), 7.94 (s, 1H), 7.53 – 7.42 (m, 2H), 7.34 (d, *J* = 8.4 Hz, 1H), 3.56 (s, 2H), 2.41 (s, 3H). **^{13}C NMR** (75 MHz, $CDCl_3$) δ 171.4, 160.9, 151.1, 141.7, 134.0, 132.3, 127.7, 123.1, 118.8, 115.9, 35.9, 20.3. **HRMS** (DART) m/z calcd. for $C_{12}H_{11}O_4$ $[M + H]^+$: 219.0657, found 219.0658. **FT-IR** (ATR): ν (cm^{-1}) 2932, 2644, 1702, 1680, 1613, 1574, 1507, 2442, 1392, 1364, 1272, 1252,1194, 1145, 996, 764, 584.

2-(6,7-dimethoxy-2-oxo-2*H*-chromen-3-yl)acetic acid (7g):

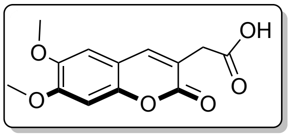

Brown solid, Method B (248 mg, 94%), m.p: 134-135 °C. **^{1}H NMR** (300 MHz, DMSO-d_6) δ 12.32 (s, 1H), 7.84 (s, 1H), 7.19 (s, 1H), 7.07 (s, 1H), 3.85 (s, 3H), 3.80 (s, 3H), 3.45 (s, 2H). **^{13}C NMR** (75 MHz, DMSO-d_6) δ 171.7, 161.3, 152.1, 148.7, 146.0, 141.9, 119.5, 111.4, 108.6, 99.9, 56.2, 55.9, 35.8. **HRMS** (DART) m/z calcd. for $C_{13}H_{13}O_6$ $[M + H]^+$: 265.0712, found 265.0702. **FT-IR** (ATR): ν (cm^{-1}) 3305, 2974, 2938, 2832, 1696, 1632, 1583, 1506, 1432, 1285, 1197, 1145, 1018, 842, 796, 638, 544.

2-(3-oxo-3*H*-benzo[*f*]chromen-2-yl)acetic acid (7h):

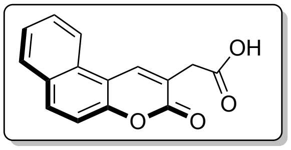

Yellow solid, Method B (189 mg, 74%), m.p: 258-262 °C. **^{1}H NMR** (300 MHz, DMSO-d_6) δ 12.58 (s, 1H), 8.87 (s, 1H), 8.45 (d, *J* = 8.4 Hz, 1H), 8.14 (d, *J* = 9.0 Hz, 1H), 8.04 (d, *J* = 8.0 Hz, 1H), 7.73 (t, *J* = 7.6 Hz, 1H), 7.61 (t, *J* = 7.4 Hz, 1H), 7.55 (d, *J* = 9.0 Hz, 1H), 3.61 (s, 2H). **^{13}C NMR** (75 MHz, $CDCl_3$) δ 171.5, 160.7, 152.4, 138.1, 132.6, 130.0, 128.9, 128.6, 128.3, 126.1, 122.5, 122.1, 116.5, 113.0, 36.2. **HRMS** (DART) m/z calcd for $C_{15}H_{11}O_4$ $[M + H]^+$: 255.0657, found 255.0666. **FT-IR** (ATR): ν (cm^{-1}) 3066, 3022, 2952, 2736, 2545, 1695, 1573, 1517, 1401, 1388, 1232, 1204, 1079, 936, 811, 746, 584.

2.1.2.1. Synthesis of salicylaldehyde 8i:

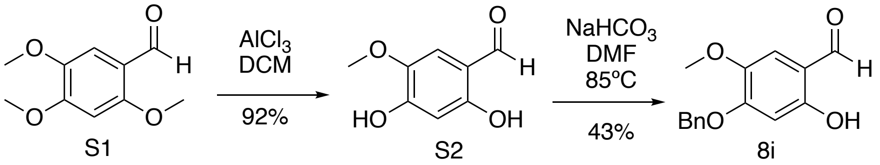

To a suspension of $AlCl_3$ (7.91 g, 59.32 mmol) in anhydrous dichloromethane (35 mL) was added dropwise a solution of 2,4,5-trimethoxybenzaldehyde (2.00 g, 9.88 mmol) in anhydrous dichloromethane (15 mL). Then, the mixture was refluxed for 4 hours. After this time, the reaction was quenched by the addition of ice/water, followed by the addition of 10 mL of concentrated hydrochloric acid. The organic layer was separated, and the aqueous phase was extracted with DCM (3 x 30 mL). The combined organic phase was dried over Na_2SO_4 and filtrated. The solvent was evaporated under reduced pressure and the residue was recrystallized in toluene to give 2,4-dihydroxy-5-methoxybenzaldehyde (**S2**) (1.57 g, 92%).

To a solution of **S2** (1.00 g, 4.16 mmol) and sodium bicarbonate (0.42 g, 4.99 mmol) in dry DMF (28 mL) was added dropwise benzyl bromide (0.87g,4.99 mmol). The resultant solution was then heated at 85°C for 48 hours. After this time, the solution was cooled to room temperature and the reaction was quenched with water and extracted with ethyl acetate (3 x 30 mL). The combined organic phase was dried over Na_2SO_4, filtrated and the solvent was evaporated under reduced pressure. The residue was purified by column chromatography on silica gel (eluent: Hex:AcOEt, 8:2 v/v) to give salicylaldehyde **8i** (0.66 g, 43%) as a white solid. **^{1}H NMR** (300 MHz, $CDCl_3$) δ 11.27 (s, 1H), 9.58 (s, 1H), 7.36 – 7.27 (m, 5H), 6.82 (s, 1H), 6.41 (s, 1H), 5.08 (s, 2H), 3.77 (s, 3H). **^{13}C NMR** (75 MHz, $CDCl_3$) δ 194.1, 159.1, 156.4, 143.2, 135.5, 128.7, 128.5, 128.3, 127.5, 127.3, 126.9, 113.8, 113.0, 101.5, 70.9, 56.5.

2-(7-(benzyloxy)-6-methoxy-2-oxo-2*H*-chromen-3-yl)acetic acid (7i):

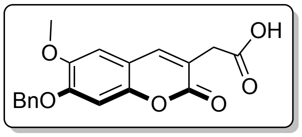

Pale brown solid, Method B (260 mg, 76%), m.p: 190-193°C. **^{1}H NMR** (300 MHz, DMSO-d_6) δ 12.44 (s, 1H), 7.86 (s, 1H), 7.51 – 7.35 (m, 5H), 7.24 (s, 1H), 7.19 (s, 1H), 5.20 (s, 2H), 3.81 (s, 3H), 3.45 (s, 2H). **^{13}C NMR** (75 MHz, DMSO-d_6) δ 171.6, 161.2, 150.9, 148.4, 146.2, 141.9, 136.2, 128.6, 128.2, 128.1, 119.7, 111.6, 108.9, 101.1, 70.3, 56.0, 35.8. **HRMS** (DART) m/z calcd. for $C_{19}H_{17}O_6$ $[M + H]^+$: 341.1025, found 341.1009. **FT-IR** (ATR): ν (cm^{-1}) 3088, 3030, 2939, 1709, 1604, 1575, 1485, 1463, 1424, 1400, 1372, 1354, 1217, 1190, 1155, 1114, 1078, 1030, 987, 916, 831, 741, 698.

2.1.2.2 Synthesis of salicylaldehyde 8j:

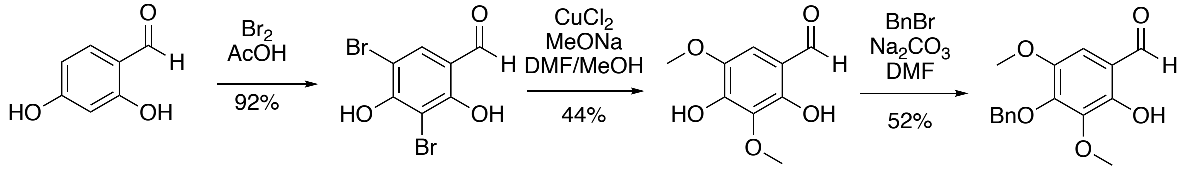

S3 **S4** **S5** **8j**

To a solution of 2,4-dihydroxybenzaldehyde (1g, 7 mmol) in acetic acid (10 mL) was added dropwise bromine (2.31 g, 14 mmol) and the reaction was stirred for 3 h at room temperature. After this time, the resulting mixture was poured into water (15 mL) and the precipitated product was filtered and washed with water. Then, the solid was recrystallized from methanol to give of 3,5-dibromo-2,4-dihydroxybenzaldehyde (**S4**) (1.98 g, 92%),.

To a solution of **S4** (0.7 g, 2.36 mmol) and copper (ll) chloride dihydrate (0.16 g, 0.94 mmol) in dry MeOH/DMF (10 mL/ 1:1 v/v) was added a solution of sodium methoxide (2.56 g,47.31 mmol) in dry MeOH (22 mL). The resultant solution was then heated at 85°C overnight. Then, the reaction was cooled to room temperature and the solvent evaporated under reduced pressure. The crude was redissolved in DCM, hydrochloric acid (10% v/v) was added and extracted with DCM (3 x 30 mL). The combined organic phase was dried with Na_2SO_4, filtrated and the solvent was evaporated under reduced pressure. The residue was purified by column chromatography on silica gel (eluent: Hex:AcOEt, 9:1 v/v) to give salicylaldehyde **S5** (0.204 g, 44% yield) as a brown solid. **^{1}H NMR** (300 MHz, $CDCl_3$) δ 11.30 (s, 1H), 9.68 (s, 1H), 6.73 (s, 1H), 6.58 (s, 1H), 3.98 (s, 3H), 3.89 (s, 3H). **^{13}C NMR** (75 MHz, $CDCl_3$) δ 194.7, 151.7, 147.1, 141.3, 134.7, 112.9, 109.0, 61.0, 56.7.

To a solution **S5** (0.200 g, 1.029 mmol) and sodium bicarbonate (0.10 g, 1.23 mmol) in dry DMF (7 mL) was added benzyl bromide (0.18 g, 1.02 mmol). The resultant solution was then heated at 85°C for 48 hours. After this time, the reaction was cooled to room temperature, quenched with water and extracted with DMC (3 x 30 mL). The combined organic phase was dried over Na_2SO_4, filtrated and the solvent was evaporated under reduced pressure. The residue was purified by column chromatography on silica gel (eluent: Hex:AcOEt, 9.5/0.5 v/v) to give salicylaldehyde **8j** (0.15 g, 52%) as a yellow oil. **^{1}H NMR** (300 MHz, $CDCl_3$) δ 10.95 (s, 1H), 9.77 (s, 1H), 7.51 – 7.45 (m, 2H), 7.40 – 7.28 (m, 3H), 6.78 (s, 1H), 5.23 (s, 2H), 3.89 (s, 2H), 3.84 (s, 3H). **^{13}C NMR** (75 MHz, $CDCl_3$) δ 195.1, 151.7, 149.4, 147.0, 141.8, 137.1, 128.5, 128.3, 115.8, 109.5, 75.5, 61.2, 56.6.

2-(7-(benzyloxy)-6,8-dimethoxy-2-oxo-*2H*-chromen-3-yl)acetic acid (7j):

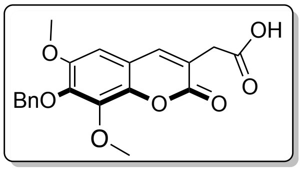

Yellow oil, Method B (227 mg, 61%), **^{1}H NMR** (300 MHz, $CDCl_3$) δ 10.14 (s, 1H), 7.60 (s, 1H), 7.48 – 7.47 (m, 1H), 7.46 – 7.45 (m, 1H), 7.41 – 7.29 (m, 3H), 6.67 (s, 1H), 5.15 (s, 2H), 3.96 (s, 3H), 3.86 (s, 3H), 3.61 (s, 2H). **^{13}C NMR** (75 MHz, $CDCl_3$) δ 175.3, 161.3, 150.7, 144.6, 142.5, 142.2, 141.5, 137.1, 128.5, 128.5, 128.4, 128.3, 128.3, 120.5, 114.9, 103.8, 75.8, 61.9, 56.4, 35.9. **HRMS** (FAB) m/z calcd. for $C_{20}H_{19}O_7$ $[M + H]^+$: 371.1131, found 371.1122. **FT-IR** (ATR): ν (cm^{-1}) 3014, 2934, 1737, 1701, 1613, 1574, 1506, 1462, 1423, 1392, 1274, 1250, 1152, 1081, 1021, 983, 912, 851, 771, 732, 696, 585.

2.1.2.3 Synthesis of salicylaldehyde 8k:

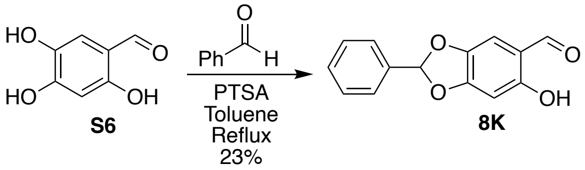

To a solution of 2,4,5-trihydroxybenzaldehyde (0.2g, 1.3 mmol) in toluene (10 mL) was added *p*-Toluenesulfonic acid. The mixture was refluxed with a Dean-Stark system for 24 hours. Then, the reaction was cooled to room temperature and the solvent evaporated under reduced pressure. The crude was redissolved in DCM and extracted with DCM (3 x 30 mL). The combined organic phase was dried with Na_2SO_4, filtrated and the solvent was evaporated under reduced pressure. The residue was purified by column chromatography on silica gel (eluent: Hex:AcOEt, 9:1 v/v) to give salicylaldehyde **8k** (0.071 g, 23% yield) as a white solid, m.p. 105-108 **^{1}H NMR** (300 MHz, $CDCl_3$) δ 11.81 (s, 1H), 9.64 (d, *J* = 0.6 Hz, 1H), 7.59 – 7.50 (m, 2H), 7.49 – 7.43 (m, 3H), 7.03 (s, 1H), 6.90 (s, 1H), 6.50 (s, 1H). **^{13}C NMR** (75 MHz, $CDCl_3$) δ 193.9, 161.7, 155.3, 141.7, 135.2, 130.9, 129.0, 126.4, 113.8, 111.8, 109.5, 98.4.

2.2. Synthesis of non-symmetrical 3,3´-bicoumarins

2.2.1. General procedure

In a microwave-type glass vial with a magnetic stir-bar were added sequentially salicylaldehyde derivative **8** (0.25 mmol, 1.0 equiv), 2-(2-oxo-2H-chromen-3-yl)acetic acid derivative **7** (0.30 mmol, 1.2 equiv), triethylamine (0.75 mmol, 3.0 equiv) and acetic anhydride (4.50 mmol, 18 equiv). Then, the mixture was heated at 120 °C under microwave irradiation for one hour. After this time, the reaction crude was purified by column chromatography on silica gel, or the precipitate was filtration under vacuum.

2.2.2. Experimental data for non-symmetrical 3,3´-bicoumarins

6,8-di-*tert*-butyl-7´-methoxy-2*H*,2'*H*-(3,3´-bichromen)-2,2´-dione (9a):

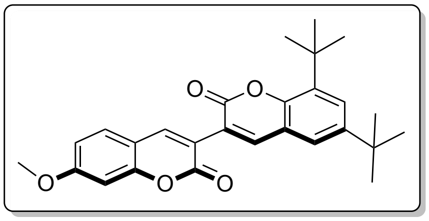

Yellow solid (67 mg, 62%), m.p. 194-195 °C. **^{1}H NMR** (300 MHz, $CDCl_3$) δ 8.59 (s, 1H), 8.57 (s, 1H), 7.58 (d, *J* = 2.2 Hz, 1H), 7.48 (d, *J* = 8.5 Hz, 1H), 7.41 (d, *J* = 2.3 Hz, 1H), 6.93 – 6.82 (m, 2H), 3.89 (s, 3H), 1.54 (s, 9H), 1.36 (s, 9H). **^{13}C NMR** (75 MHz, $CDCl_3$) δ 163.4, 160.7, 160.2, 155.3, 150.1, 147.0, 144.5, 143.7, 137.0, 129.9, 127.5, 123.5, 119.2, 119.2, 116.8, 113.2, 113.0, 100.4, 55.9, 35.2, 34.8, 31.5, 30.0. **HRMS** (DART) m/z calcd. for $C_{27}H_{29}O_5$ $[M + H]^+$: 433.2015, found 433.2015. **FT-IR** (ATR): ν (cm^{-1}) 3001, 2955, 2904, 2869, 1706, 1621, 1603, 1502, 1361, 1237, 1124, 1029, 921, 829,767, 622, 477. The residue was purified by column chromatography on silica gel (eluent: Hex:AcOEt, 9:1 v/v).

Crystals of **9a** were grown from a chloroform solution by slow evaporation at room temperature.

7-methoxy-2*H*,2'*H*-(3,3́-bichromen)-2,2́-dione (9b):

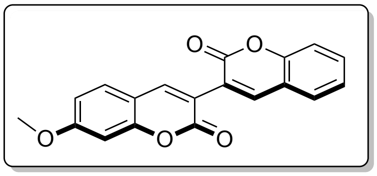

Yellow solid (38 mg, 48%), m.p. 253 °C. **¹H NMR** (300 MHz, $CDCl_3$) δ 8.57 (s, 1H), 8.55 (s, 1H), 7.59 (dd, *J* = 8.0, 1.5 Hz, 1H), 7.56 – 7.47 (m, 2H), 7.38 – 7.33 (m, 1H), 7.30 (dd, *J* = 7.5, 1.1 Hz, 1H), 6.89 (dd, *J* = 8.6, 2.4 Hz, 1H), 6.84 (d, *J* = 2.4 Hz, 1H), 3.90 (s, 3H). **^{13}C NMR** (75 MHz, $CDCl_3$) δ 163.6, 160.6, 160.4, 155.4, 153.3, 144.0, 143.1, 132.2, 130.0, 128.8, 124.8, 120.5, 119.4, 116.6, 116.5, 113.3, 112.9, 100.5, 56.0. **HRMS** (DART) m/z calcd. for $C_{19}H_{13}O_5$ $[M+H]^+$: 321.0763, found 321.0764. **FT-IR** (ATR): ν (cm^{-1}) 3065, 3039, 2925, 2843, 1711, 1610, 1556, 1503, 1454, 1284, 1264, 1239, 1152, 1132, 1028, 921, 756. The residue was purified by column chromatography on silica gel (eluent: Hex:AcOEt, 9:1 v/v, 8:2 v/v).

6-chloro-7'-methoxy-2*H*,2'*H*-[3,3'-bichromene]-2,2'-dione (9c):

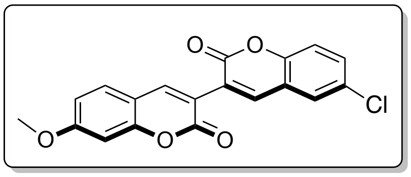

Yellow pale solid (56 mg, 63%), m.p. 299°C (Determined by DSC). **¹H NMR** (300 MHz, $CDCl_3$) δ 8.58 (s, 1H), 8.53 (s, 1H), 7.59 (d, *J* = 2.4 Hz, 1H), 7.55 – 7.46 (m, 2H), 7.31 (d, *J* = 8.9 Hz, 1H), 6.94 – 6.83 (m, 2H), 3.91 (s, 3H). **^{13}C CPMAS ssNMR** (125 MHz) δ 163.9, 162.4, 160.5, 155.7, 151.2, 142.6, 133.7, 125.9, 120.9, 111.3, 100.3, 56.5. **HRMS** (DART) m/z calcd. for $C_{19}H_{12}ClO_5$ $[M+H]^+$: 355.0373, found 355.0384. **FT-IR** (ATR): ν (cm^{-1}) 3082, 3055, 2963, 1718, 1616, 1560, 1505, 1480, 1449, 1357, 1274, 1255, 1241, 1127, 1080, 1024, 925, 827, 765, 627, 614, 484. The precipitate was isolated by vacuum filtration.

7́-methoxy-6-methyl-2*H*,2'*H*-(3,3́-bichromen)-2,2́-dione (9d):

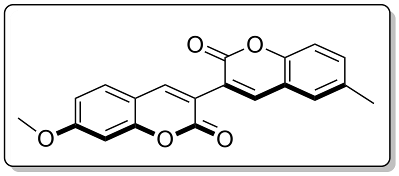

White solid (25 mg, 30%), m.p. 253-254 °C. **¹H NMR** (300 MHz, $CDCl_3$) δ 8.55 (s, 1H), 8.52 (s, 1H), 7.50 (d, *J* = 8.9 Hz, 1H), 7.36 (d, *J* = 9.7 Hz, 2H), 7.23 (s, 1H), 6.97 – 6.73 (m, 2H), 3.90 (s, 3H), 2.42 (s, 3H). **^{13}C NMR** (75 MHz, $CDCl_3$) δ 163.6, 160.6, 155.4, 151.5, 143.9, 143.2, 134.5, 133.5, 133.3, 130.0, 128.6, 120.4, 119.1, 116.8, 116.2, 113.3, 113.0, 100.5, 56.0, 20.9. HRMS (ESI) m/z calcd. for $C_{20}H_{15}O_5$ $[M+H]^+$: 335.0919, found 335.0908. **FT-IR** (ATR): ν (cm^{-1}) 3103, 3047, 3009, 2922, 2845, 1711, 1617, 1555, 1436, 1335, 1292, 1260, 1244, 1143, 1124, 1022, 922, 820, 763, 575, 540, 454. The residue was purified by column chromatography on silica gel (eluent: Hex:AcOEt, 9:1 v/v, 85:15 v/v).

7'-methoxy-6-nitro-2*H*,2'*H*-[3,3'-bichromene]-2,2'-dione (9e):

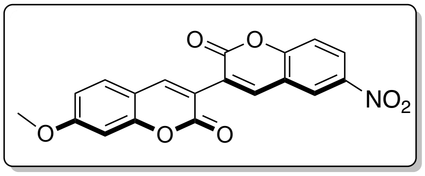

Yellow solid (67 mg, 73%), m.p. 281-285°C (Determined by DSC). **¹H NMR** (300 MHz, $CDCl_3$) δ 8.71 (s, 1H), 8.61 (s, 1H), 8.54 (d, *J* = 2.6 Hz, 1H), 8.41 (dd, *J* = 9.1, 2.6 Hz, 1H), 7.53 (d, *J* = 8.6 Hz, 1H), 7.49 (d, *J* = 9.1 Hz, 1H), 6.93 (dd, *J* = 8.6, 2.4 Hz, 1H), 6.87 (d, *J* = 2.3 Hz, 1H), 3.92 (s, 3H). **^{13}C CPMAS**

ssNMR (125 MHz) δ 162.8, 159.2, 155.7, 143.3, 133.8, 124.6, 111.4, 96.4, 55.5. **HRMS** (DART) m/z calcd for $C_{19}H_{12}NO_7$ $[M + H]^+$: 366.0613, found 366.0597. **FT-IR** (ATR): ν (cm^{-1}) 3107, 3056, 2922, 2850, 1733, 1715, 1611, 1528, 1505, 1346, 1276, 1253, 1226, 1136, 1123, 1024, 978, 948, 930, 921, 835, 763, 623. The precipitate was isolated by vacuum filtration.

6,8-dibromo-7'-methoxy-2*H*,2'*H*-[3,3'-bichromene]-2,2'-dione (9f):

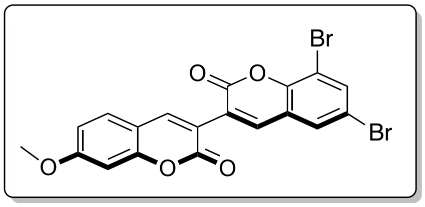

Yellow pale solid (69 mg, 58%), m.p. 308°C (Determined by DSC). **^{1}H NMR** (300 MHz, $CDCl_3$) δ 8.62 (s, 1H), 8.54 (s, 1H), 7.89 (d, *J* = 2.1 Hz, 1H), 7.69 (d, *J* = 2.2 Hz, 1H), 7.53 (d, *J* = 8.7 Hz, 1H), 6.95 – 6.89 (m, 1H), 6.85 (d, *J* = 2.4 Hz, 1H), 3.91 (s, 3H). **^{13}C CPMAS ssNMR** (125 MHz) δ 163.6, 159.7, 154.7, 143.0, 127.3, 120.8, 114.9, 111.0, 103.2, 56.1. **HRMS** (DART) m/z calcd. for $C_{19}H_{11}Br_2O_5$ $[M + H]^+$: 476.8973, found 476.8981. **FT-IR** (ATR): ν (cm^{-1}) 3073, 3002, 2982, 2950, 2841, 1709, 1614, 1547, 1509, 1436, 1334, 1298, 1279,1246, 1224, 1143, 1023, 858, 760, 634, 547. The precipitate was isolated by vacuum filtration.

2-(7-methoxy-2-oxo-2H-chromen-3-yl)-3H-benzo[f]chromen-3-one (9g):

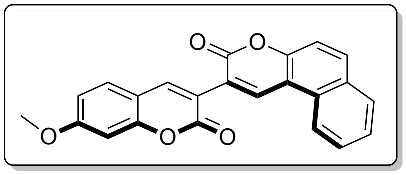

Yellow solid (52 mg, 56%), m.p. 262 °C. **^{1}H NMR** (300 MHz, $CDCl_3$) δ 9.50 (s, 1H), 8.72 (s, 1H), 8.39 (d, *J* = 8.6 Hz, 1H), 8.02 (d, *J* = 9.0 Hz, 1H), 7.93 (d, *J* = 8.0 Hz, 1H), 7.72 (t, *J* = 7.8 Hz, 1H), 7.64 – 7.43 (m, 3H), 6.90 (d, *J* = 12.7 Hz, 2H), 3.92 (s, 3H). **^{13}C CPMAS ssNMR** (125 MHz) δ 163.5, 162.7, 159.5, 154.3, 151.4, 140.6, 130.8, 127.6, 121.6, 116.5, 110.9, 101.6, 54.9. **HRMS** (DART) m/z calcd. for $C_{23}H_{15}O_5$ $[M + H]^+$: 371.0919, found 371.0913. **FT-IR** (ATR): ν (cm^{-1}) 3098, 3048, 3009, 2883, 2958, 2845, 1728, 1699, 1614, 1573, 1507, 1440, 1289, 1268, 1145, 1120, 1021, 915, 842, 811, 738, 637, 578. The precipitate was isolated by vacuum filtration.

6,8-di-*tert*-butyl-2*H*,2'*H*-(3,3′-bichromen)-2,2′-dione (9h):

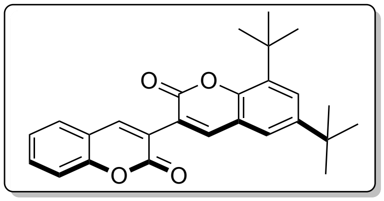

Yellow solid (66 mg, 66%), m.p. 213-213 °C. ^{1}H NMR (400 MHz, $CDCl_3$) δ 8.64 (s, 1H), 8.60 (s, 1H), 7.63 –7.51 (m, 3H), 7.43 (d, *J* = 2.3 Hz, 1H), 7.36 (d, *J* = 8.3 Hz, 1H), 7.31 (td, *J* = 7.5 Hz, 1H), 1.54 (s, 9H), 1.37 (s, 9H). **^{13}C NMR** (100 MHz, $CDCl_3$) δ 160.2, 159.9, 153.3, 150.1, 147.0, 145.1, 143.4, 136.9, 132.2, 128.8, 127.7, 124.7, 123.6, 120.3, 119.2, 119.0, 118.8, 116.4, 35.1, 34.7, 31.4, 29.9. **HRMS** (DART) m/z calcd. for $C_{26}H_{27}O_4$ $[M + H]^+$: 403.1909, found 403.1891. **FT-IR** (ATR): ν (cm^{-1}) 2997, 2964, 2950, 2902, 2868, 1746, 1707, 1580, 1490, 1454, 1360, 1164, 768. The residue was purified by column chromatography on silica gel (eluent: Hex:AcOEt, 9:1 v/v).

6,8-di-*tert*-butyl-8'-methoxy-2*H*,2'*H*-(3,3'-bichromen)-2,2'-dione (9i):

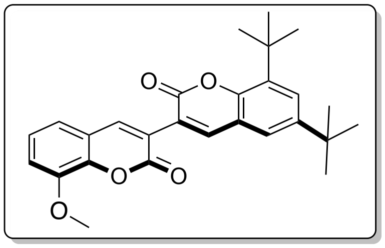

White solid (82 mg, 76%). m.p. 229-231 °C. **^{1}H NMR** (400 MHz, $CDCl_3$) δ 8.57 (s, 1H), 8.56 (s, 1H), 7.52 (d, *J* = 2.3 Hz, 1H), 7.36 (d, *J* = 2.3 Hz, 1H), 7.19 – 7.14 (m, 1H), 7.09 (dd, *J* = 7.9, 1.4 Hz, 1H), 7.03 (dd, *J* = 8.1, 1.4 Hz, 1H), 3.92 (s, 3H), 1.46 (s, 9H), 1.29 (s, 9H). **^{13}C NMR** (100 MHz, $CDCl_3$) δ 160.0, 159.7, 150.1, 147.0, 146.9, 145.2, 143.6, 142.9, 137.0, 127.7, 124.5, 123.6, 120.5, 120.2, 119.9, 119.0, 118.7, 113.8, 56.3, 35.1, 34.7, 31.4, 29.9. **HRMS** (DART) m/z calcd. for $C_{27}H_{29}O_5$ $[M + H]^+$: 433.2015, found 433.2025. **FT-IR** (ATR): ν (cm^{-1}) 3095, 3051, 3002, 2955, 2923, 2868, 1705, 1610, 1586, 1572, 1480, 1459, 1442, 1360, 1345, 1277, 1242, 1144, 1097, 768, 539. The residue was purified by column chromatography on silica gel (eluent: Hex:AcOEt, 95:5 v/v).

2-(6,8-di-*tert*-butyl-2-oxo-2*H*-chromen-3-yl)-3*H*-benzo[*f*]chromen-3-one (9j):

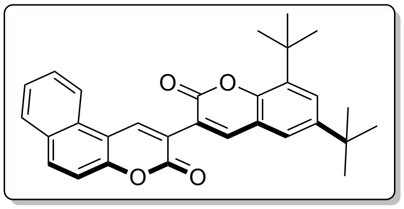

Yellow solid (77 mg, 68%), m.p. 267-268°C **^{1}H NMR** (300 MHz, $CDCl_3$) δ 9.58 (s, 1H), 8.79 (s, 1H), 8.37 (d, *J* = 8.4 Hz, 1H), 8.01 (d, *J* = 9.0 Hz, 1H), 7.91 (d, *J* = 8.1 Hz, 1H), 7.69 (t, *J* = 7.6 Hz, 1H), 7.63 (d, *J* = 2.3 Hz, 1H), 7.57 (t, *J* = 7.5 Hz, 1H), 7.50 – 7.46 (m, 2H), 1.57 (s, 9H), 1.38 (s, 9H). **^{13}C NMR** (75 MHz, $CDCl_3$) δ 160.4, 160.3, 153.1, 150.2, 147.2, 145.1, 139.3, 137.0, 133.8, 130.5, 129.5, 129.1, 128.6, 127.8, 126.3, 123.8, 122.1, 119.2, 119.1, 119.0, 116.6, 113.7, 35.2, 34.9, 31.5, 30.0. **HRMS** (DART) m/z calcd. for $C_{30}H_{29}O_4$ $[M + H]^+$: 453.2065, found 453.2060. **FT-IR** (ATR): ν (cm^{-1}) 2995, 2960, 2902, 2867, 1713, 1699, 1571, 1392, 1361, 1324, 1206, 1155, 1000, 925, 819, 778, 763, 750, 685, 586, 537.The precipitate was isolated by vacuum filtration.

2-(2-oxo-2*H*-chromen-3-yl)-3*H*-benzo[*f*]chromen-3-one (9k):

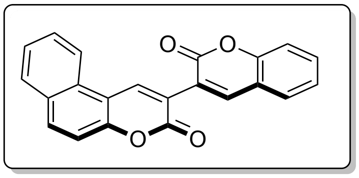

Brown solid (60 mg, 70%), m.p. 262°C. **^{1}H NMR** (300 MHz, $CDCl_3$) δ 9.51 (s, 1H), 8.76 (s, 1H), 8.38 (d, *J* = 8.4 Hz, 1H), 8.04 (d, *J* = 9.0 Hz, 1H), 7.93 (d, *J* = 8.1 Hz, 1H), 7.73 (ddd, *J* = 8.4, 7.0, 1.4 Hz, 1H), 7.67 – 7.54 (m, 3H), 7.50 (d, *J* = 8.7 Hz, 1H), 7.40 (d, *J* = 8.3 Hz, 1H), 7.35 (t, *J* = 7.5 Hz, 1H). **^{13}C NMR** (75 MHz, $CDCl_3$) δ 160.4, 160.3, 153.4, 153.3, 143.6, 139.6, 134.0, 132.4, 130.5, 129.5, 129.1, 129.0, 128.7, 126.4, 124.9, 122.0, 120.4, 119.3, 119.0, 116.6, 116.5, 113.5. Yield: 70%. **HRMS** (DART) m/z calcd. for $C_{22}H_{13}O_4$ $[M + H]^+$: 341.0813, found 341.0818. **FT-IR** (ATR): ν (cm^{-1}) 3062, 1713, 1607, 1564, 1340, 1214, 1126, 957, 912, 809, 744, 573. The precipitate was isolated by vacuum filtration.

2-(6-nitro-2-oxo-2*H*-chromen-3-yl)-3*H*-benzo[*f*]chromen-3-one (9l):

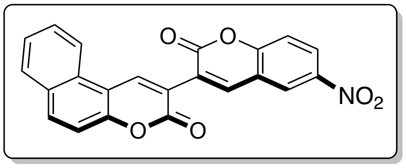

Yellow solid (83 mg, 86%), m.p. 318-323°C (Determined by DSC). **^{1}H NMR** (700 MHz, DMSO-*d*$_6$) δ 9.30 (s, 1H), 8.84 (d, *J* = 2.7 Hz, 1H), 8.68 (s, 1H), 8.53 (d, *J* = 8.4 Hz, 1H), 8.50 (d, *J* = 2.7 Hz, 1H), 8.49 (d, *J* = 2.7 Hz, 1H), 8.30 (d, *J* = 9.0 Hz, 1H), 8.12 (d, *J* = 8.0 Hz, 1H), 7.81 (t, *J* = 8.2 Hz, 1H), 7.75 (d, *J* = 9.1 Hz, 1H),

7.69 – 7.66 (m, 2H). **^{13}C NMR** (175 MHz, DMSO-*d*$_6$) δ 158.6, 158.0, 156.7, 153.3, 143.9, 142.3, 140.0, 134.2, 130.0, 129.1, 128.8, 128.7, 127.0, 126.4, 124.7, 124.0, 122.1, 120.5, 119.1, 117.8, 116.6, 112.7. **HRMS** (DART) m/z calcd. for $C_{22}H_{12}NO_6$ $[M + H]^+$: 386.0664, found 386.0670. **FT-IR** (ATR): ν (cm^{-1}) 3107, 3057, 1737, 1711, 1608, 1572, 1529, 1349, 1216, 1139, 1122, 922, 814, 776, 743, 655, 578. The precipitate was isolated by vacuum filtration.

6-metil-2*H*,2'*H*-(3,3´-bichromen)-2,2´-dione (9m):

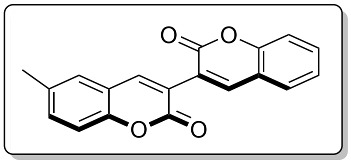

White solid (38 mg, 50%), m.p. 257 °C. **^{1}H NMR** (300 MHz, $CDCl_3$) δ 8.60 (s, 1H), 8.54 (s, 1H), 7.61 (dd, *J* = 7.5, 1.6 Hz, 1H), 7.59–7.54 (m, 1H), 7.41–7.35 (m, 3H), 7.34 – 7.27 (m, 2H), 2.43 (s, 3H). **^{13}C NMR** (75 MHz, $CDCl_3$) δ 160.5, 160.3, 153.5, 151.7, 143.9, 143.8, 134.6, 133.6, 132.5, 129.0, 128.7, 124.9, 120.4, 120.1, 119.3, 119.0, 116.5, 116.3, 21.0. **HRMS** (DART) m/z calcd. for $C_{19}H_{13}O_4$ $[M + H]^+$: 305.0813, found 305.0806. **FT-IR** (ATR): ν (cm^{-1}) 2955, 2917, 2849, 1720, 1617, 1604, 1565, 1455, 1342, 1251, 1122, 927, 821, 750, 623, 590, 511. The residue was purified by column chromatography on silica gel (eluent: Hex-AcOEt, 8:2 v/v)

8'-methoxy-6-methyl-2*H*,2'*H*-[3,3'-bichromene]-2,2'-dione (9n):

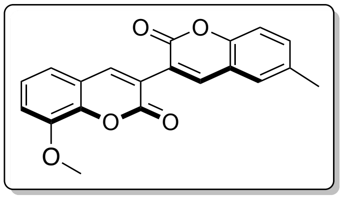

White solid (40 mg, 48%), m.p. 269-271 °C. **^{1}H NMR** (300 MHz, $CDCl_3$) δ 8.60 (s, 1H), 8.59 (s, 1H), 7.49 (d, *J* = 7.9 Hz, 1H), 7.24 (d, *J* = 7.8 Hz, 1H), 7.20 – 7.07 (m, 4H), 4.00 (s, 3H), 2.48 (s, 3H). **^{13}C NMR** (75 MHz, $CDCl_3$) δ 160.5, 159.8, 153.6, 147.0, 144.0, 144.0, 143.7, 143.1, 128.7, 126.1, 124.7, 120.6, 120.3, 120.0, 118.9, 117.0, 116.7, 114.0, 56.4, 22.1. **HRMS** (DART) m/z calcd. for $C_{20}H_{15}O_5$ $[M + H]^+$: 335.0919, found 335.0916. **FT-IR** (ATR): ν (cm^{-1}) 3029, 2988, 2950, 2841, 1713, 1618, 1606, 1581, 1477, 1341, 1278, 1262, 1170, 1143, 1104, 923, 813, 776, 761, 726, 578, 520. The residue was purified by column chromatography on silica gel (eluent: Hex-AcOEt, 85:15 v/v).

6'-chloro-6,7-dimethoxy-2*H*,2'*H*-[3,3'-bichromene]-2,2'-dione (9o)

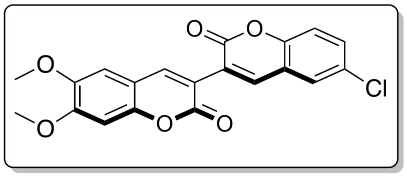

Yellow solid (56 mg, 58%), m.p. 280-284°C (Determined by DSC). **^{1}H NMR** (300 MHz, DMSO-*d*$_6$) δ 8.41 (s, 1H), 8.38 (s, 1H), 7.97 (d, *J* = 2.6 Hz, 1H), 7.70 (dd, *J* = 8.9, 2.5 Hz, 1H), 7.52 (d, *J* = 8.8 Hz, 1H), 7.37 (s, 1H), 7.18 (s, 1H), 3.90 (s, 3H), 3.83 (s, 3H). Yield: 58%. **^{13}C CPMAS ssNMR** (125 MHz) δ 161.1, 153.6, 144.4, 133.1, 129.8, 120.9, 110.2, 102.4, 55.2. **HRMS** (DART) m/z calcd. for $C_{20}H_{14}ClO_6$ $[M + H]^+$: 385.0478, found 385.0467. **FT-IR** (ATR): ν (cm^{-1}) 3065, 2962, 2835, 1708, 1619, 1607, 1562, 1508, 1472, 1448, 1395, 1284, 1271, 1257, 1196, 1148, 1079, 1008, 953, 927, 854, 826, 765, 650, 580, 484, 466. The precipitate was isolated by vacuum filtration.

6,8-di-*tert*-butyl-6'-chloro-2*H*,2'*H*-[3,3'-bichromene]-2,2'-dione (9p):

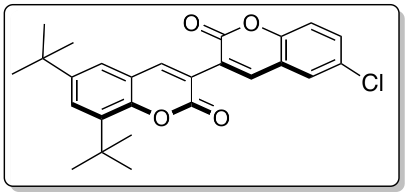

White solid (71 mg, 65%), m.p 186-187°C. **^{1}H NMR** (300 MHz, $CDCl_3$) δ 8.61 (s, 1H), 8.59 (s, 1H), 7.62 (d, *J* = 2.3 Hz, 1H), 7.58 (d, *J* = 2.4 Hz, 1H), 7.51 (dd, *J* = 8.8, 2.4 Hz, 1H), 7.42 (d, *J* = 2.2 Hz, 1H), 7.32 (d, *J* = 8.8 Hz, 1H), 1.54 (s, 9H), 1.37 (s, 9H). **^{13}C NMR** (75 MHz, $CDCl_3$) δ 159.9, 159.7, 151.7, 150.3, 147.2, 145.7, 142.1, 137.1, 132.1, 130.1, 128.1, 128.0, 123.7, 121.5, 120.4, 118.9, 118.4, 118.0, 35.2, 34.8, 31.5, 31.4, 30.0. Yield: 65%. **HRMS** (DART) m/z calcd. for $C_{26}H_{25}ClO_4$ $[M + H]^+$: 437.1439, found 437.1458. **FT-IR** (ATR): ν (cm^{-1}) 2957, 2926, 2869, 1713, 1578, 1565, 1479, 1468, 1362, 1241, 1201, 1189, 1132, 1117, 1076, 997, 920, 898, 819, 767, 703, 649, 550. The precipitate was isolated by vacuum filtration.

7,7'-bis(benzyloxy)-6,6'-dimethoxy-2*H*,2'*H*-[3,3'-bichromene]-2,2'-dione (9q):

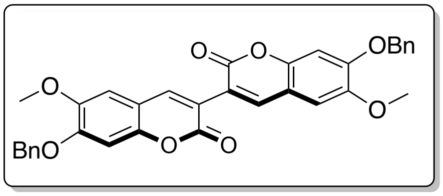

Yellow solid (49 mg, 35%), m.p 254-257°C. **^{1}H NMR** (300 MHz, $CDCl_3$) δ 8.58 (s, 2H), 7.51 – 7.31 (m, 10H), 6.97 (s, 2H), 6.85 (s, 2H), 5.24 (s, 4H), 3.93 (s, 6H). **^{13}C NMR** (75 MHz, $CDCl_3$) δ 160.9, 152.4, 149.1, 147.2, 143.2, 135.7, 129.0, 128.5, 127.4, 117.1, 112.3, 108.9, 101.3, 71.3, 56.5. **HRMS** (DART) m/z calcd. for $C_{34}H_{27}O_8$ $[M + H]^+$: 563.1705, found 563.1685. **FT-IR** (ATR): ν (cm^{-1}) 3082, 3011, 2942, 2918, 2838, 1693, 1612, 1560, 1578, 1500, 1459,1391, 1378, 1223, 1268, 1239, 1201, 1158, 1105, 1024, 993, 839, 756, 737, 692, 602, 589, 468. The precipitate was isolated by vacuum filtration.

7**,7'-bis(benzyloxy)-6,6',8,8'-tetramethoxy-*2H,2'H*-[3,3'-bichromene]-2,2'-dione (9r):**

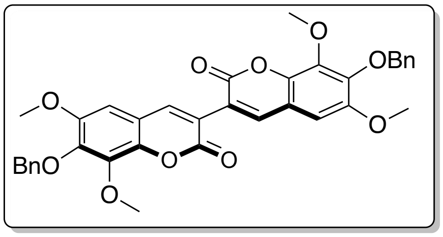

Yellow solid (101 mg, 65%), m.p 202-204°C. **^{1}H NMR** (300 MHz, $CDCl_3$) δ 8.57 (s, 2H), 7.50 – 7.47 (m, 4H), 7.38 – 7.33 (m, 6H), 6.78 (s, 2H), 5.20 (s, 4H), 4.01 (s, 6H), 3.89 (s, 6H). **^{13}C NMR** (75 MHz, $CDCl_3$) δ 160.0, 150.9, 145.3, 143.7, 142.3, 141.3, 137.0, 128.6, 128.4, 118.6, 115.1, 104.6, 75.8, 62.0, 56.5. **HRMS** (DART) m/z calcd. for $C_{36}H_{30}O_{10}$ $[M + H]^+$: 623.6311 found 623.6309 **FT-IR** (ATR): ν (cm^{-1}) 3046, 2921, 2851, 1710, 1605, 1560, 1482, 1457, 1419, 1371, 1289, 1256, 1192, 1107, 1045, 996, 728,570. The residue was purified by column chromatography on silica gel (eluent: Hex-AcOEt, 85:15 v/v).

7-(7-(benzyloxy)-6,8-dimethoxy-2-oxo-*2H*-chromen-3-yl)-2-phenyl-6H-[1,3]dioxolo[4,5-g]chromen-6-one (9s):

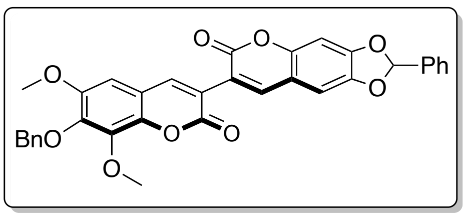

Yellow solid (71 mg, 49%), m.p 172-174°C. **^{1}H NMR** (300 MHz, $CDCl_3$) δ 8.58 (s, 1H), 8.56 (s, 1H), 7.61 – 7.52 (m, 3H), 7.51 – 7.46 (m, 5H), 7.39 – 7.33 (m, 3H), 7.11 (s, 1H), 6.99 (s, 1H), 6.88 (s, 1H), 6.78 (s, 1H), 5.19 (s, 2H), 4.00 (s, 3H), 3.89 (s, 3H). **^{13}C NMR** (75 MHz, $CDCl_3$) δ 160.7, 160.1, 152.0, 150.8, 150.8, 145.5, 145.1, 143.8, 143.3, 142.3,

141.3, 137.1, 135.1, 130.9, 129.0, 128.8, 128.6, 128.5, 128.4, 126.5, 126.4, 118.7, 116.8, 115.1, 113.3, 112.1, 105.8, 104.5, 97.9, 77.4, 75.8, 62.0, 56.4. **HRMS** (DART) m/z calcd. for $C_{34}H_{25}O_9$ $[M + H]^+$: 577.1498, found 577.1495. **FT-IR** (ATR): ν (cm^{-1}) 2919, 2849, 1707, 1568, 1484, 1462, 1370, 1259, 1191, 1159, 1114, 1014, 909, 693. The residue was purified by column chromatography on silica gel (eluent: Hex-AcOEt, 85:15 v/v).

2.3. Synthesis of Arteminorin C, 3,3´-biisofraxidin and Biscopoletin

2.3.1 Deprotection of benzyl ether.

In a glass vial with a magnetic stir-bar bicoumarine derivative (1.0 equiv) was added and dissolved in MeOH (0.01 M). Then, palladium on carbon (20 wt. %) Was added and the reaction was put into a reactor at 120 psi of hydrogen at 25 °C for 12 hours. After this time, the reaction crude was purified by column chromatography on silica gel or the precipitate was isolated of vacuum filtration.

2.3.2 Experimental data for Arteminorin C, 3,3´-biisofraxidin and Biscopoletin

Arteminorin C (1):

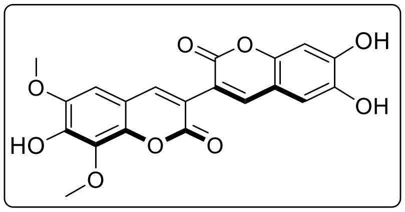

Starter compound (0.061 mmol). Yellow amorphous solid (24 mg, 99%), m.p 150°C decomposition (Determined by DSC). **^{1}H NMR** (700 MHz, DMSO-d_6) δ 8.21 (s, 1H), 8.17 (s, 1H), 7.08 (s, 1H), 7.03 (s, 1H), 6.80 (s, 1H), 3.86 (s, 3H), 3.83 (s, 3H). **^{13}C NMR** (175 MHz, DMSO-d_6) δ 159.9, 159.5, 151.1, 148.3, 146.0, 144.4, 143.4, 143.4, 143.2, 142.6, 134.6, 118.1, 117.4, 112.6, 110.9, 110.4, 104.7, 102.5, 60.9, 56.3. Yield: 99% **HRMS** (DART) m/z calcd. for $C_{20}H_{15}O_9$ $[M + H]^+$: 399.0716, found 399.0724. **FT-IR** (ATR): ν (cm^{-1}) 3261, 2920, 2851, 1676, 1607, 1575, 1503, 1454, 1400, 1287, 1232, 1195, 1154, 1107, 1032, 927, 851, 766, 586. The precipitate was isolated by vacuum filtration and washed with hot methanol and ethyl acetate.

3,3´-biisofraxidin (2):

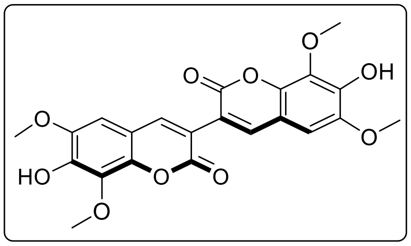

Starter compound (0.014 mmol). Yellow solid (5.6 mg, 88%). **^{1}H NMR** (700 MHz, DMSO-d_6) δ 10.04 (s, 2H), 8.30 (s, 2H), 7.12 (s, 2H), 3.87 (s, 6H), 3.85 (s, 6H). **^{13}C NMR** (175 MHz, DMSO-d_6) δ 159.3, 145.9, 144.4, 143.4, 142.4, 134.5, 117.3, 110.2, 104.7, 60.9, 56.2. **HRMS** (DART) m/z calcd. for $C_{22}H_{19}O_{10}$ $[M + H]^+$: 443.0978, found 443.0975. **FT-IR** (ATR): ν (cm^{-1}) 2955, 2852, 1707, 1607, 1574, 1501, 1458, 1430, 1392, 1298, 1234, 1166, 1104, 1044, 1024, 992, 765, 623. The residue was purified by column chromatography on silica gel (eluent: DCM-MeOH, 99:1 v/v, 94:6 v/v).

Biscopoletin (3):

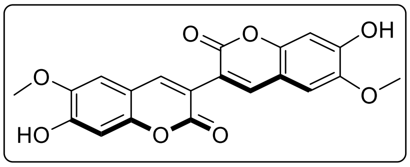

Starter compound (0.020 mmol). Yellow solid (7.1 mg, 95%), m.p 346-352°C (Determined by DSC). **^{1}H NMR** (700 MHz, DMSO-d_6) δ 10.45 (s, 2H), 8.30 (s, 2H), 7.30 (s, 2H), 6.84 (s, 2H), 3.84 (s, 6H). **^{13}C NMR** (175 MHz, DMSO-d_6) δ 159.8, 151.7, 149.1, 145.6, 143.0, 117.1, 110.6, 109.8, 102.5, 56.1. **HRMS** (DART) m/z calcd. for $C_{20}H_{15}O_8$ $[M + H]^+$: 383.0766, found 383.0770. **FT-IR** (ATR): ν (cm^{-1}) 3462, 2921, 1710, 1627, 1608, 1571, 1505, 1400, 1345, 1303, 1270, 1207, 1159, 1119, 1002, 944, 926, 864, 764, 665, 593. The residue was purified by column chromatography on silica gel (eluent: DCM-MeOH, 99:1 v/v, 94:6 v/v).

2.4 Comparison of the NMR Data of Natural and Synthetic Products.

2.4.1 Comparison of the NMR Data of Natural and Synthetic Arteminorin C (1).

Table S1. Comparison of the ^{1}H NMR Data of Natural and Synthetic Arteminorin C (1).

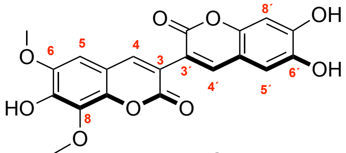

Arteminorin C **(1)**

Position	δ$_H$ in ppm (mult, *J* in Hz)		
	Natural Product[1]	Synthetic product	Δ$_{(S-N)}$
2			
3			
4	8.24 *s*	8.21 *s*	-0.03
5	7.10 *s*	7.08 *s*	-0.02
6			
7			
8			
9			
10			
1´			
2´			
3´			
4´	8.21 *s*	8.17 *s*	-0.04
5´	7.04 *s*	7.03 *s*	-0.01
6´			
7´			
8´	6.80 *s*	6.80 *s*	0

9´			
10´			
6-OCH_3	3.85 *s*	3.83 *s*	-0.02
8-OCH_3	3.87 *s*	3.86 *s*	-0-01
6´-OH			
7´-OH			

Table S2. Comparison of the ^{13}C NMR Data of Natural and Synthetic Arteminorin C (1).

**Position**	**δ_C in ppm**		
	**Natural Product**	**Synthetic product**	**$\Delta_{(S\text{-}N)}$**
2	159.7	159.5	-0.2
3	118.4	118.1	-0.3
4	143.7	143.4	-0.3
5	105.1	104.7	-0.4
6	146.4	146.0	-0.4
7	144.8	144.4	-0.4
8	135.0	134.6	0.6
9	142.9	143.6	0.7
10	110.7	110.4	-0.3
2´	160.2	159.9	-0.3
3´	117.6	117.4	-0.2
4´	143.5	143.2	-0.3
5´	112.9	112.6	-0.3
6´	143.7	143.4	-0.3
7´	151.5	151.1	-0.4
8´	102.8	102.5	-0.3
9´	148.6	148.3	-0.3
10´	111.3	110.9	-0.4
6-OCH_3	56.7	56.3	-0.4
8-OCH_3	61.3	60.9	-0.4
6´-OH			
7´-OH			

2.4.2 Comparison of the NMR Data of Natural and Synthetic 3,3´-biisofraxidin (2).

Table S3. Comparison of the ^{1}H NMR Data of Natural and Synthetic 3,3´-biisofraxidin (2).

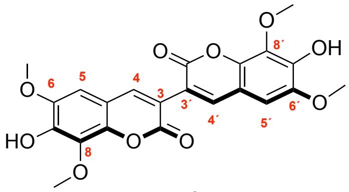

3,3´-biisofraxidin **(2)**

Position	δ_H in ppm (mult, *J* in Hz)		
	Natural Product[2]	Synthetic product	$\Delta_{(S-N)}$
2,2´			
3,3´			
4,4´	8.30 *s*	8.30 *s*	0
5,5´	7.12 *s*	7.12 *s*	0
6,6´			
7,7´			
8,8´			
9,9´			
10,10´			
6,6´-OCH_3	3.87 *s*	3.87 *s*	0
8,8´-OCH_3	3.85 *s*	3.85 *s*	0
7,7´-OH	10.06 *s*	10.04 *s*	-0.02

Table S4. Comparison of the ^{13}C NMR Data of Natural and Synthetic 3,3´-biisofraxidin (2).

Position	δ_C in ppm		
	Natural Product	Synthetic product	$\Delta_{(S-N)}$
2,2´	159.3	159.9	0.6
3,3´	117.3	117.3	0
4,4´	143.4	143.4	0
5,5´	104.7	104.7	0
6,6´	145.9	145.9	0
7,7´	144.4	144.4	0
8,8´	134.5	134.5	0
9,9´	142.5	142.4	-0.1
10,10´	110.2	110.2	0
6,6´-OCH_3	60.8	60.9	0.1
8,8´-OCH_3	56.2	56.2	0
7,7´-OH			

2.4.1 Comparison of the NMR Data of Natural and Synthetic Biscopoletin (3).

Table S5. Comparison of the ^{1}H NMR Data of Natural and Synthetic Biscopoletin (3).

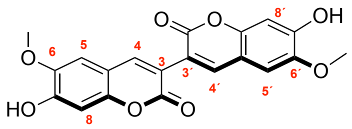

Biscopoletin (**3**)

**Position**	**δ_H in ppm (mult, *J* in Hz)**		
	**Natural Product[3]**	**Synthetic product**	**$\Delta_{(S-N)}$**
2,2´			
3,3´			
4,4´	8.29 *s*	*8.30 s*	0.01
5,5´	7.29 *s*	*7.30 s*	0.01
6,6´			
7,7´			
8,8´	6.83 *s*	*6.84 s*	0.01
9,9´			
10,10´			
6,6´-OCH_3	3.83 *s*	3.84 *s*	0.01
7,7´-OH	10.45 *s*	10.45 *s*	0

Table S6. Comparison of the ^{13}C NMR Data of Natural and Synthetic Biscopoletin (3).

**Position**	**δ_C in ppm**		
	**Natural Product**	**Synthetic product**	**$\Delta_{(S-N)}$**
2,2´	159.7	159.8	0.1
3,3´	117.0	117.1	0.1
4,4´	143.1	143.0	-0.1
5,5´	109.7	109.8	0.1
6,6´	145.6	145.6	0
7,7´	151.6	151.7	0.1
8,8´	102.5	102.5	0
9,9´	149.0	149.1	0.1
10,10´	110.6	110.6	0
6,6´-OCH_3	56.1	56.1	0
7,7´-OH			

3. NMR spectra

^{1}H NMR (400 MHz, $CDCl_3$) and **^{13}C NMR** (100 MHz, $CDCl_3$) for compound **7a.**

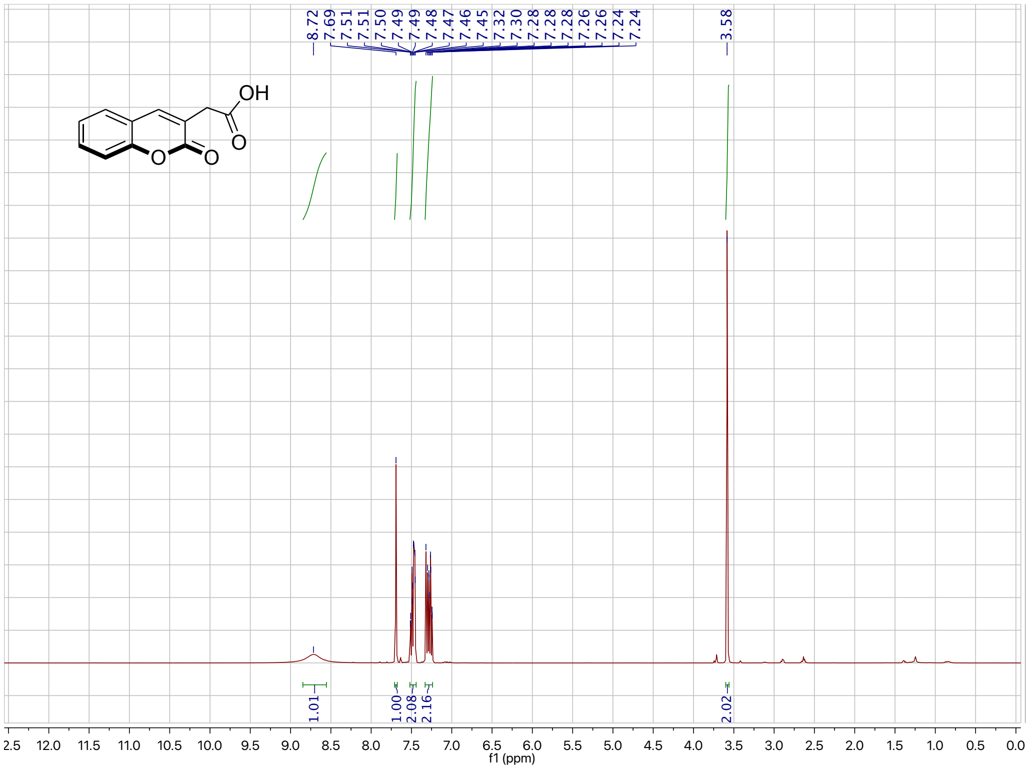

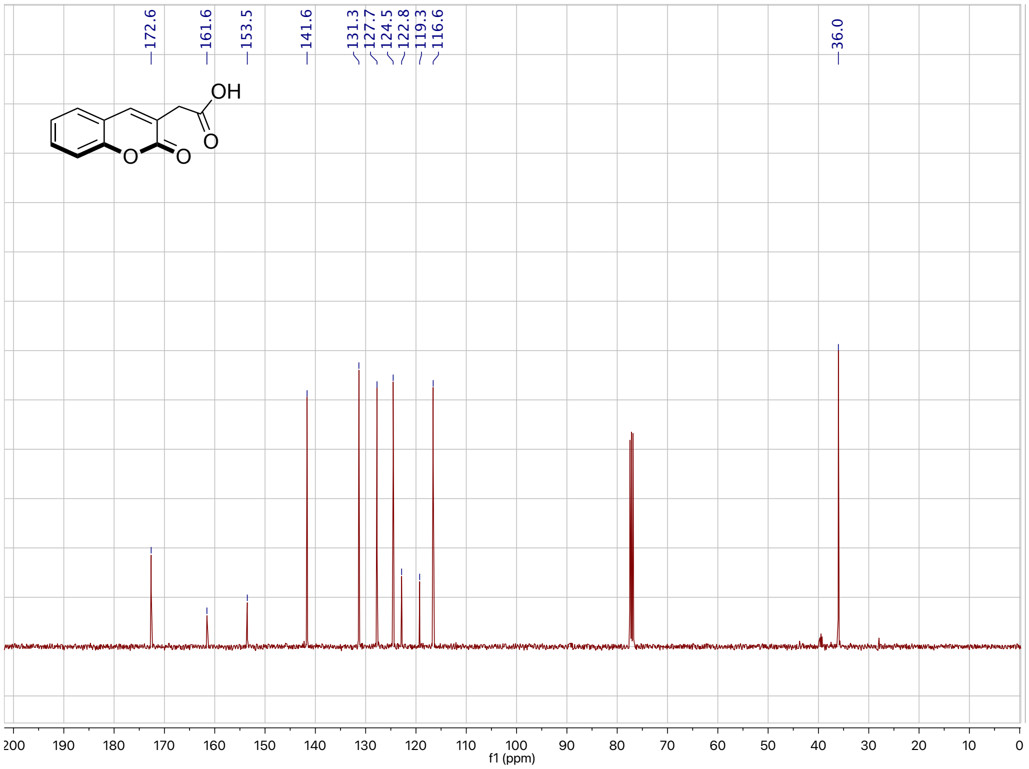

^{1}**H NMR** (400 MHz, DMSO-d_6) **and** ^{13}**C NMR** (100 MHz, DMSO-d_6) for compound **7b.**

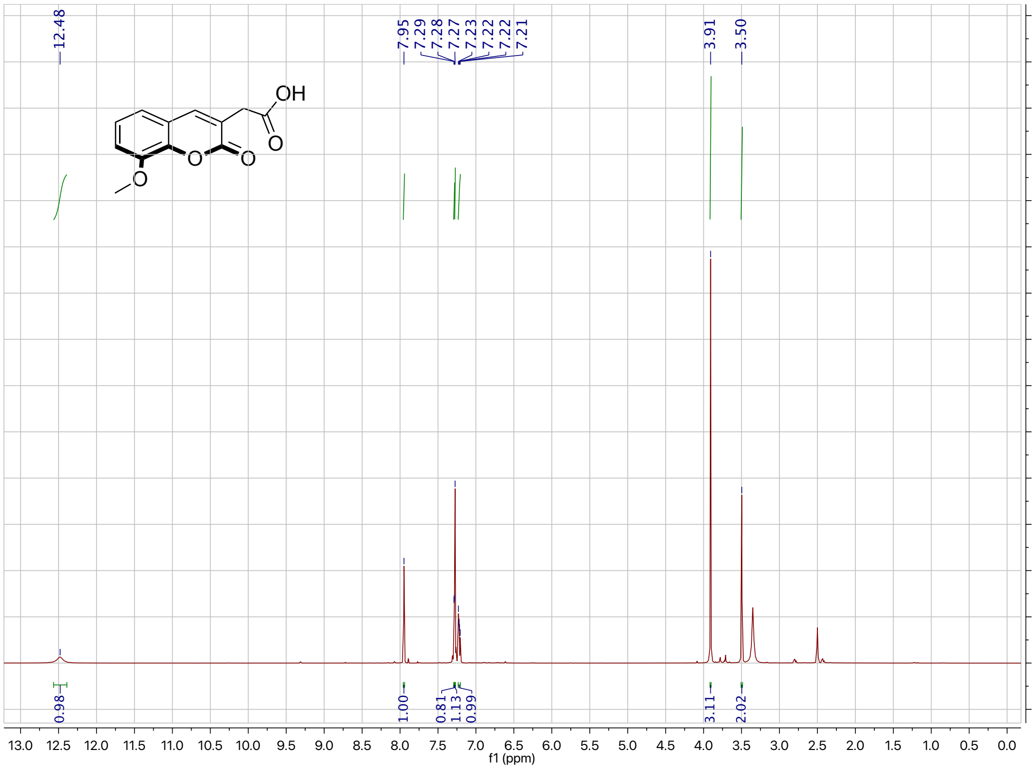

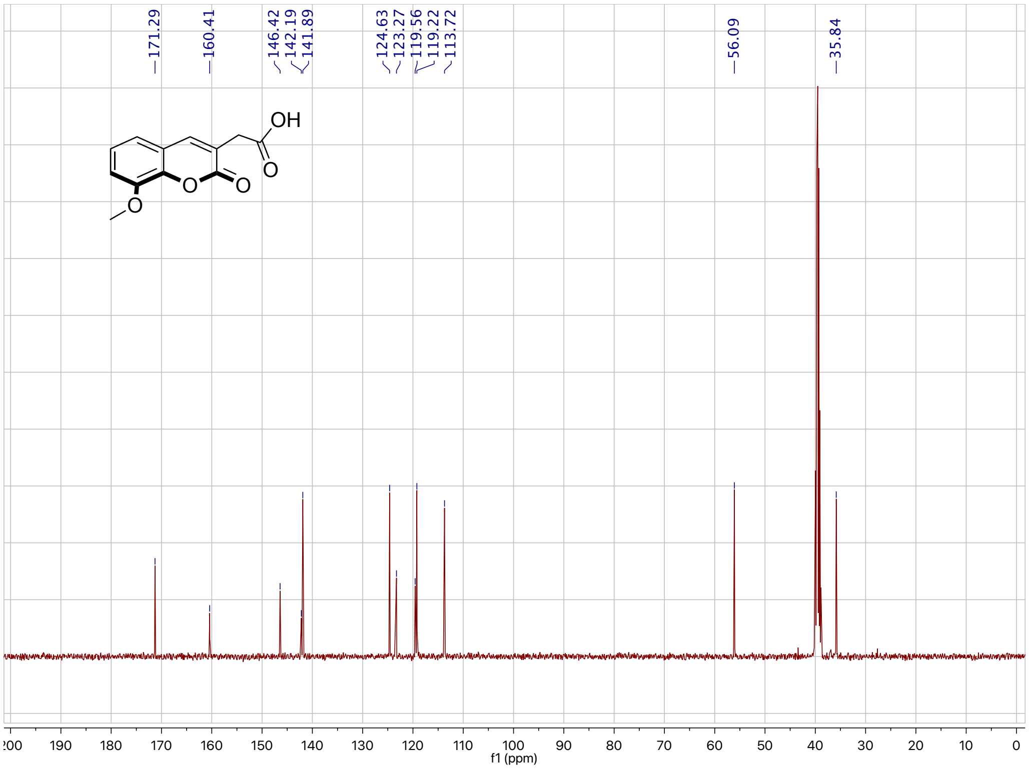

^{1}H NMR (400 MHz, DMSO-d_6) **and ^{13}C NMR** (100 MHz, DMSO-d_6) for compound **7c.**

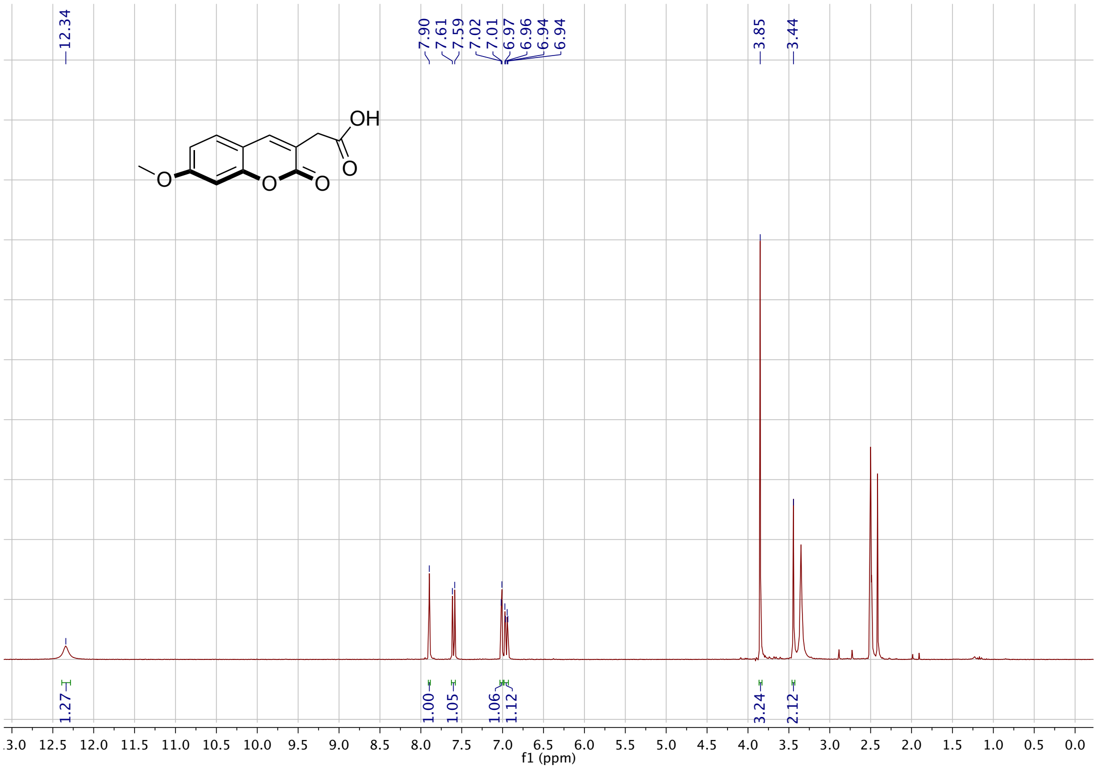

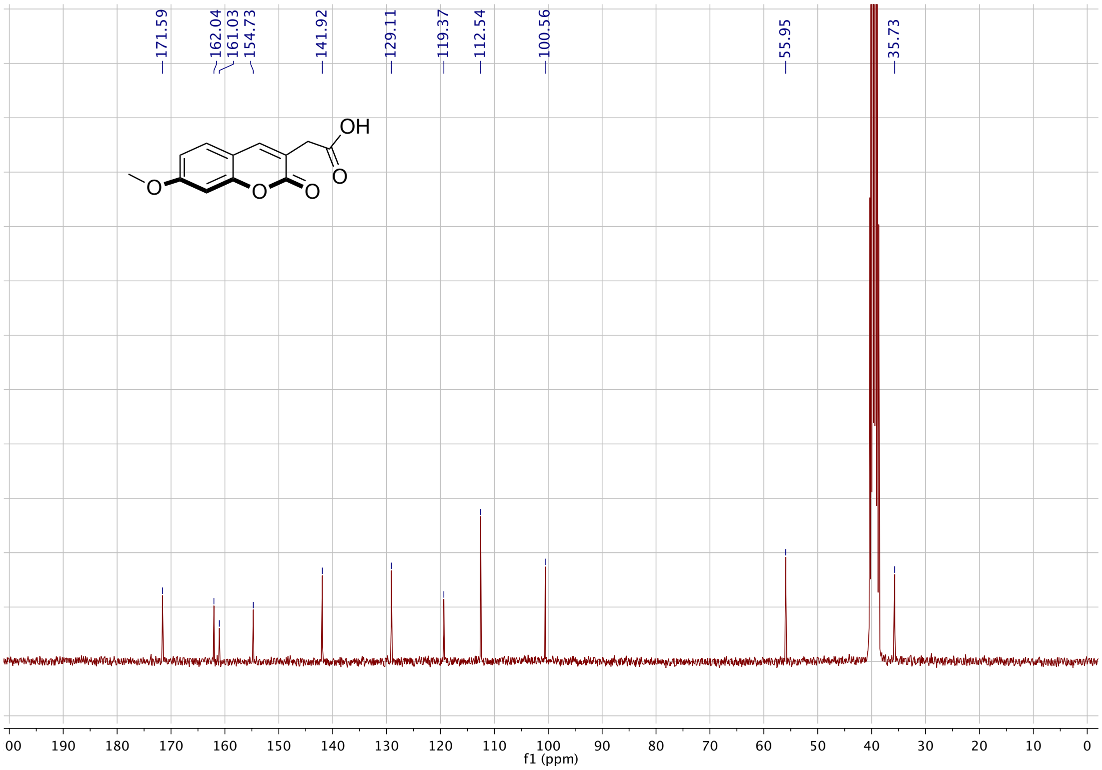

^{1}**H NMR** (400 MHz, DMSO-d_6) **and** ^{13}**C NMR** (100 MHz, DMSO-d_6) for compound **7d.**

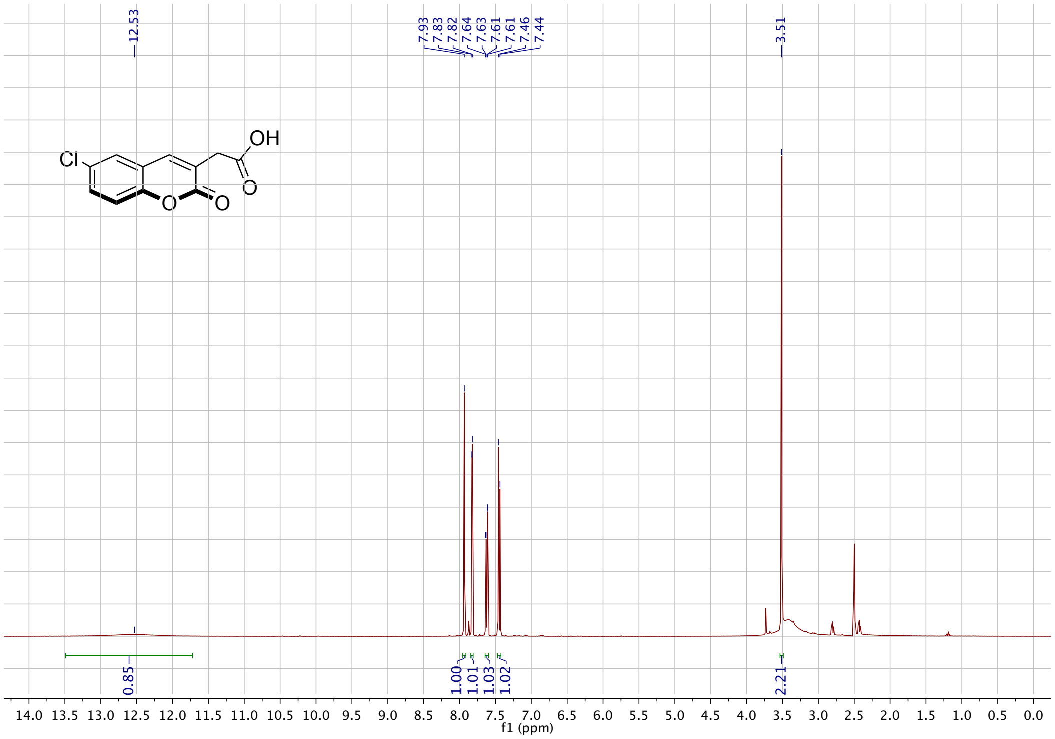

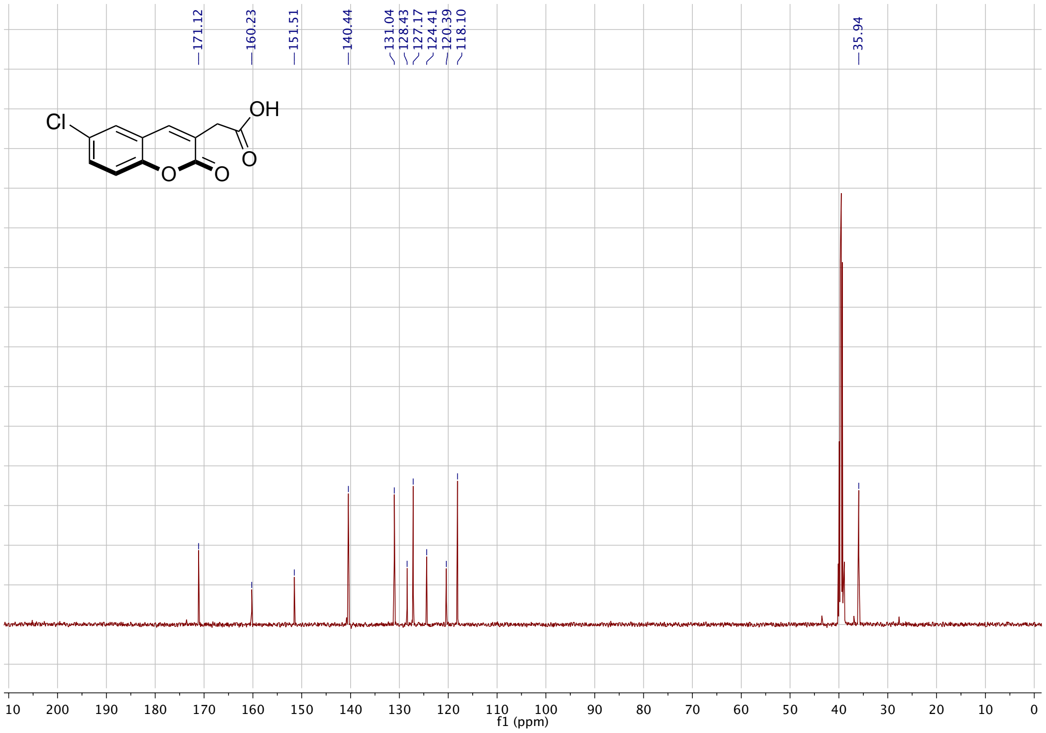

^{1}H NMR (400 MHz, DMSO-d_6) **and ^{13}C NMR** (100 MHz, DMSO-d_6) for compound **7e.**

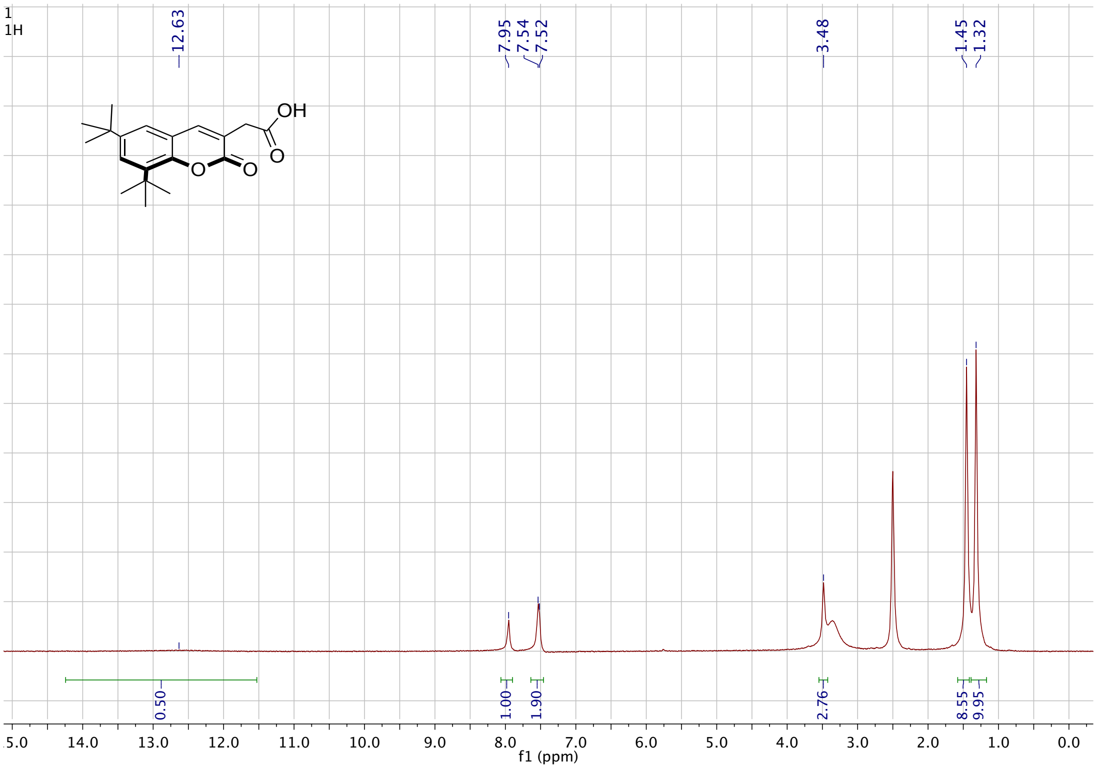

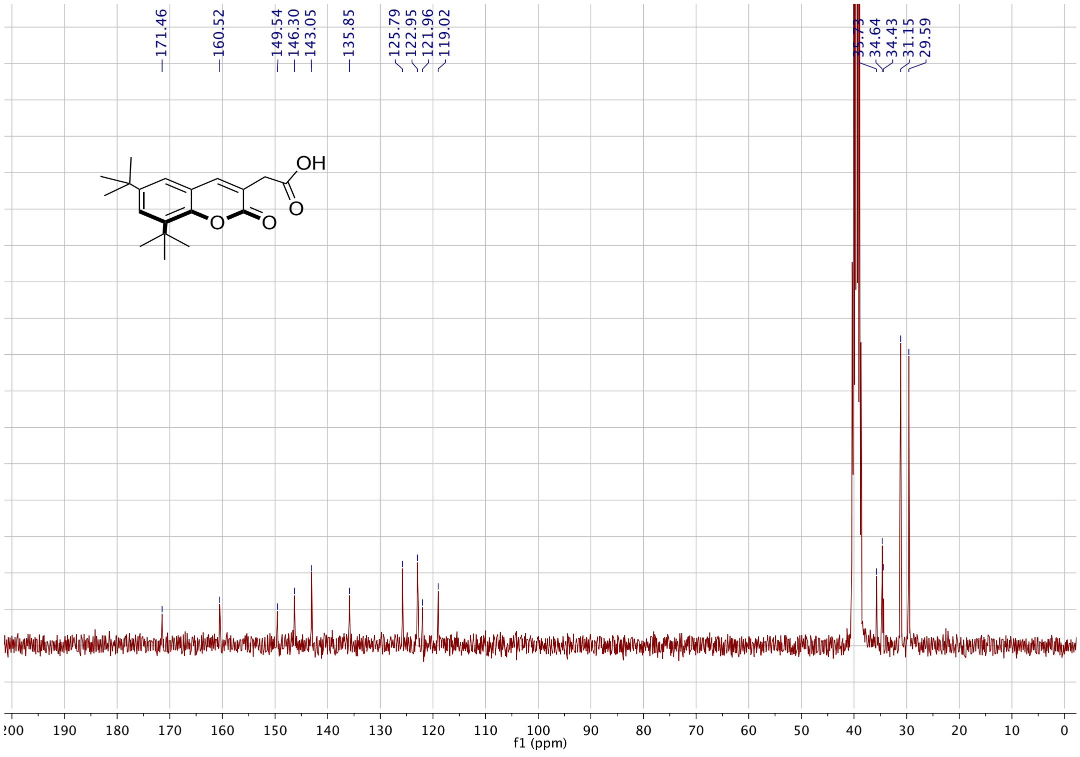

^{1}H NMR (300 MHz, DMSO-d_6) **and ^{13}C NMR** (75 MHz, DMSO-d_6) for compound **7f.**

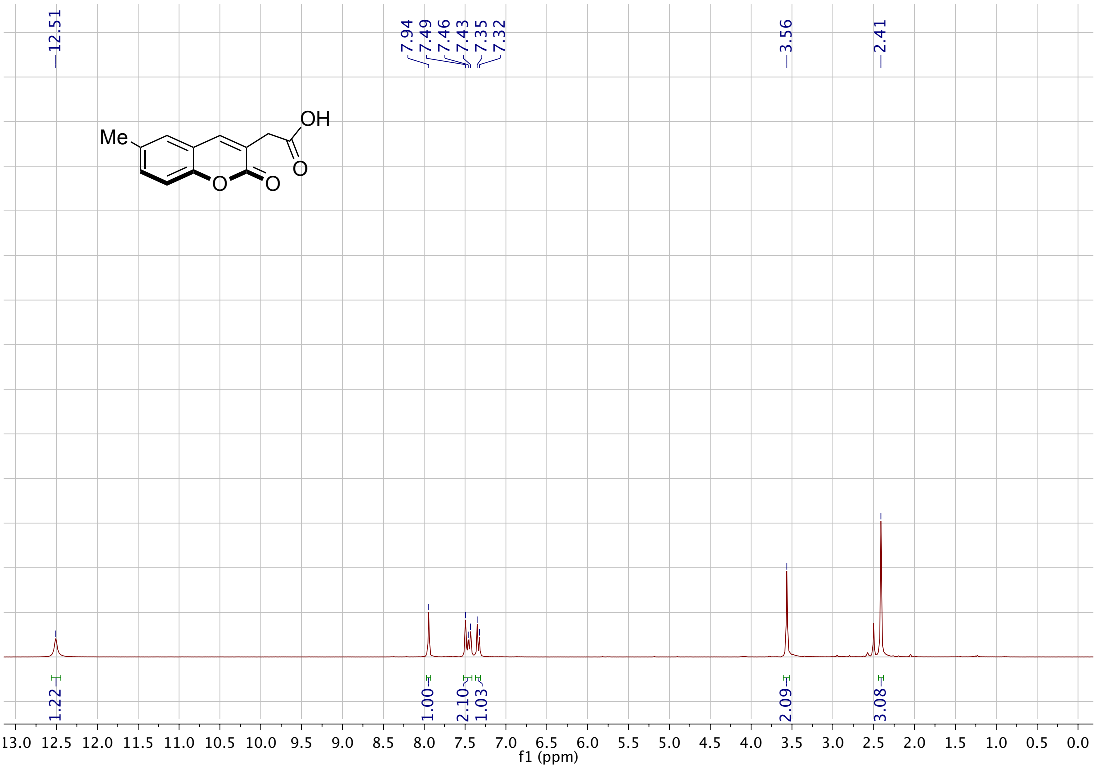

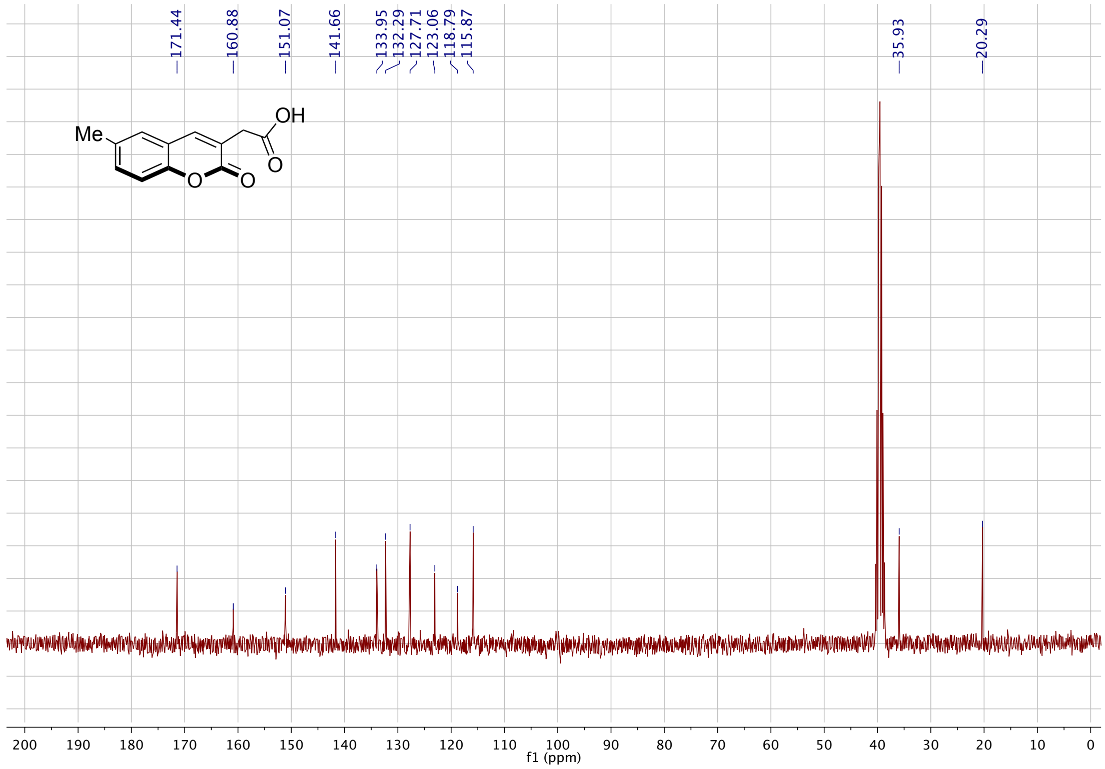

^{1}H NMR (300 MHz, DMSO-d_6) **and ^{13}C NMR** (75 MHz, DMSO-d_6) for compound **7g.**

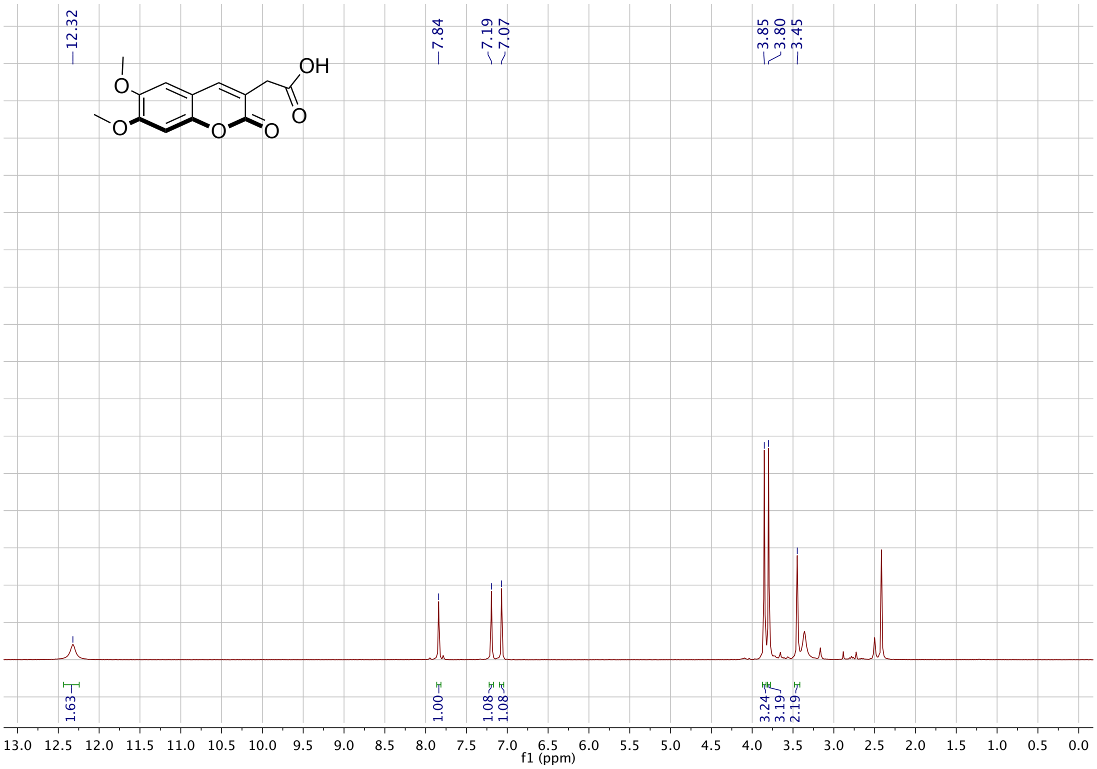

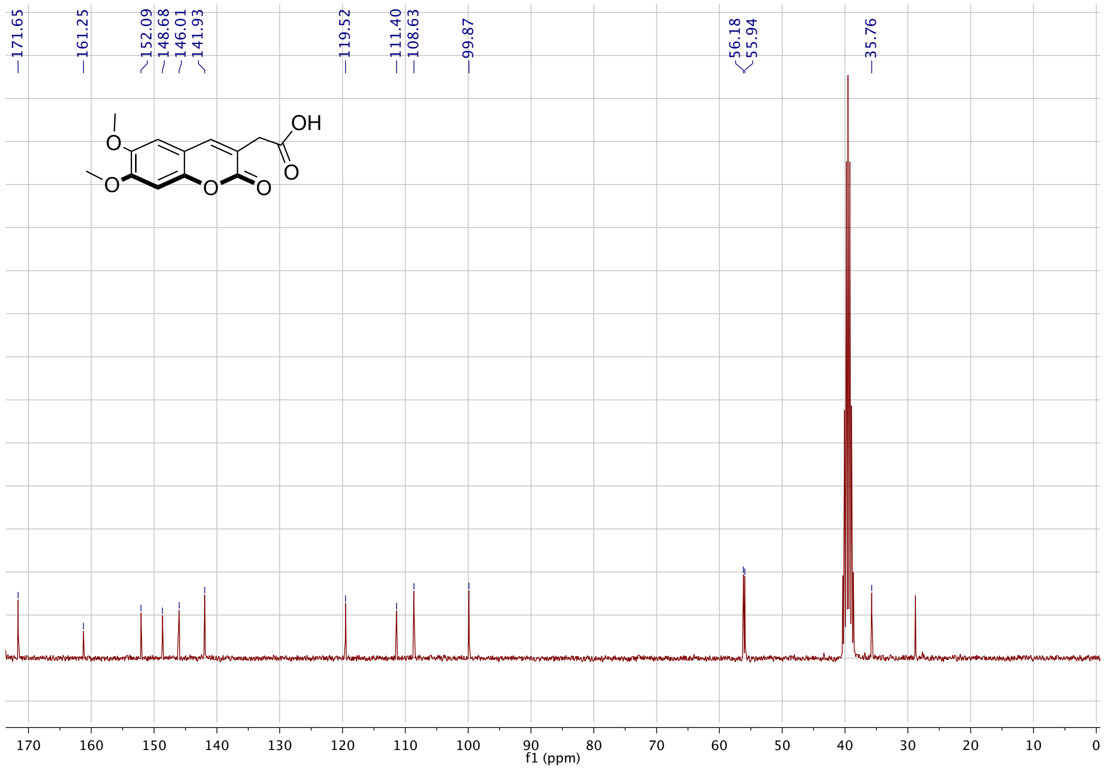

[1]**H NMR** (300 MHz, DMSO-d_6) and [13]**C NMR** (75 MHz, DMSO-d_6) for compound **7h.**

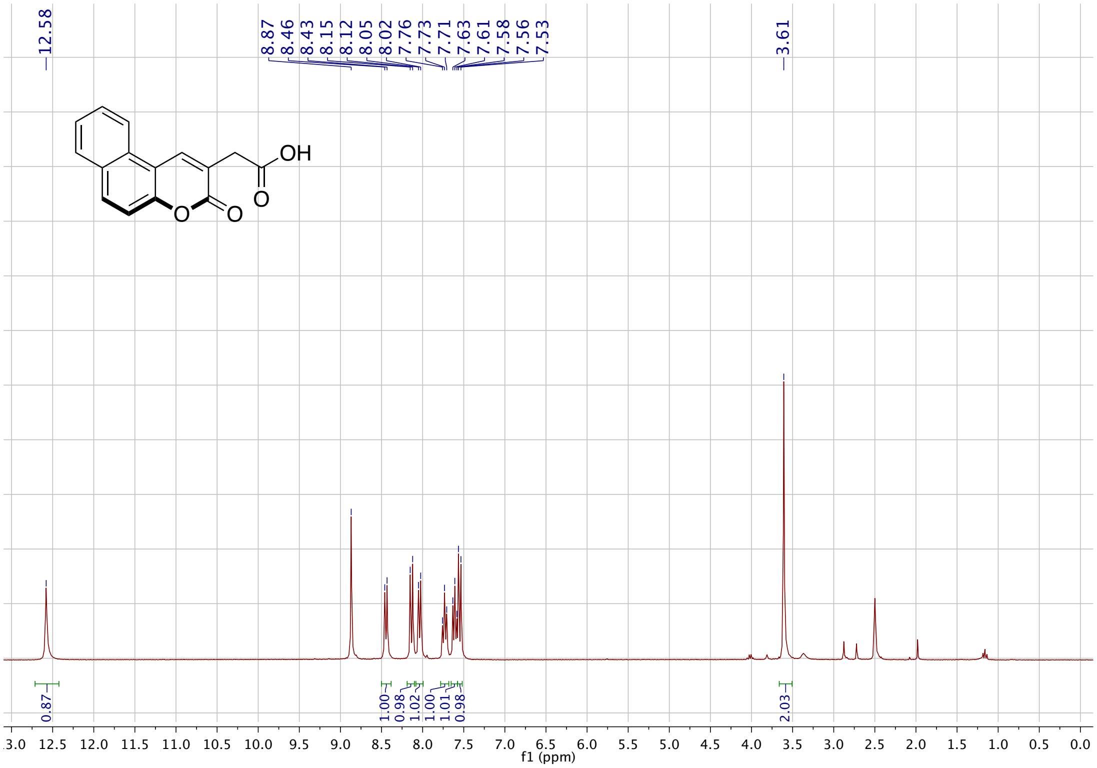

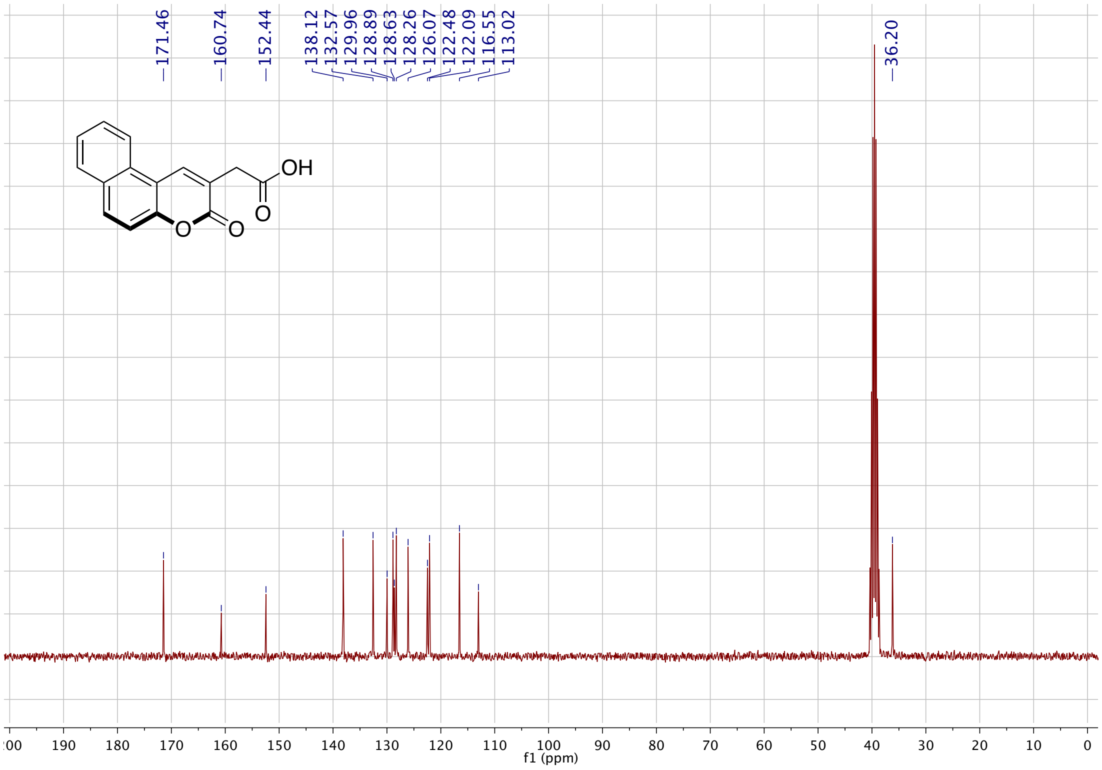

^{1}H NMR (300 MHz, $CDCl_3$) and **^{13}C NMR** (75 MHz, $CDCl_3$) for compound **8i.**

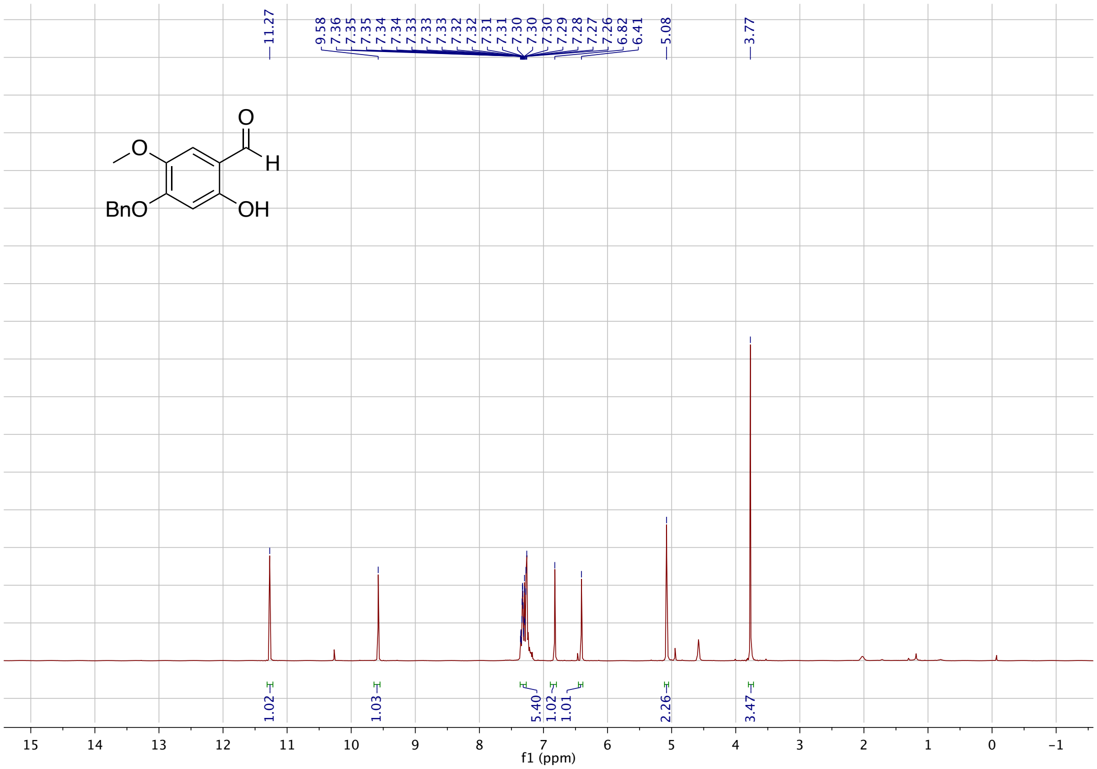

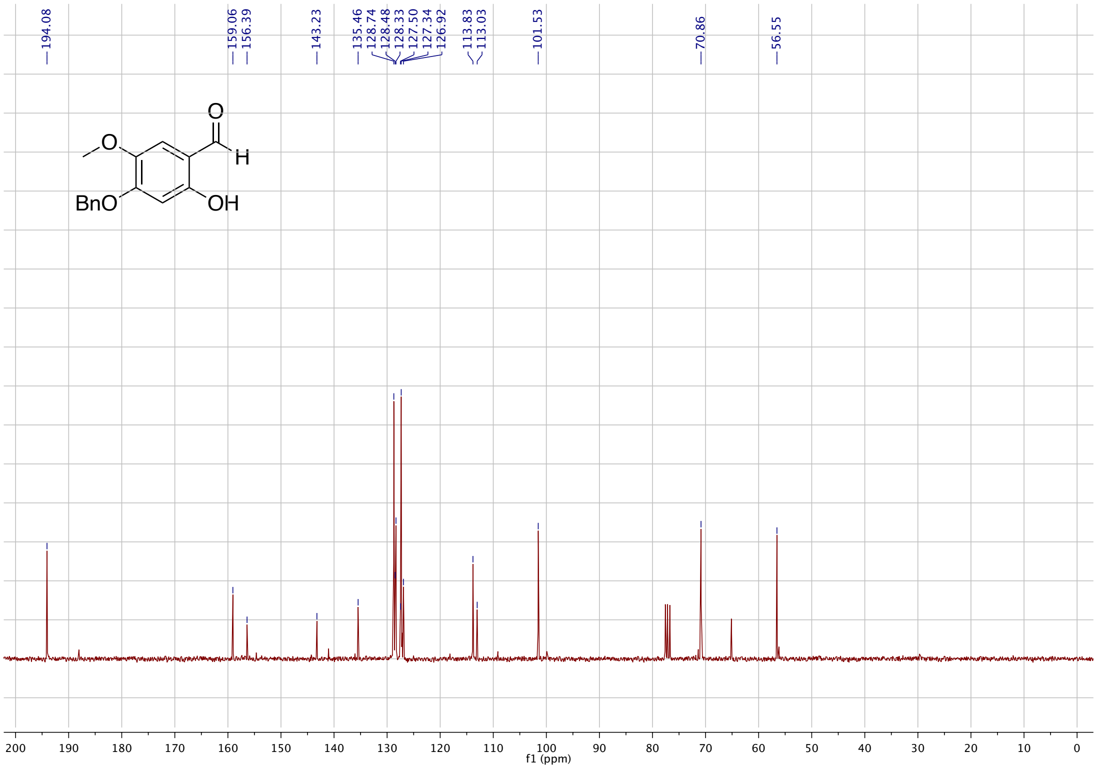

^{1}H NMR (300 MHz, DMSO-d_6) and **^{13}C NMR** (75 MHz, DMSO-d_6) for compound **7i.**

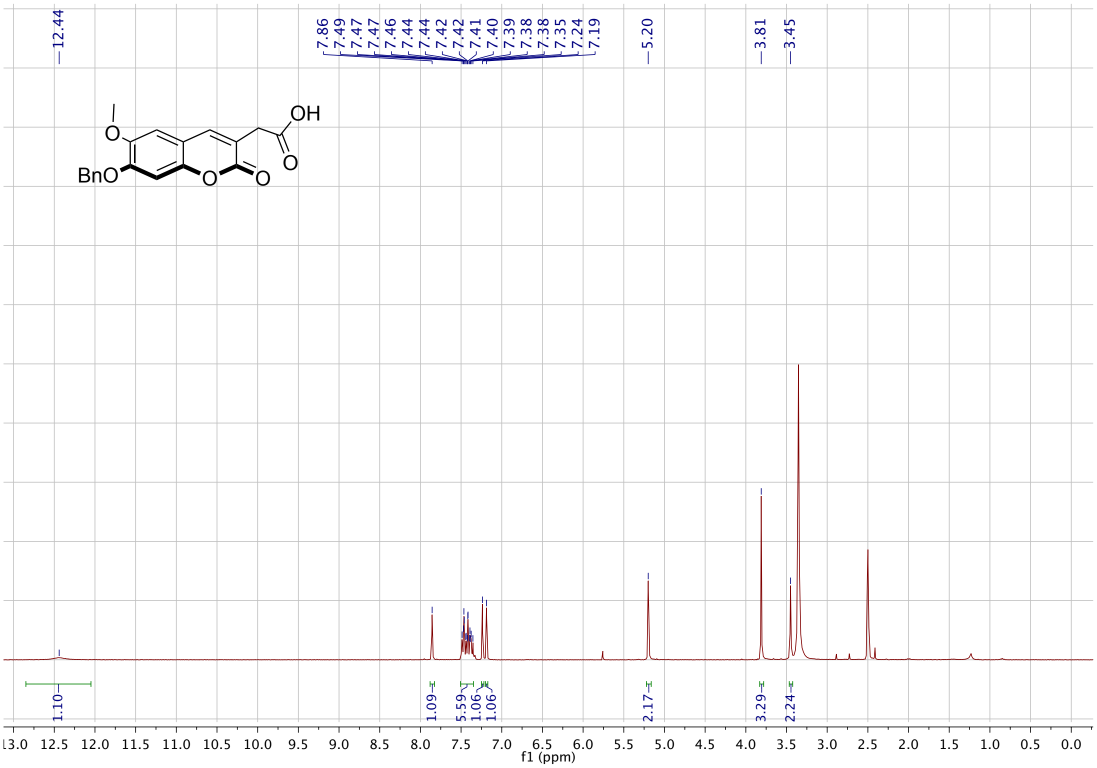

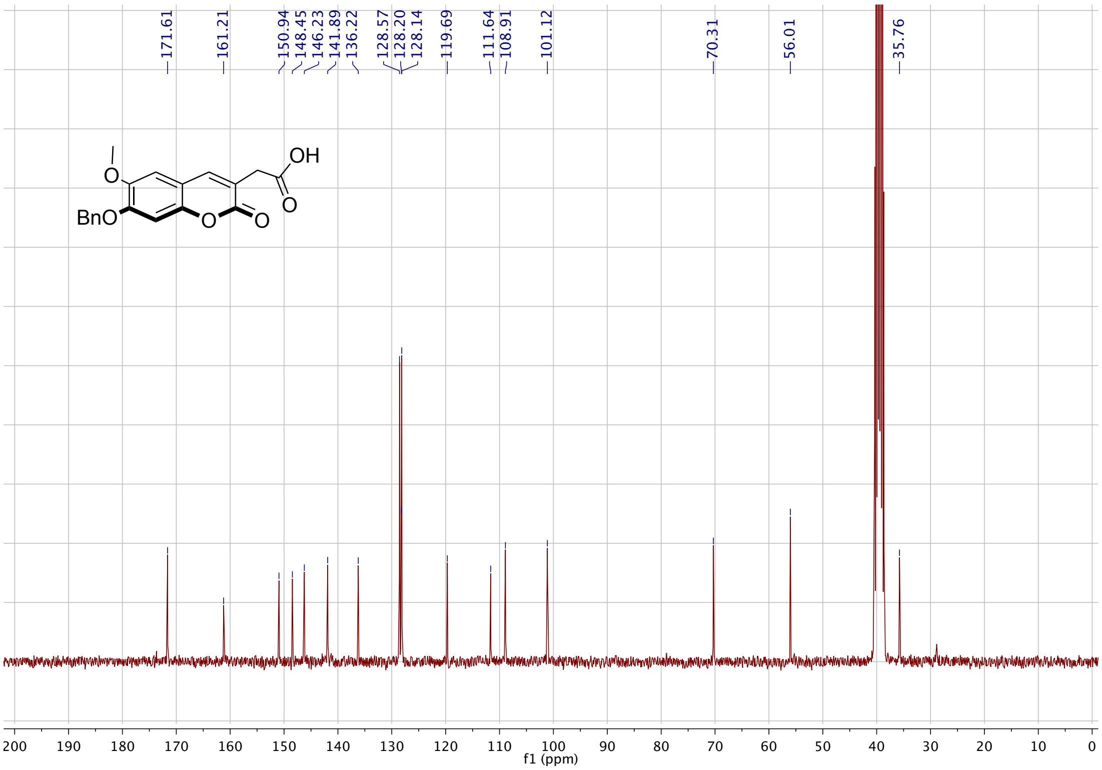

^{1}H NMR (300 MHz, $CDCl_3$) and **^{13}C NMR** (75 MHz, $CDCl_3$) for compound **S5.**

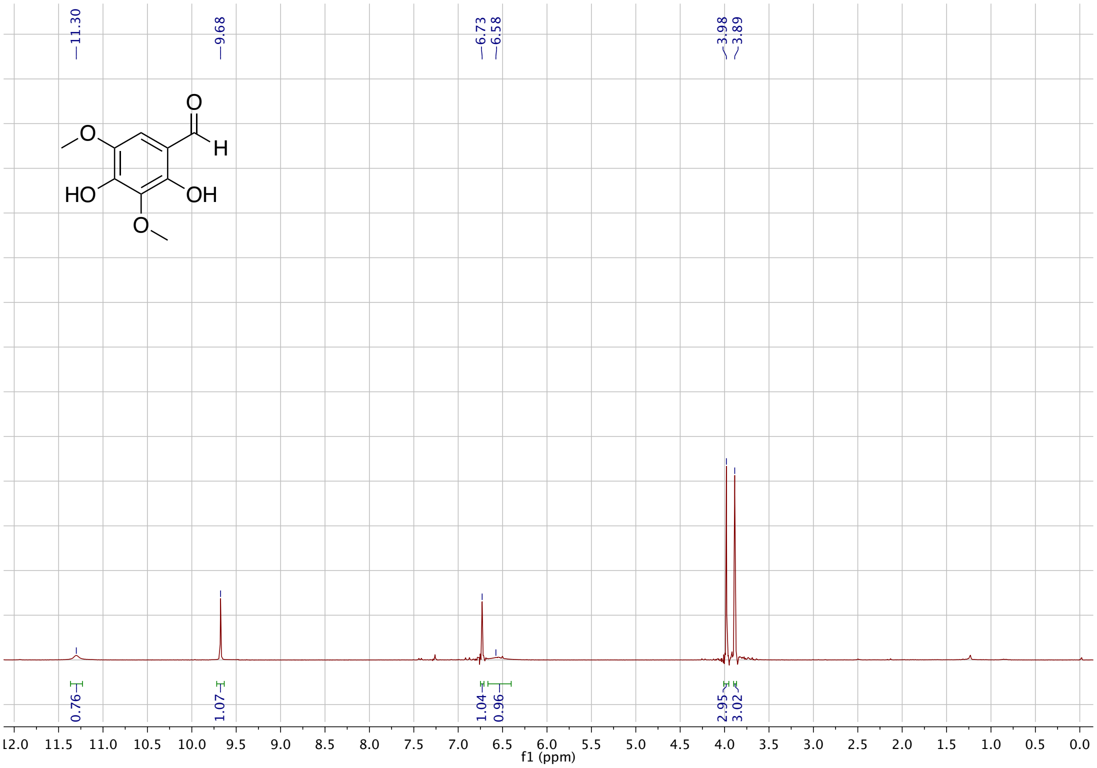

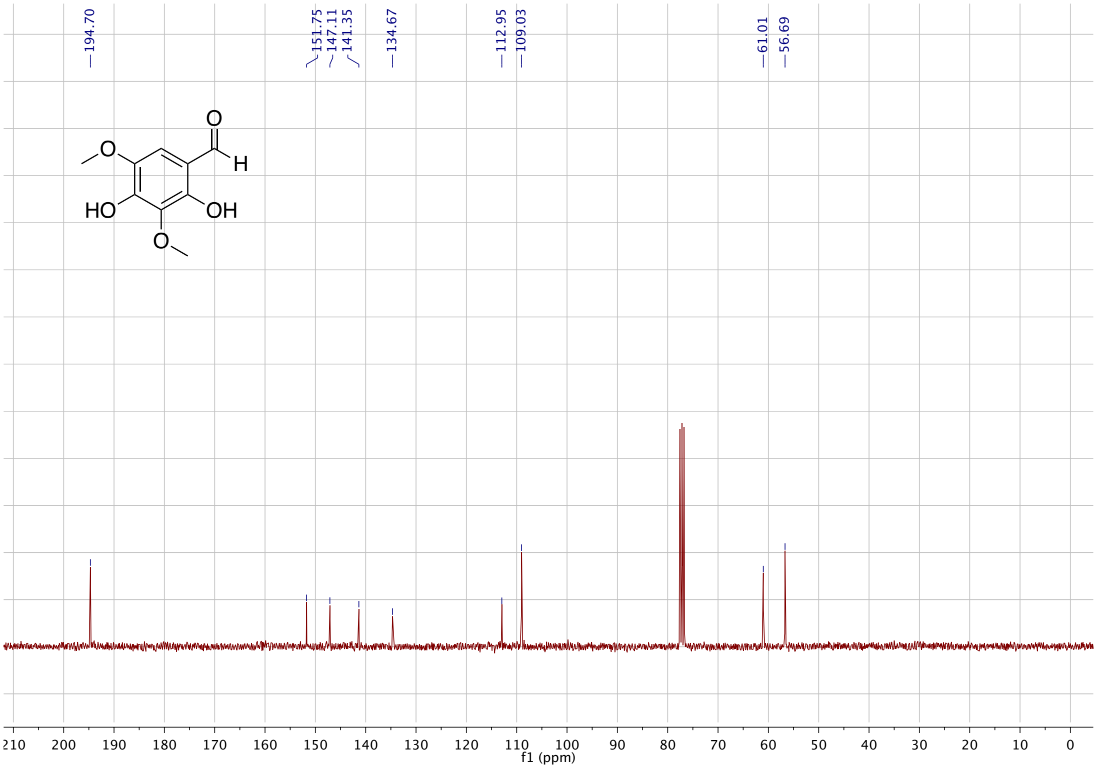

^{1}**H NMR** (300 MHz, $CDCl_3$) and ^{13}**C NMR** (75 MHz, $CDCl_3$) for compound **8j.**

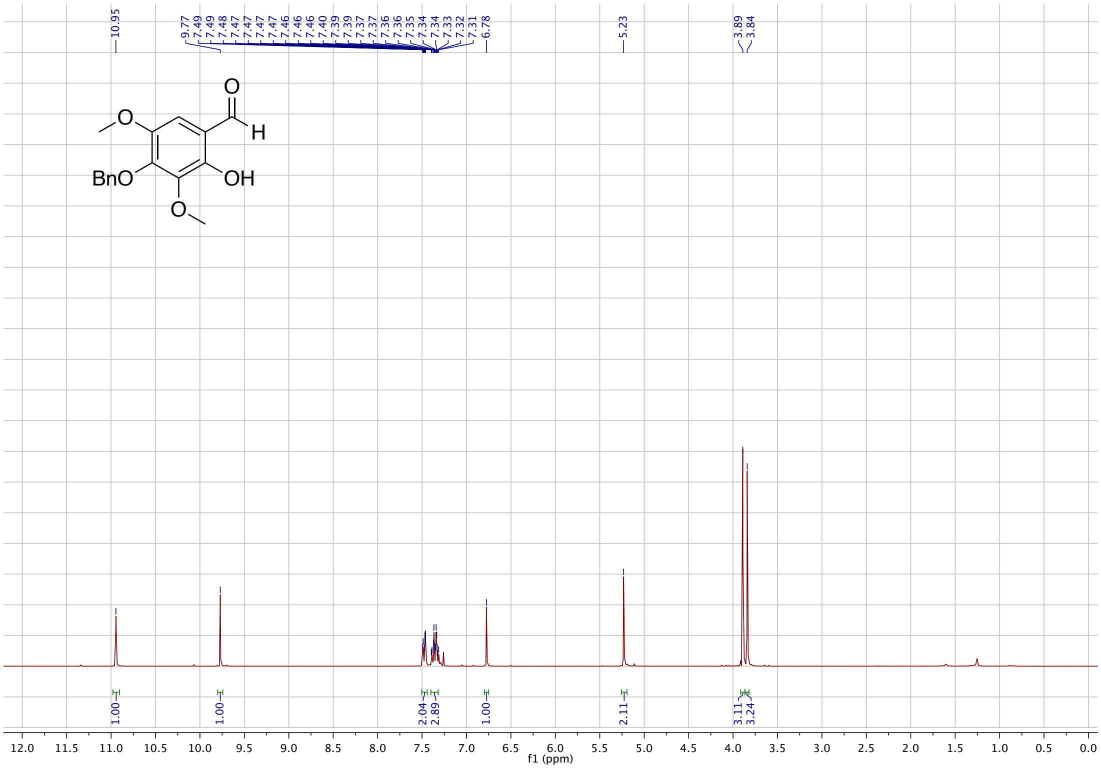

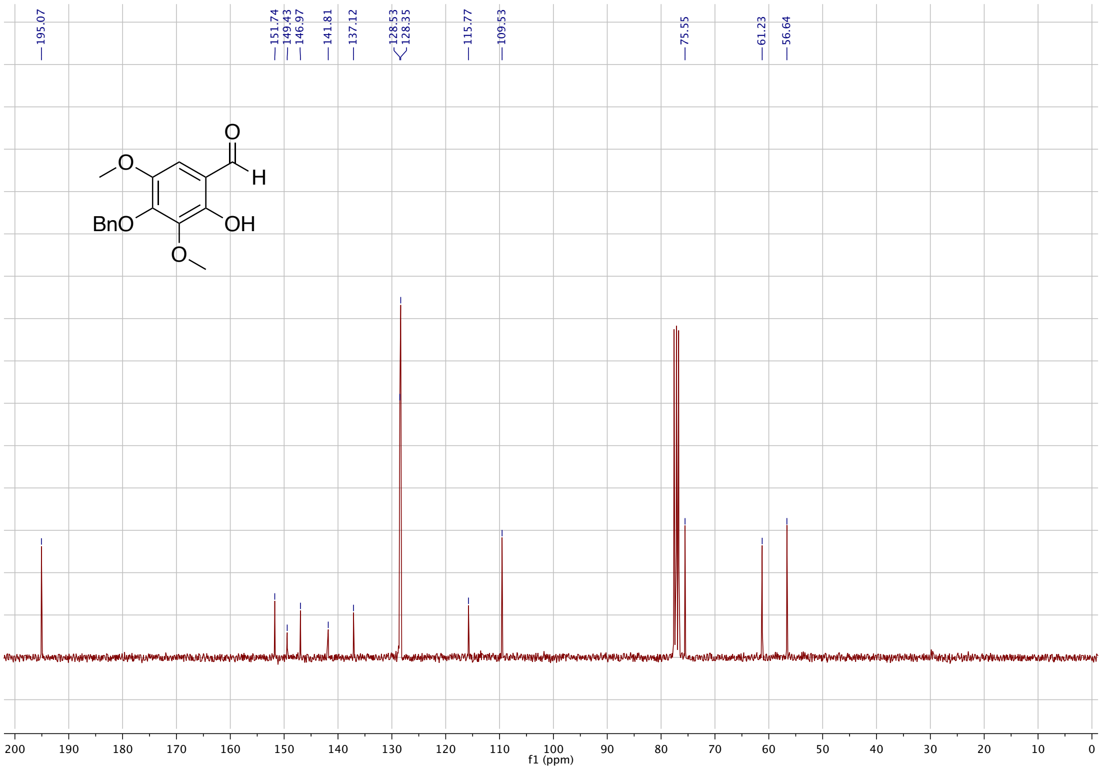

^{1}**H NMR** (400 MHz, $CDCl_3$) and ^{13}**C NMR** (100 MHz, $CDCl_3$) for compound **7j.**

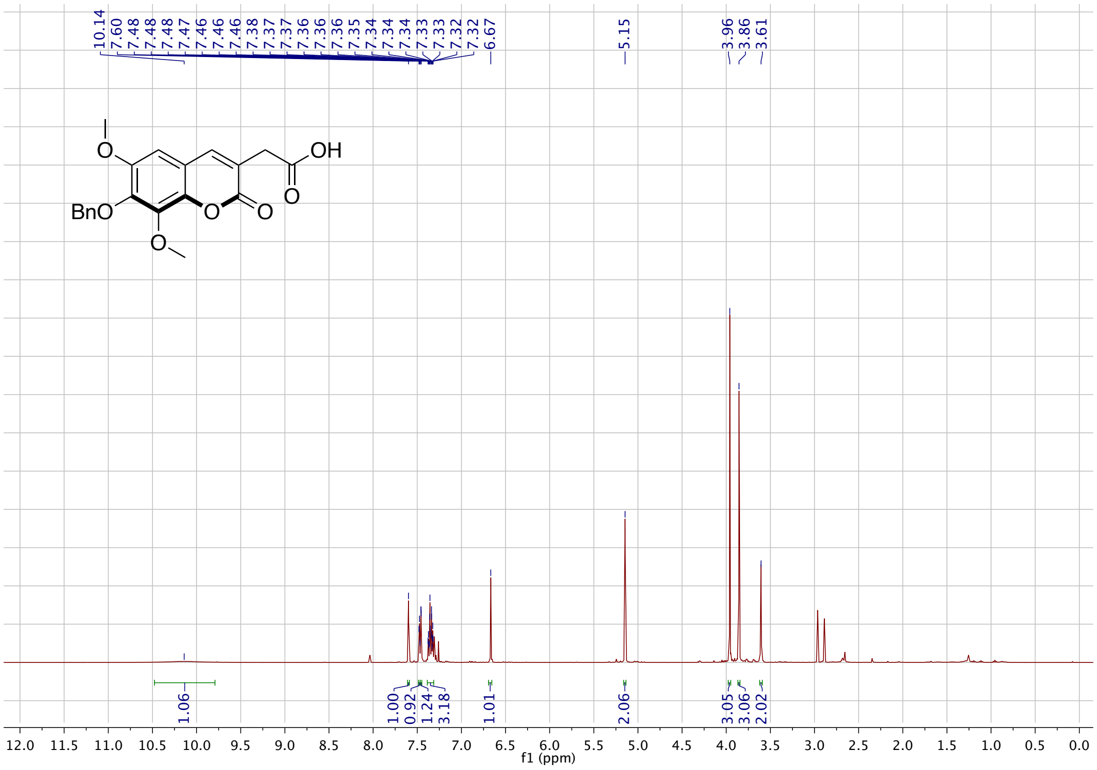

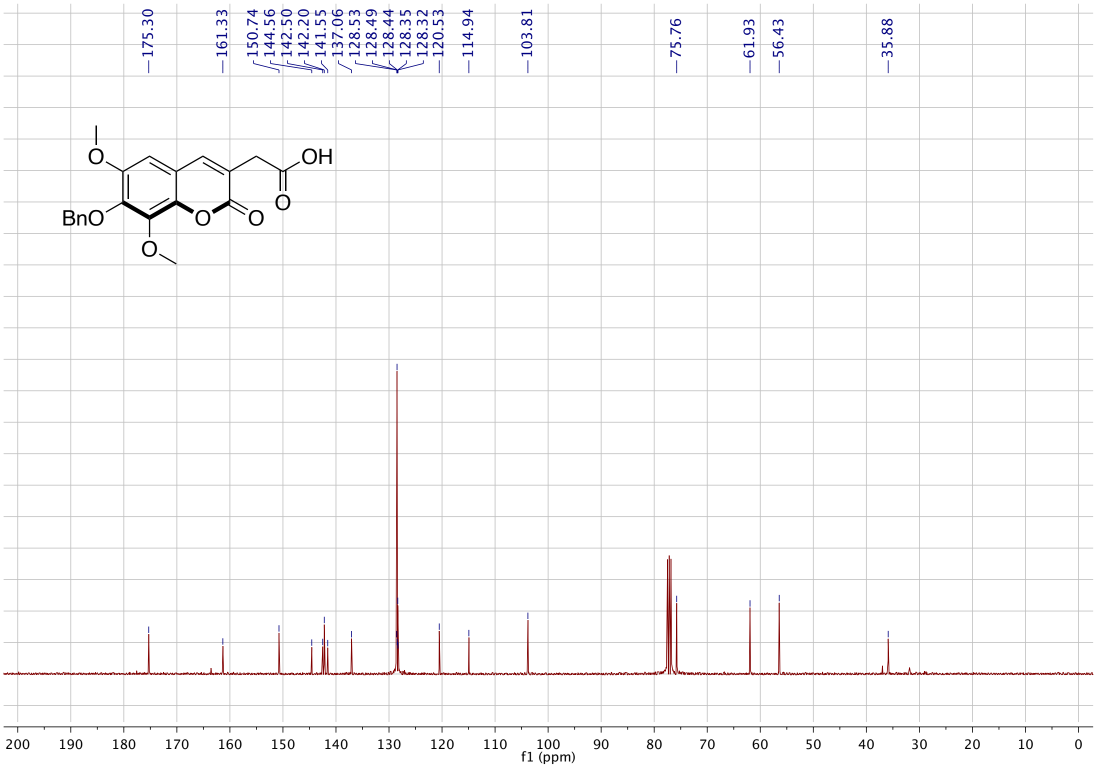

^{1}H NMR (300 MHz, $CDCl_3$) and **^{13}C NMR** (75 MHz, $CDCl_3$) for compound **8K.**

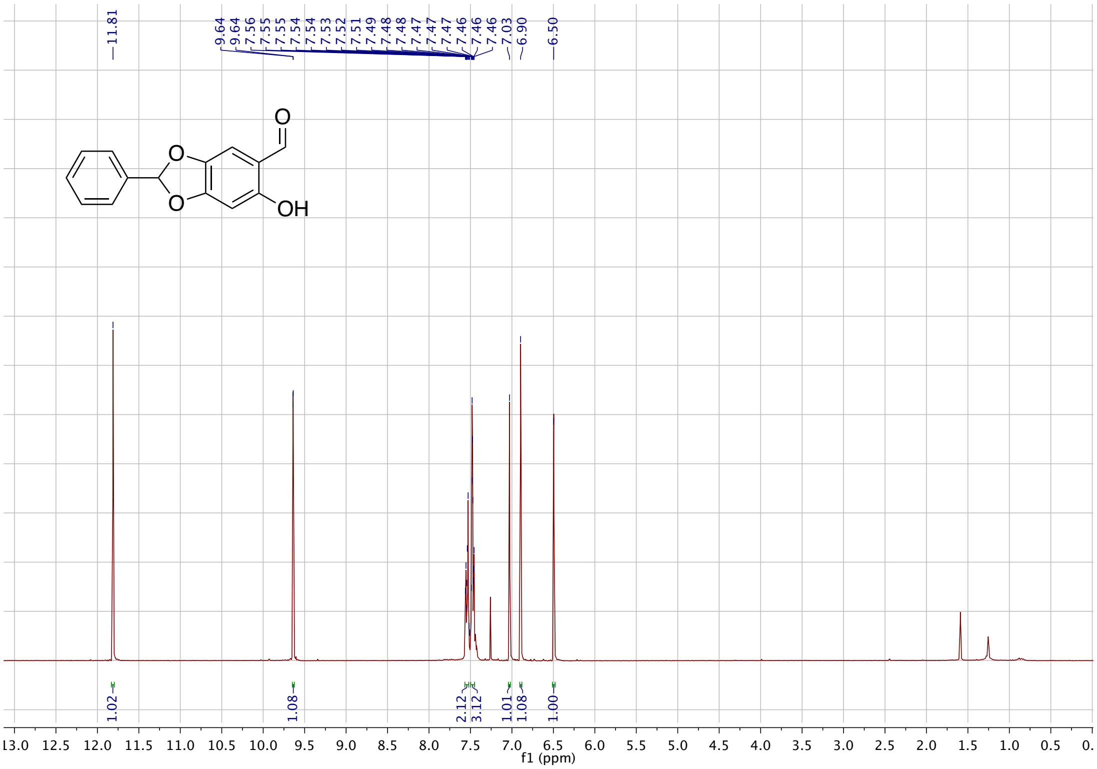

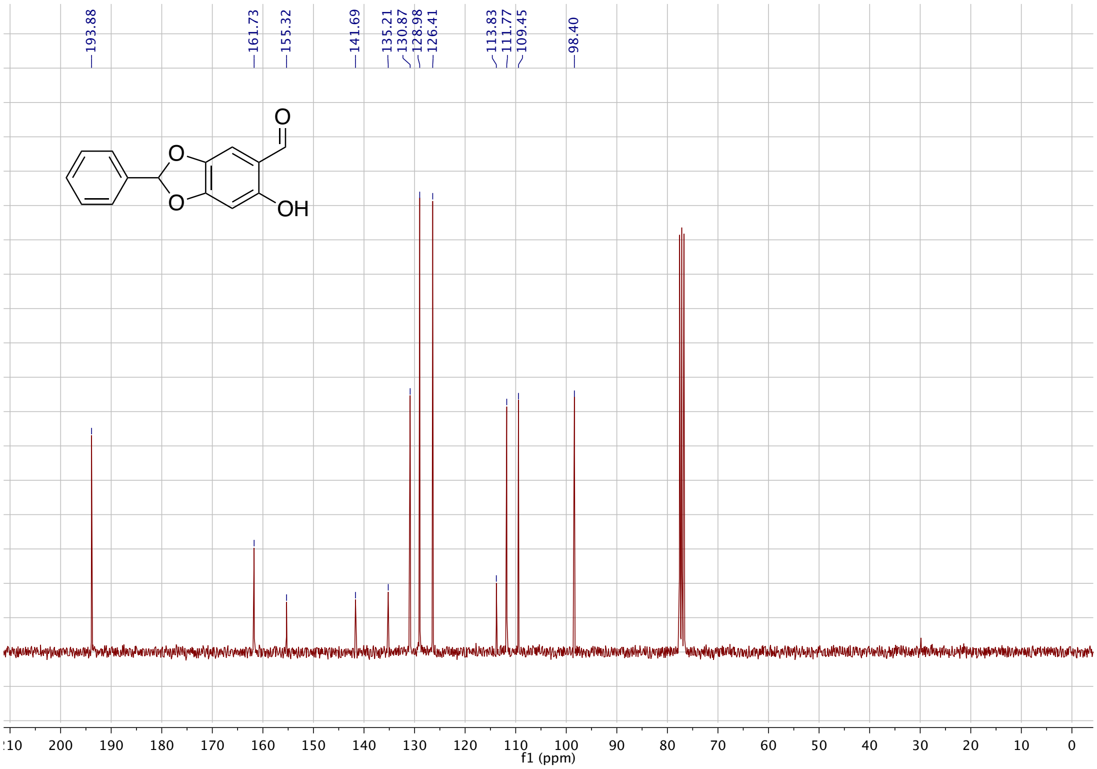

^{1}H NMR (300 MHz, $CDCl_3$) and **^{13}C NMR** (75 MHz, $CDCl_3$) for compound **9a.**

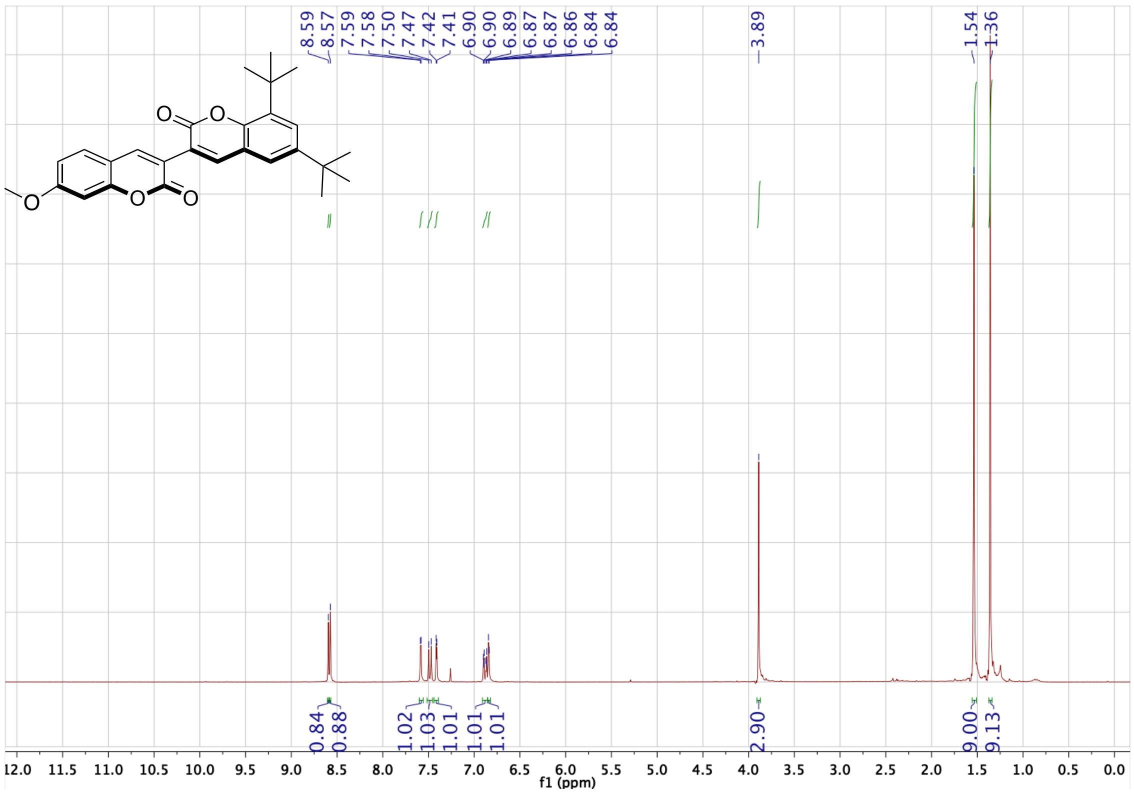

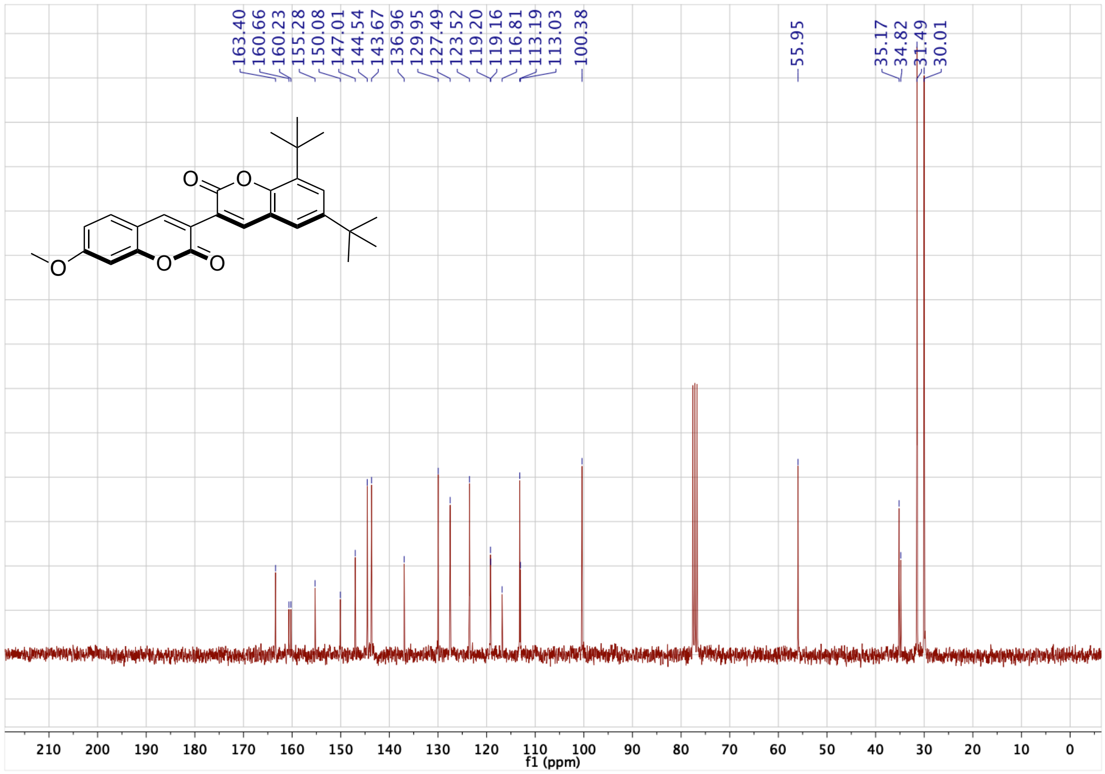

^{1}H NMR (300 MHz, $CDCl_3$) and **^{13}C NMR** (75 MHz, $CDCl_3$) for compound **9b.**

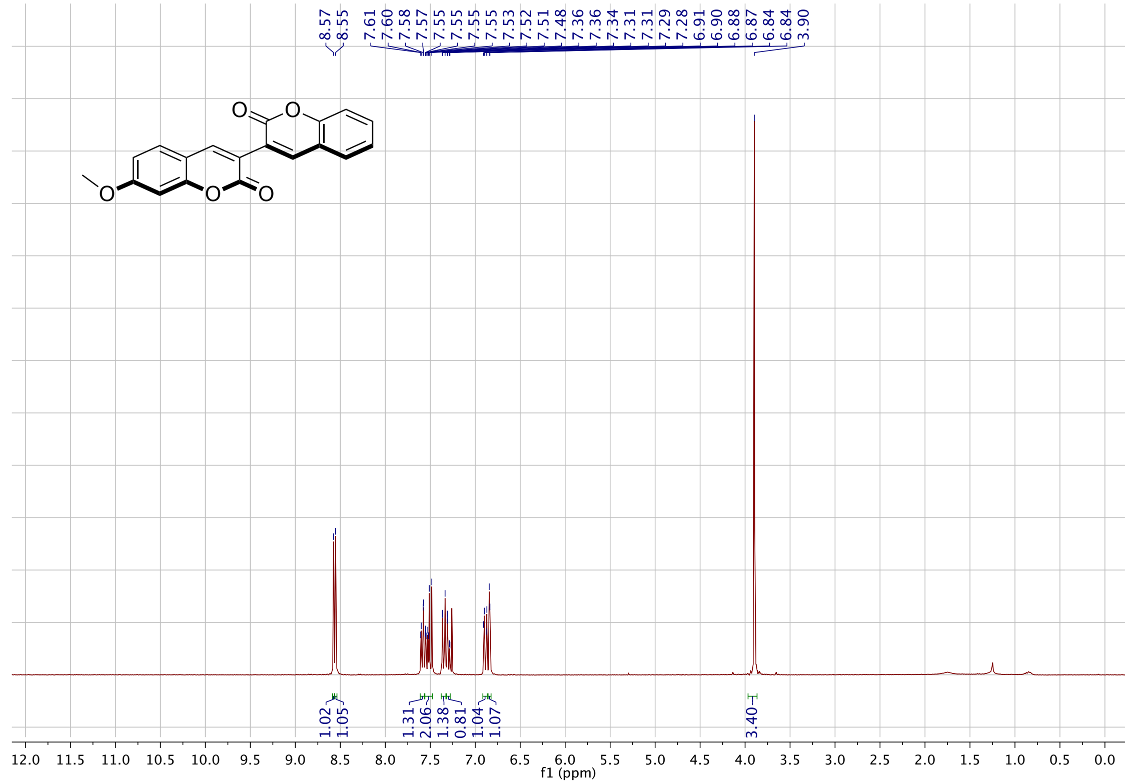

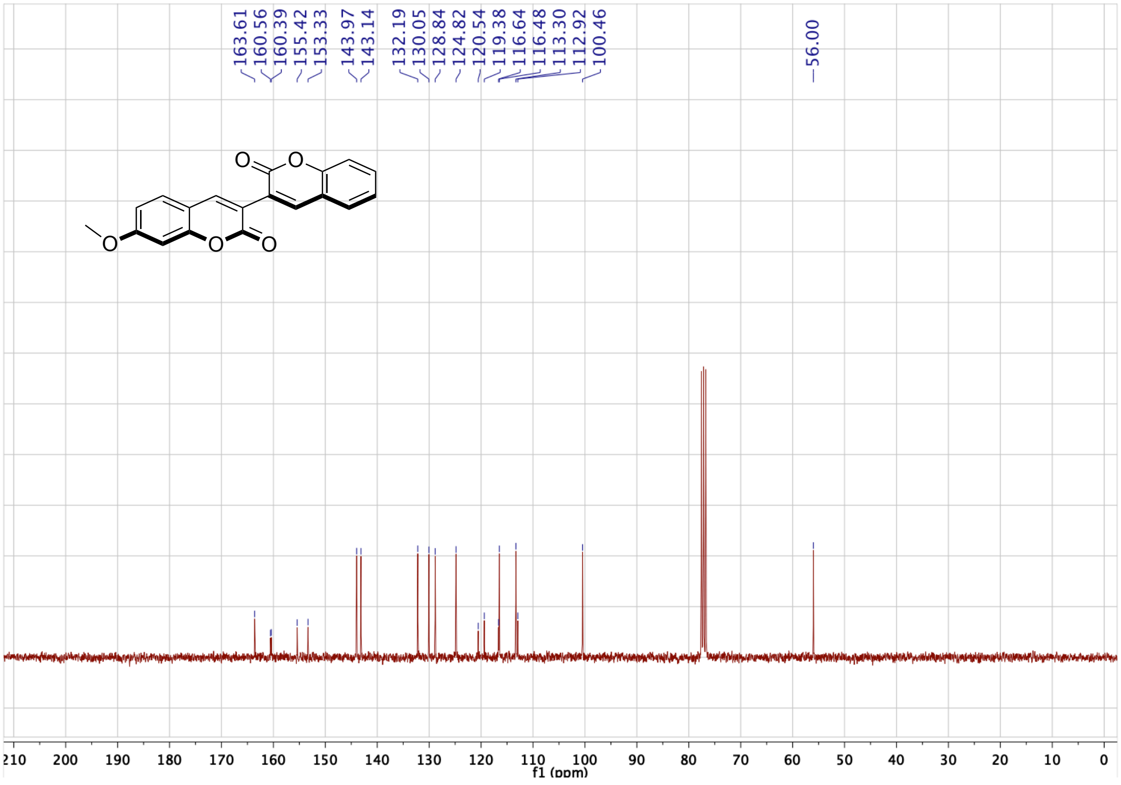

^{1}H NMR (300 MHz, $CDCl_3$) and **^{13}C CPMAS ssNMR** (125 MHz) for compound **9c.**

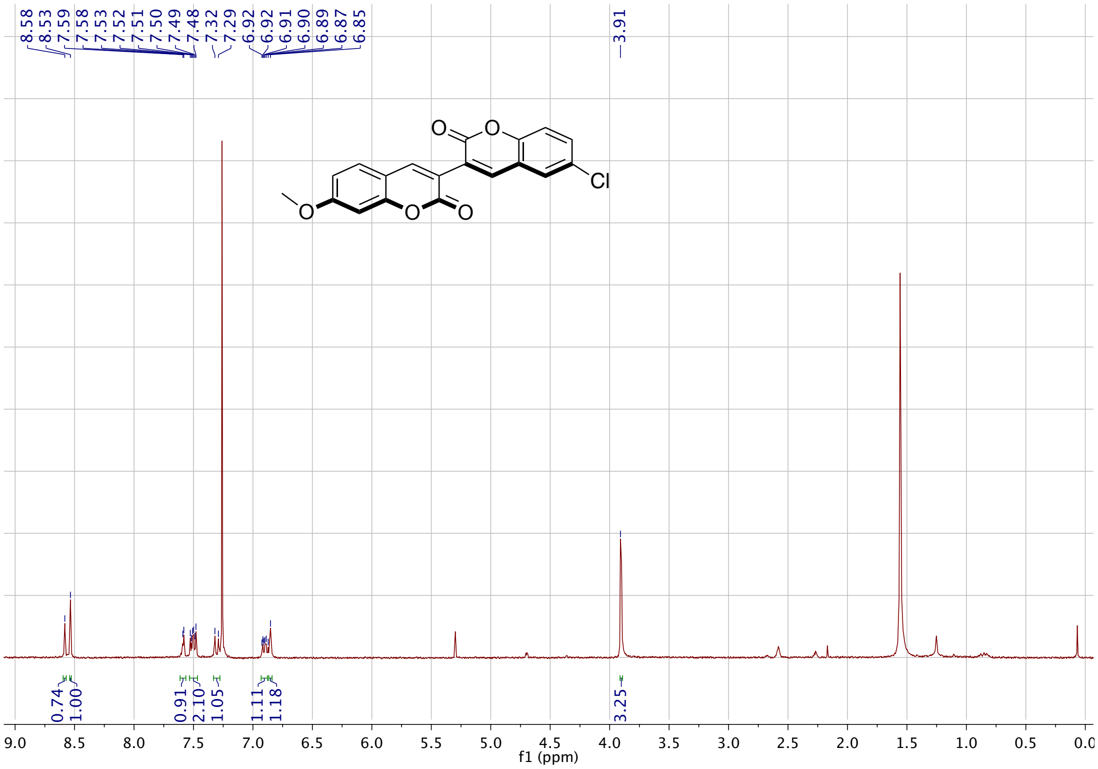

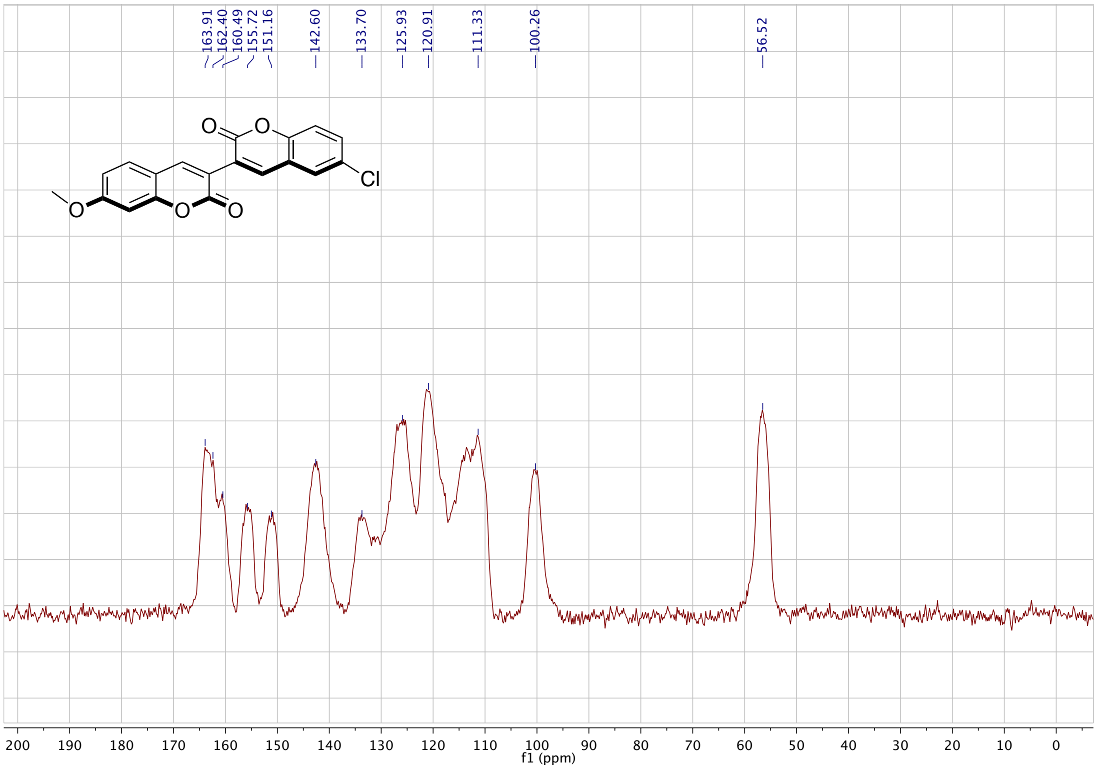

¹H NMR (300 MHz, $CDCl_3$) and **¹³C NMR** (75 MHz, $CDCl_3$) for compound **9d.**

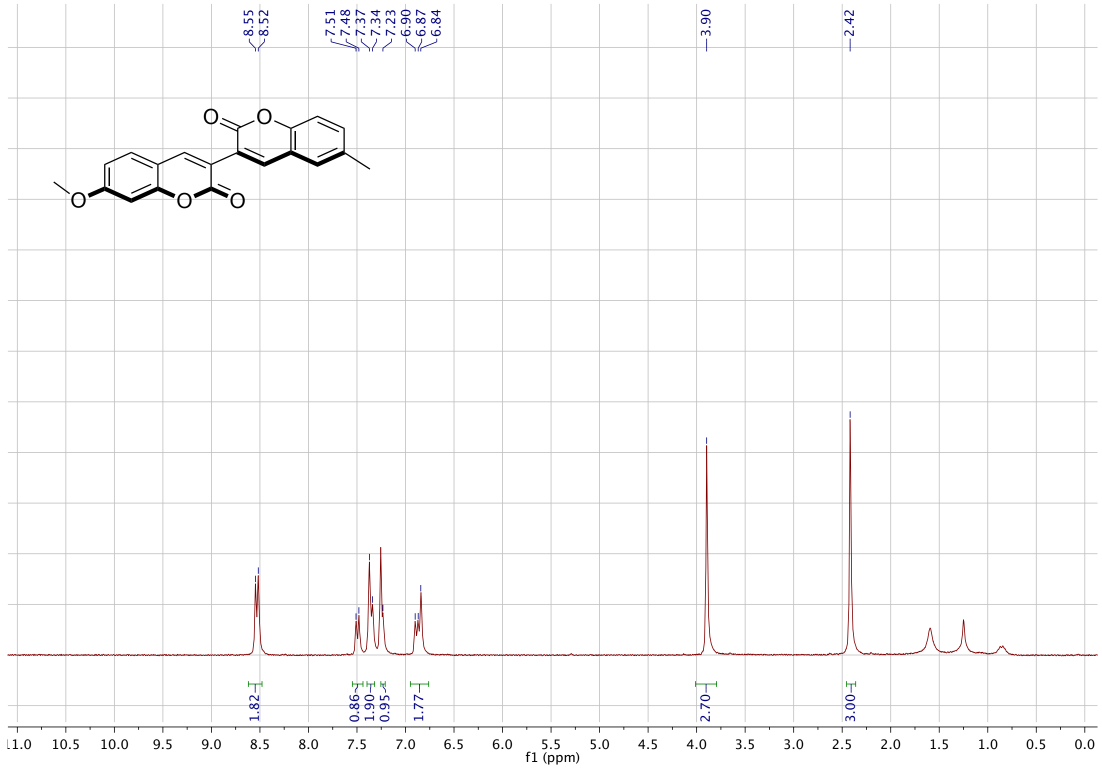

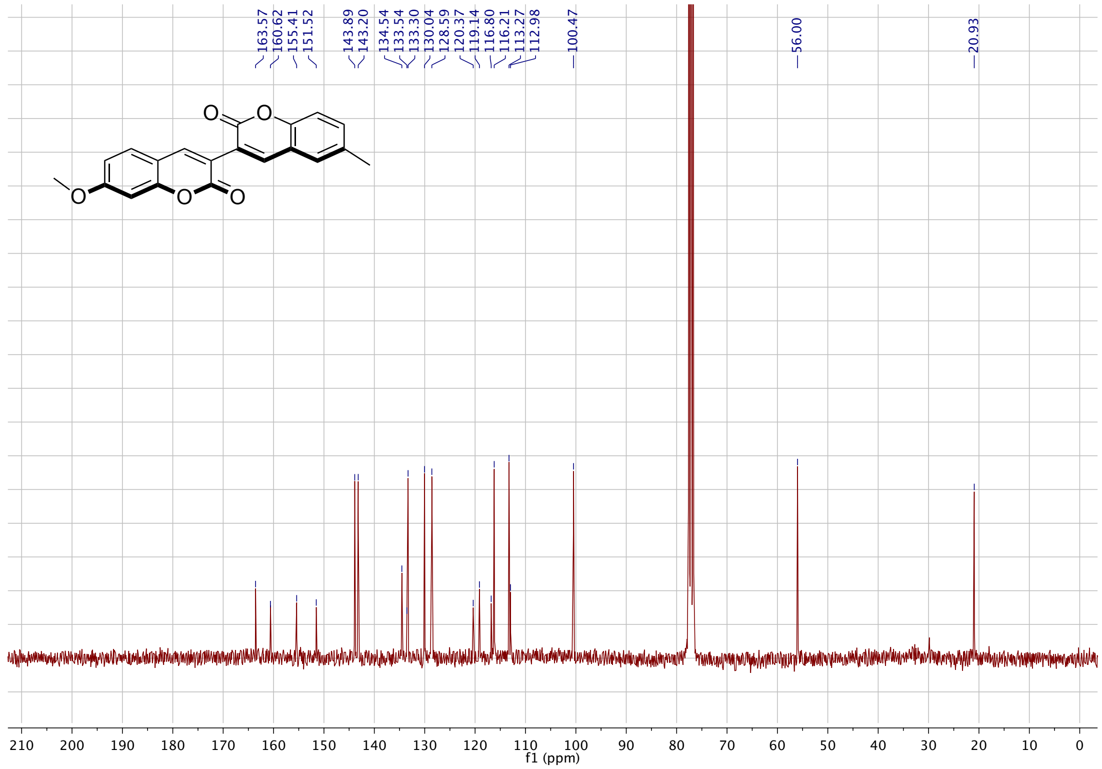

[1]**HNMR** (300 MHz, $CDCl_3$) and [13]**C CPMAS ssNMR** (125 MHz) for compound **9e**.

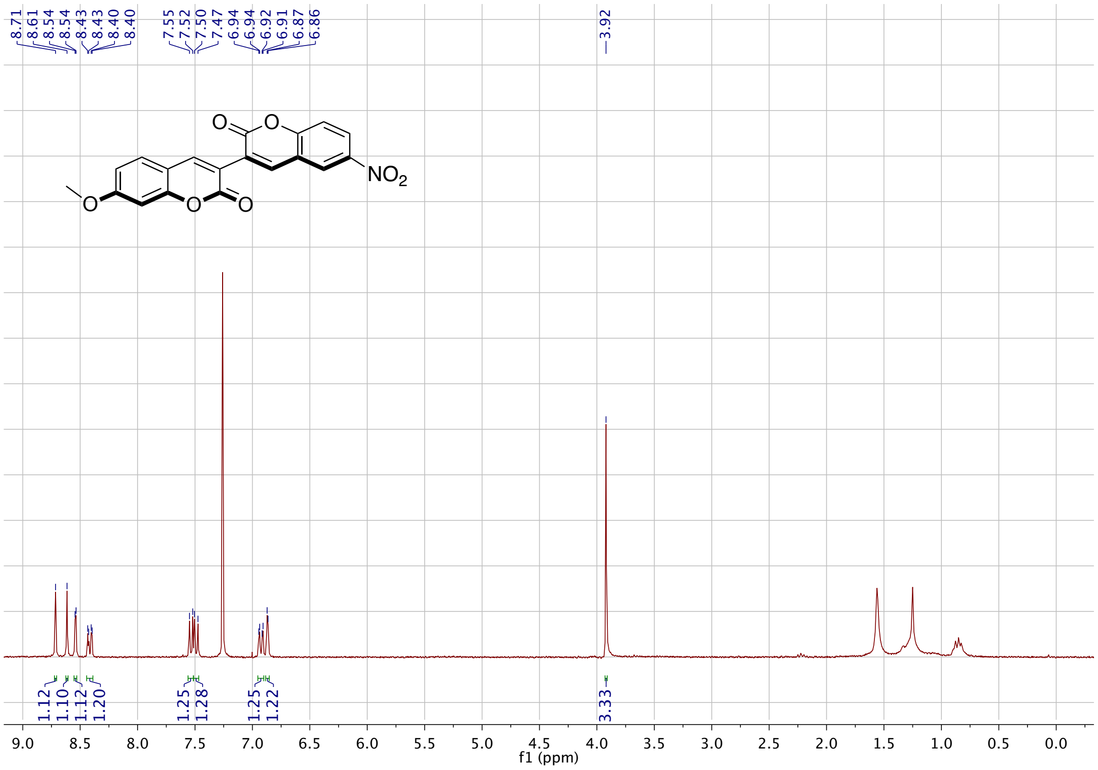

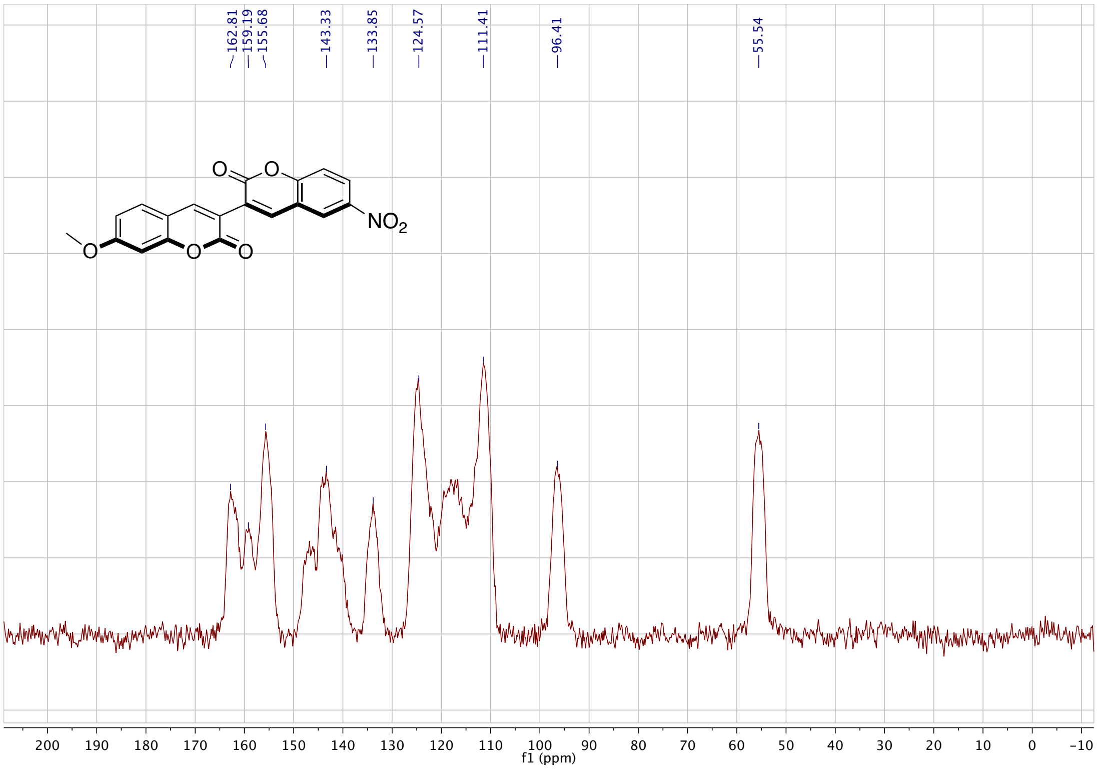

^{1}H NMR (300 MHz, $CDCl_3$) and **^{13}C CPMAS ssNMR** (125 MHz) for compound **9f.**

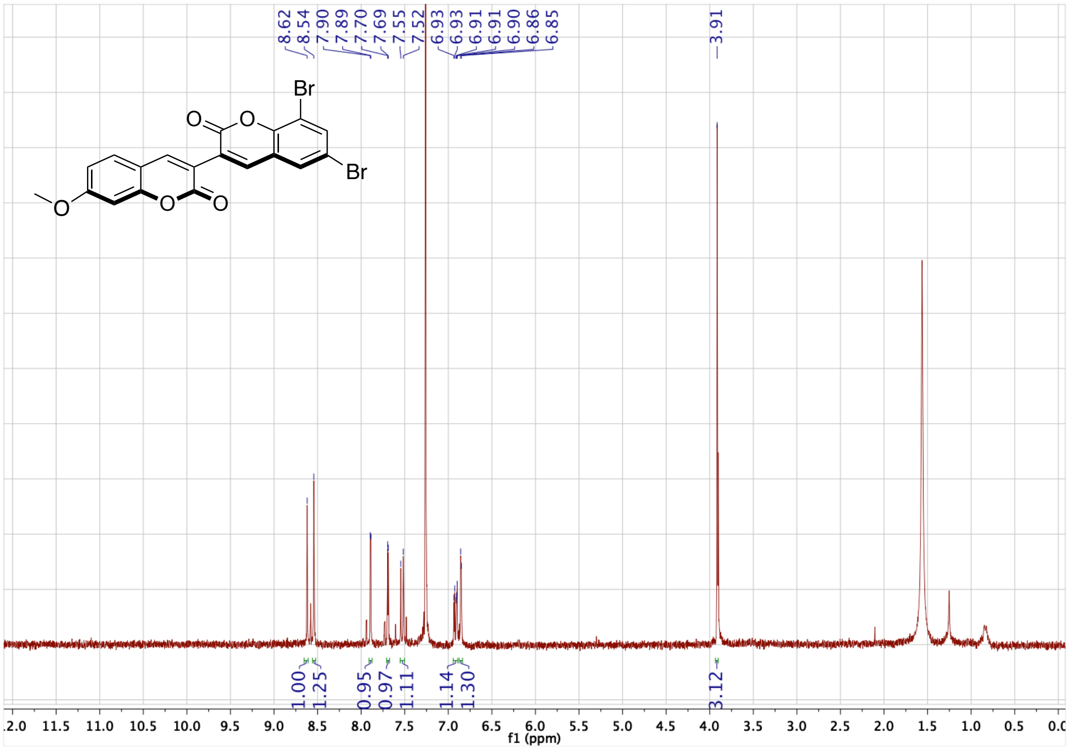

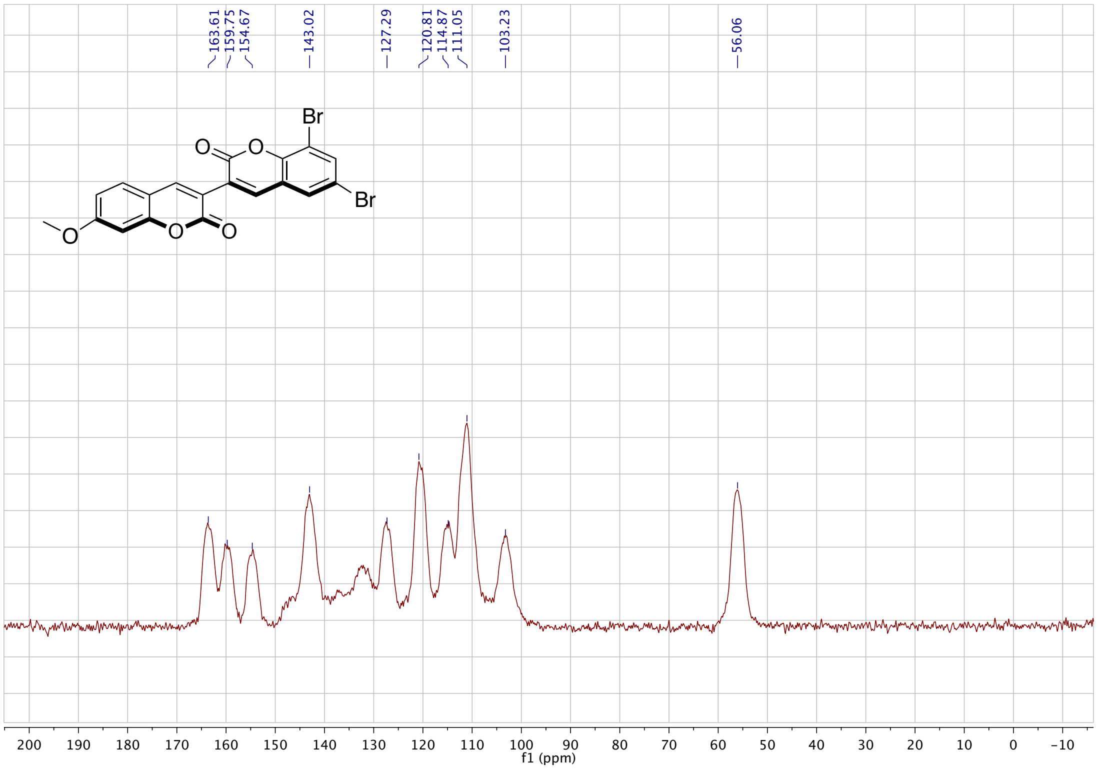

^{1}H NMR (300 MHz, $CDCl_3$) and **^{13}C CPMAS ssNMR** (125 MHz) for compound **9g.**

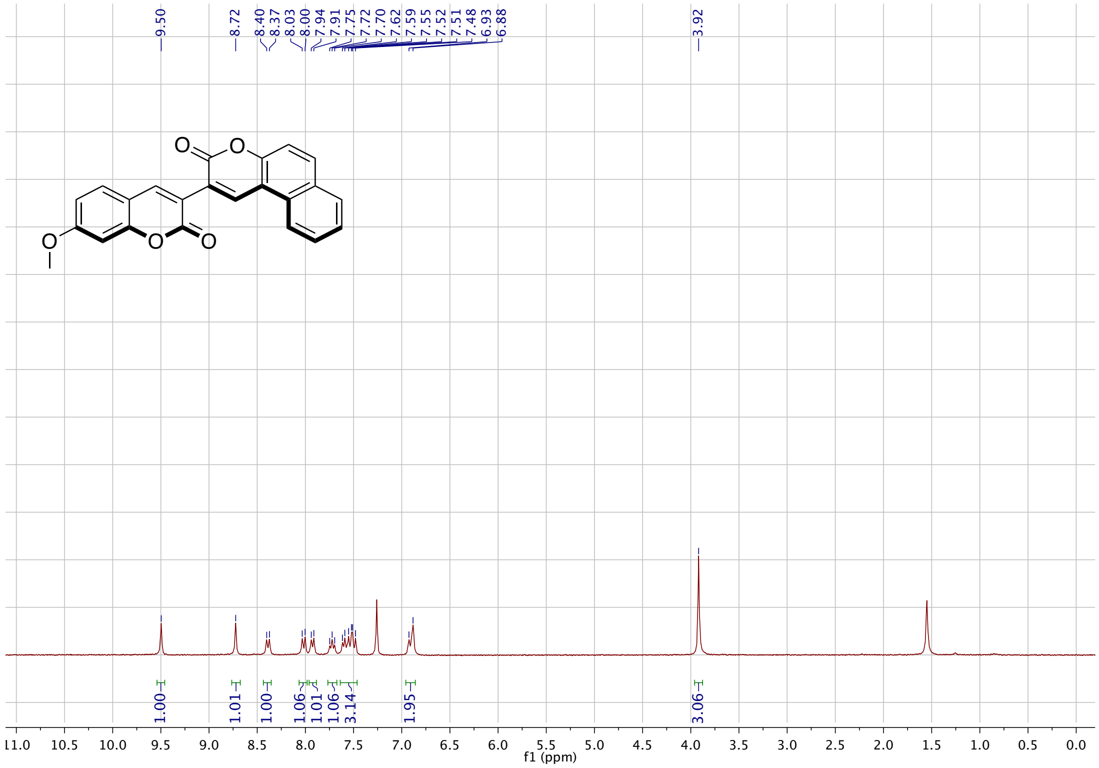

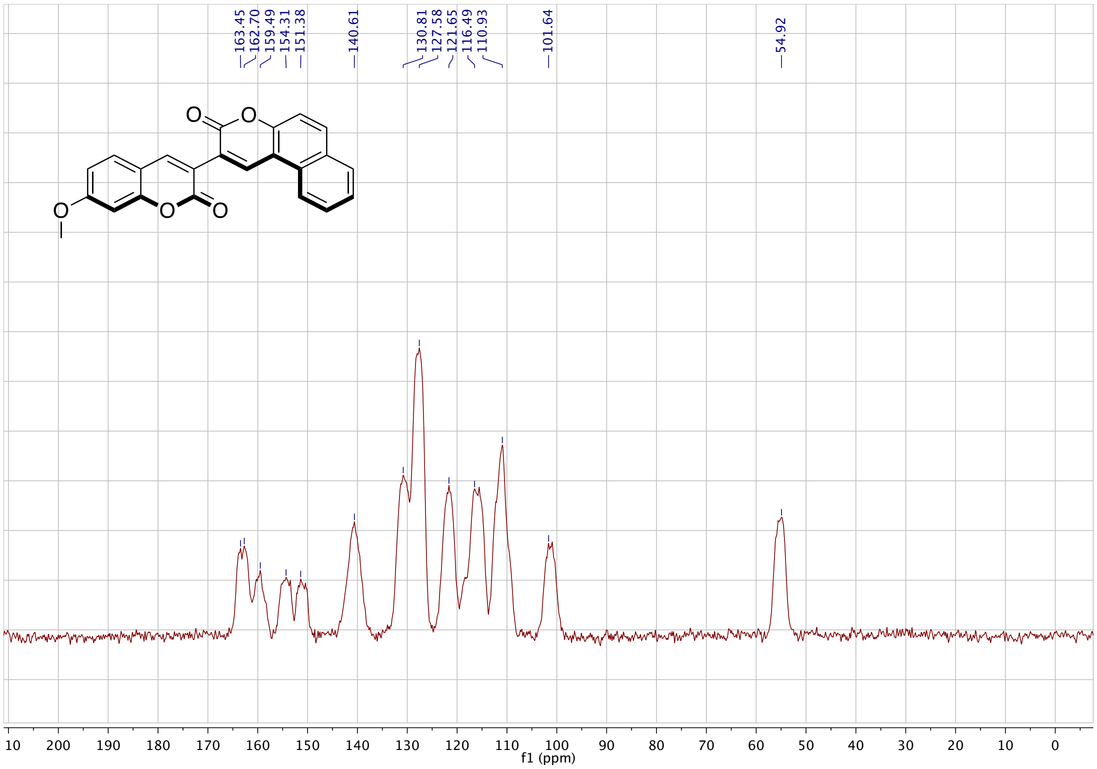

^{1}H NMR (300 MHz, $CDCl_3$) and **^{13}C NMR** (75 MHz, $CDCl_3$) for compound **9h.**

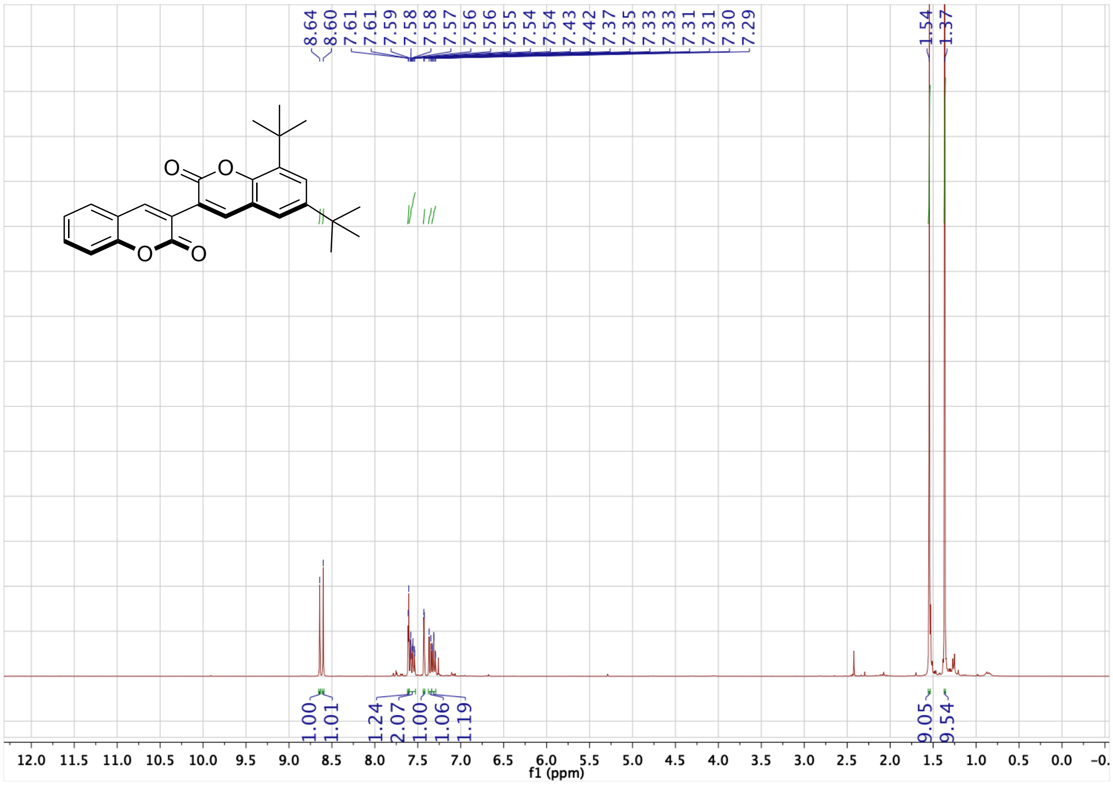

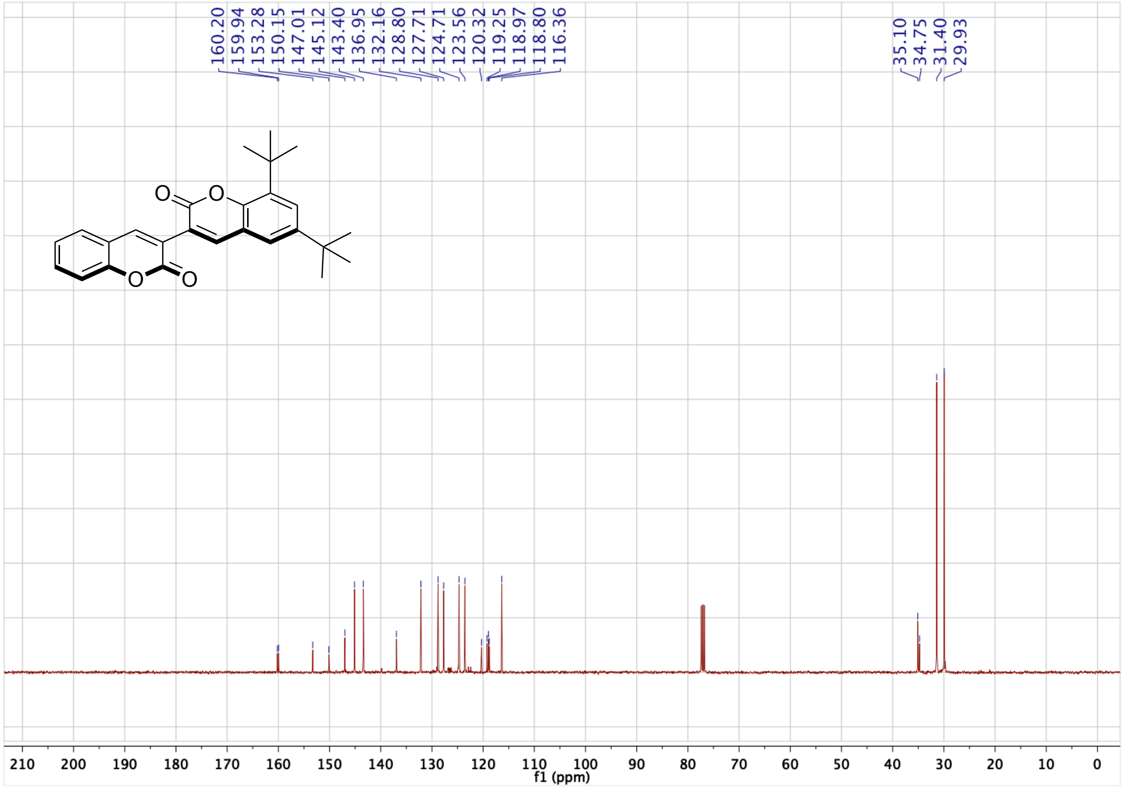

^{1}**H NMR** (300 MHz, $CDCl_3$) and ^{13}**C NMR** (75 MHz, $CDCl_3$) for compound **9i.**

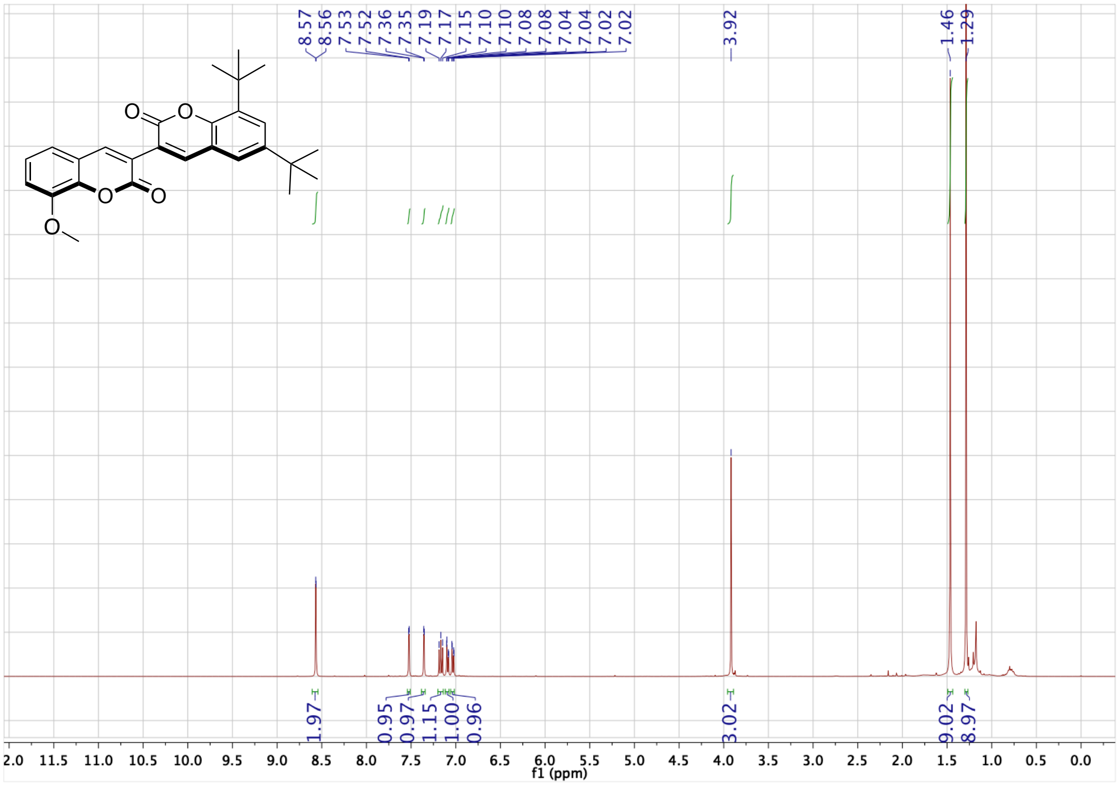

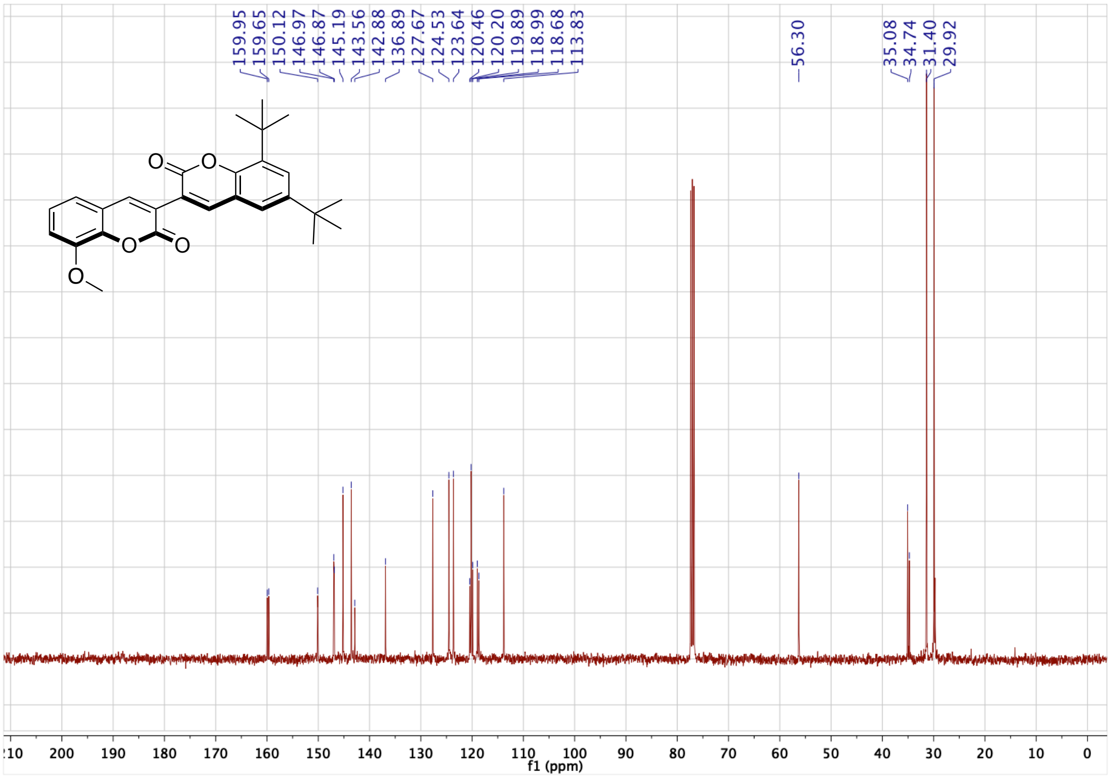

^{1}**H NMR** (300 MHz, $CDCl_3$) and ^{13}**C NMR** (75 MHz, $CDCl_3$) for compound **9j.**

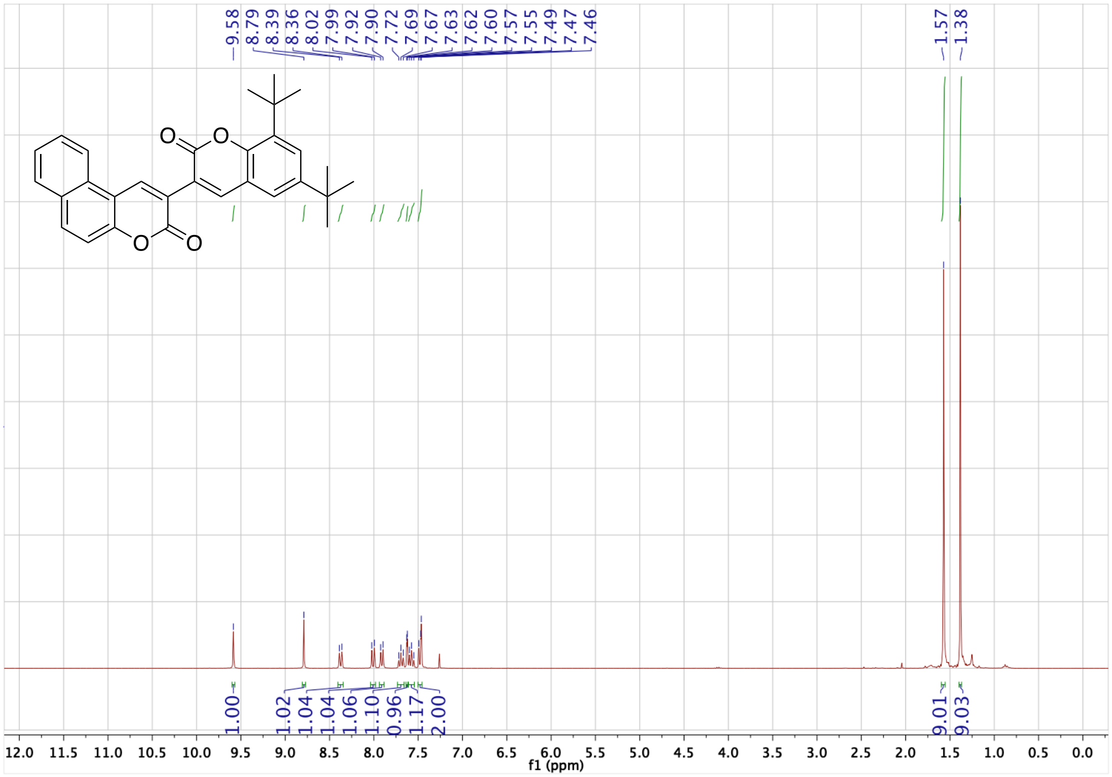

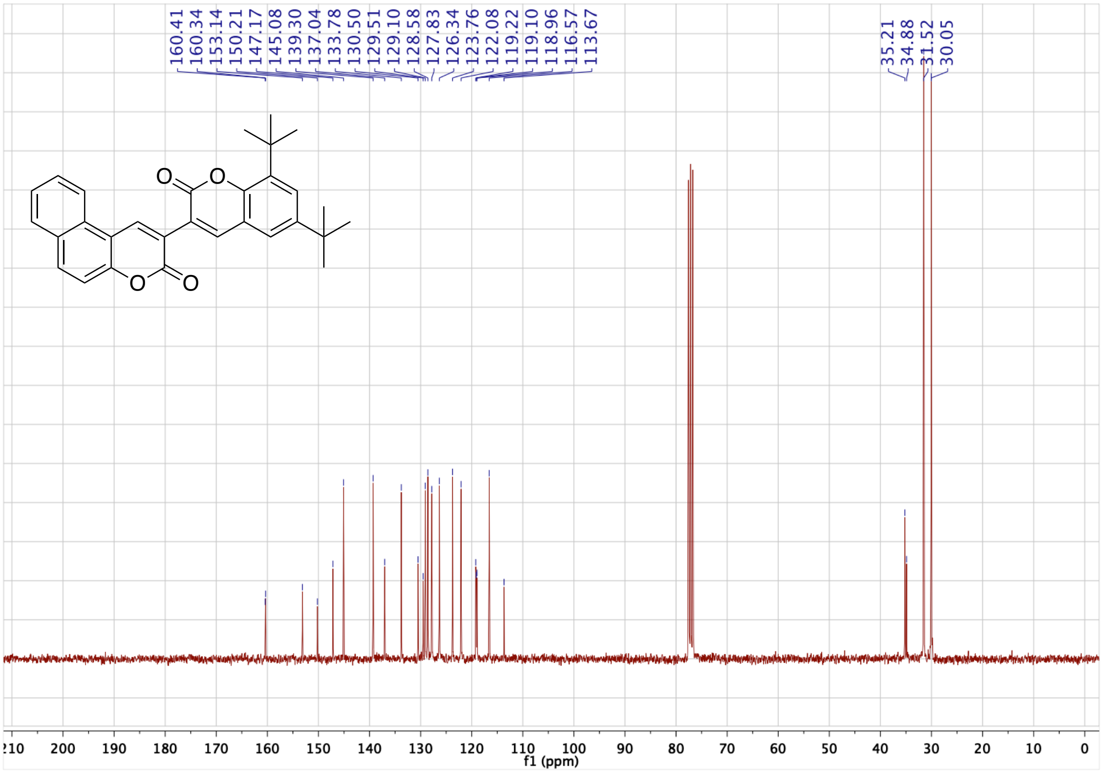

^{1}H NMR (300 MHz, $CDCl_3$) and **^{13}C NMR** (75 MHz, $CDCl_3$) for compound **9k.**

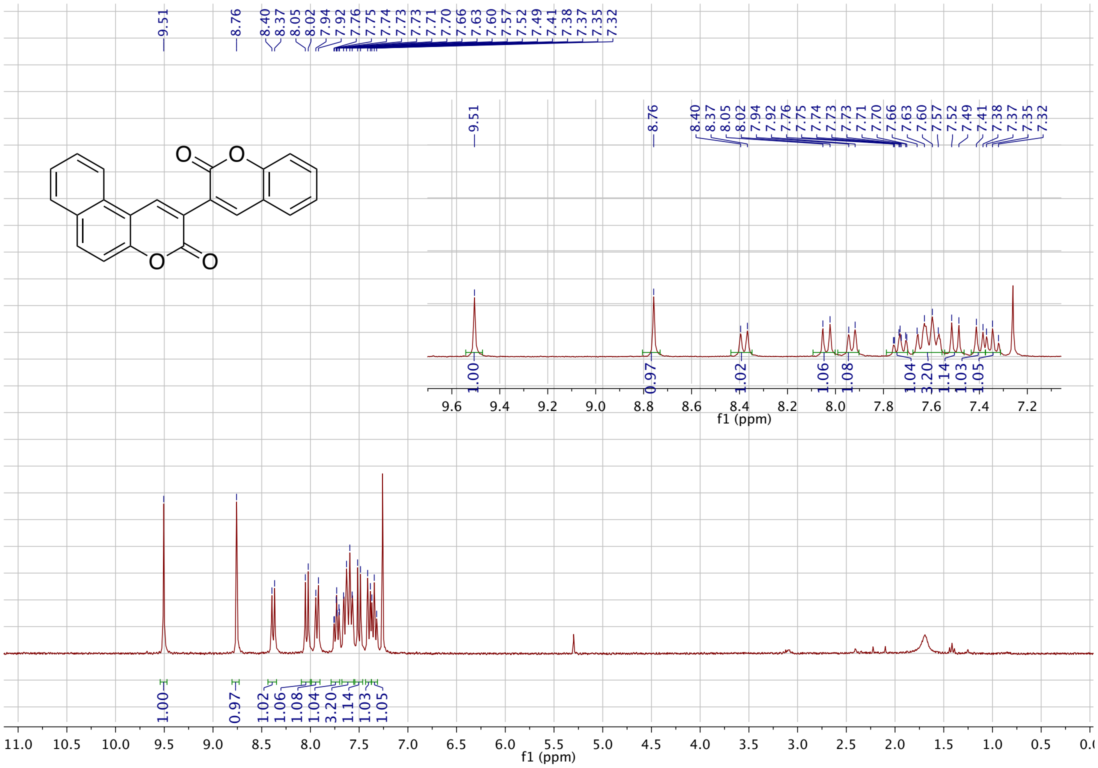

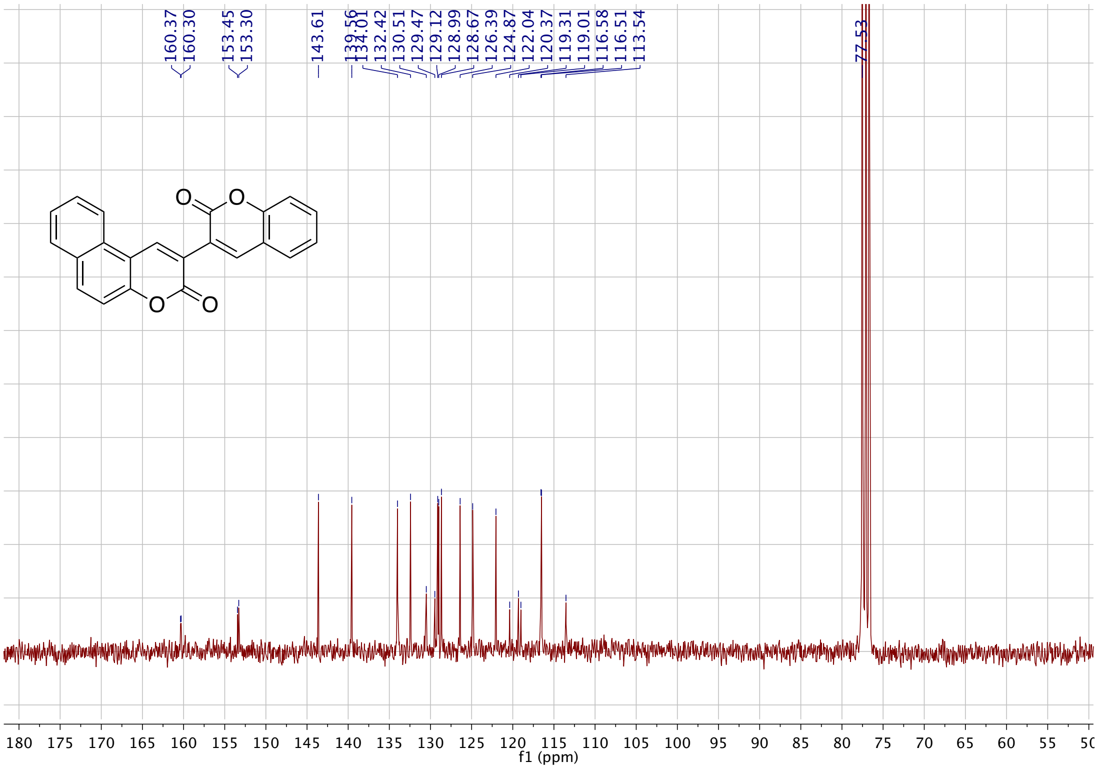

^{1}**H NMR** (700 MHz, $CDCl_3$) and ^{13}**C NMR** (175 MHz, $CDCl_3$) for compound **9l.**

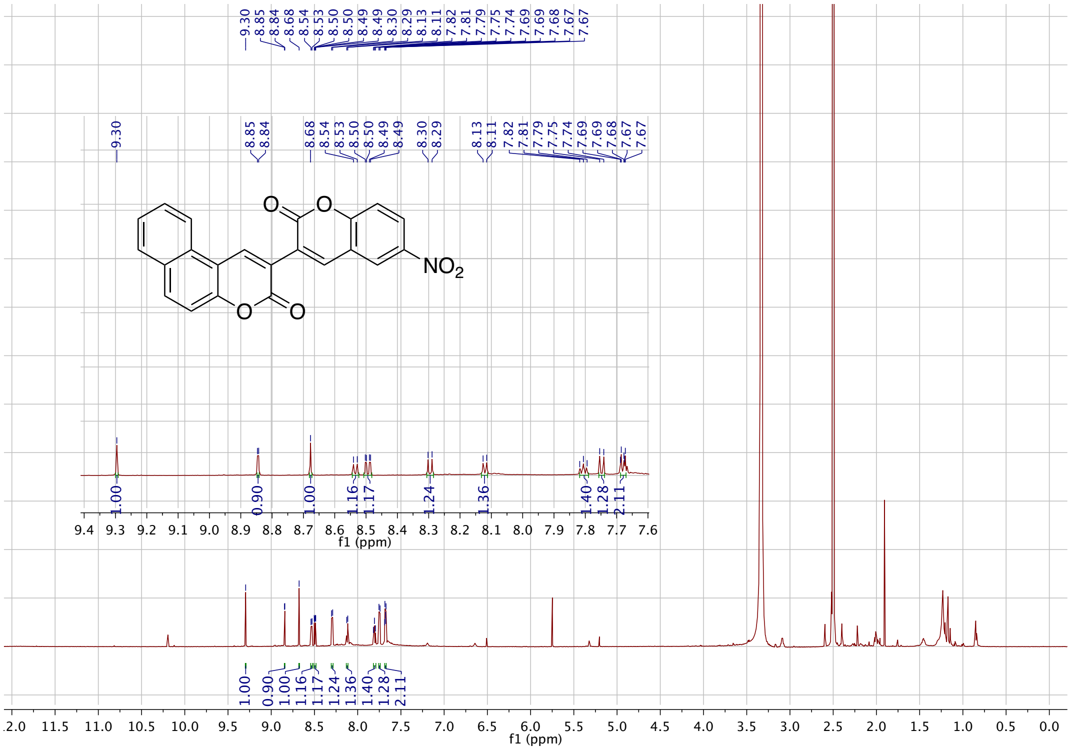

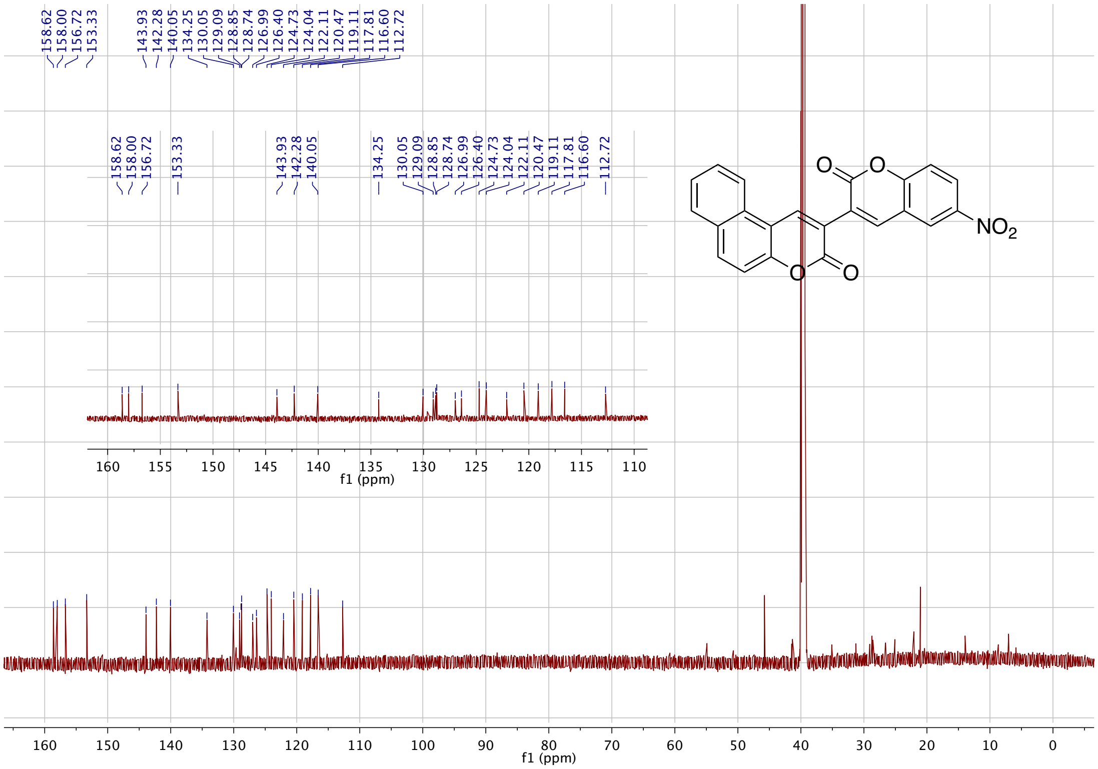

^{1}**H NMR** (300 MHz, $CDCl_3$) and ^{13}**C NMR** (75 MHz, $CDCl_3$) for compound **9m.**

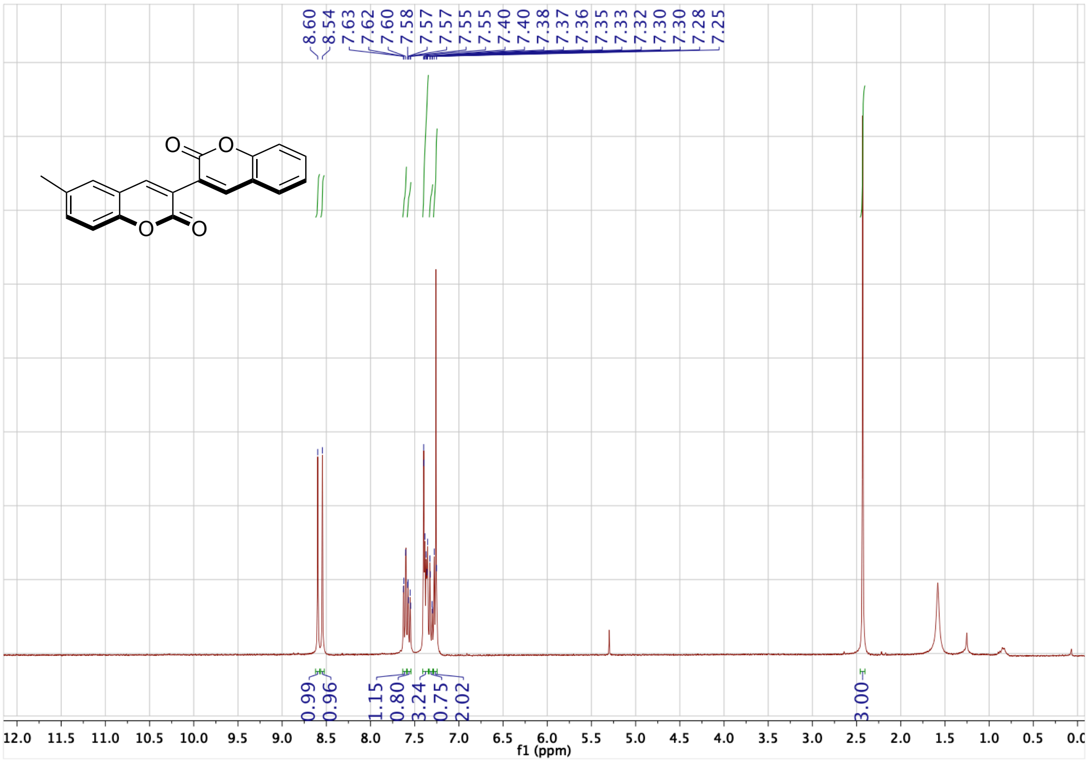

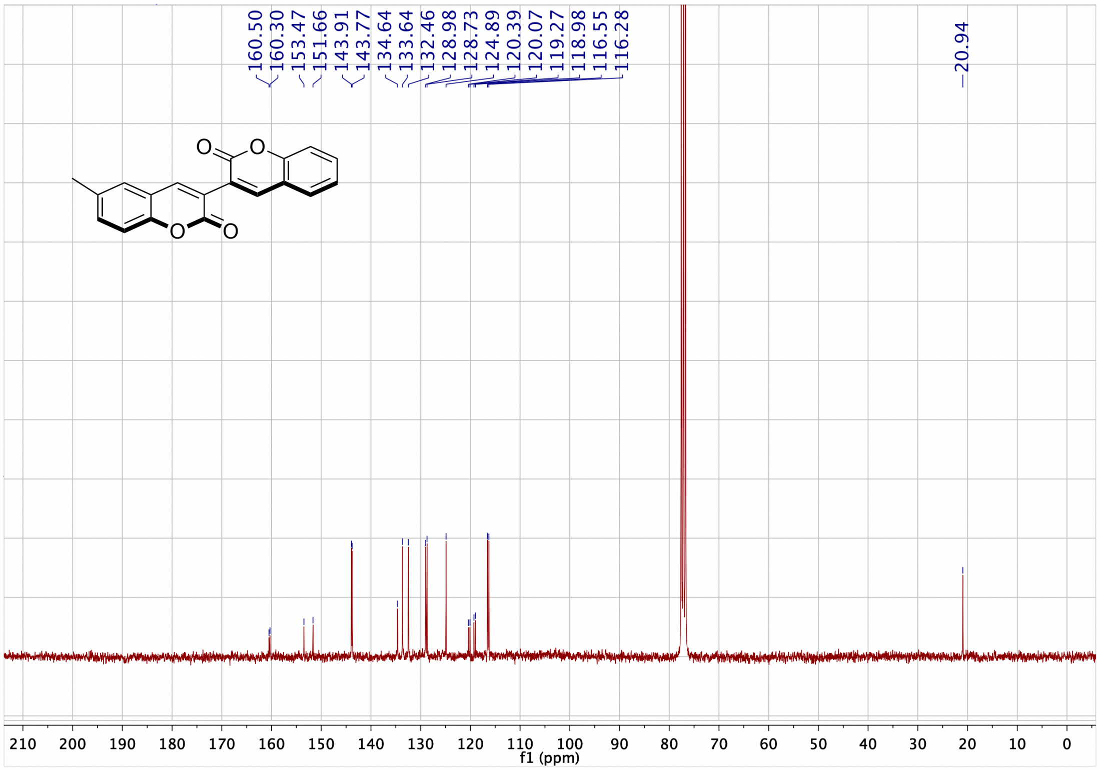

^{1}H NMR (300 MHz, $CDCl_3$) and **^{13}C NMR** (75 MHz, $CDCl_3$) for compound **9n.**

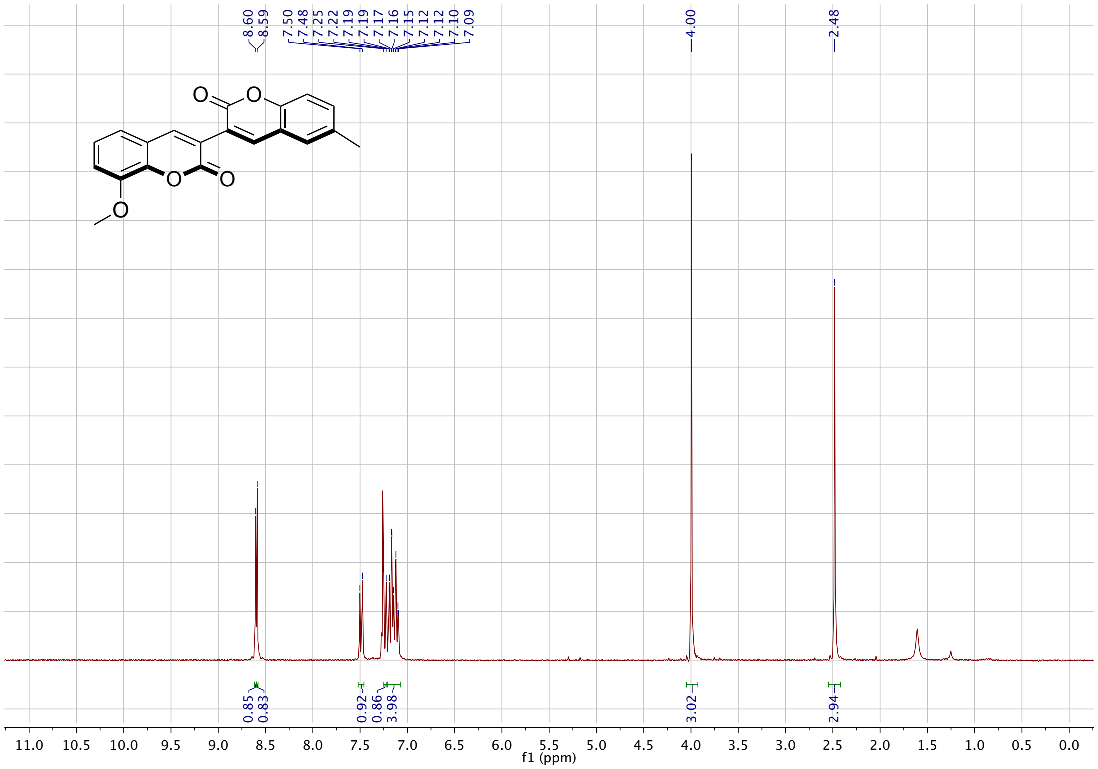

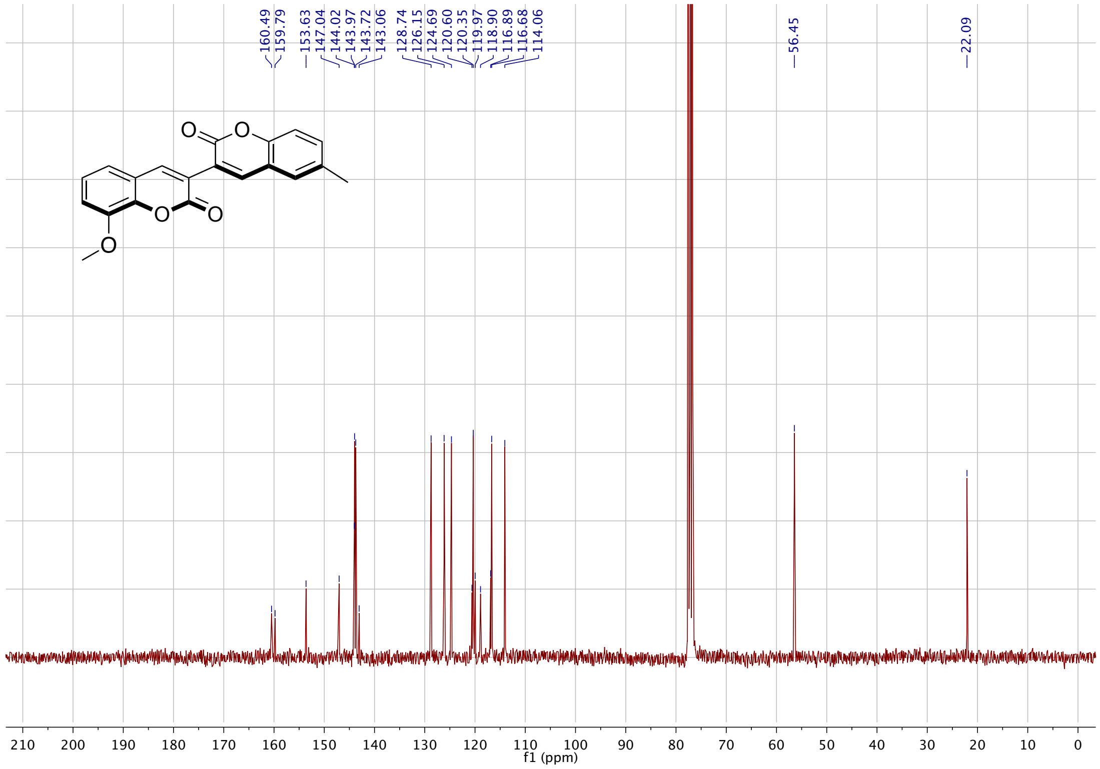

^{1}**H NMR** (300 MHz, DMSO) and ^{13}**C CPMAS ssNMR** (125 MHz) for compound **9o.**

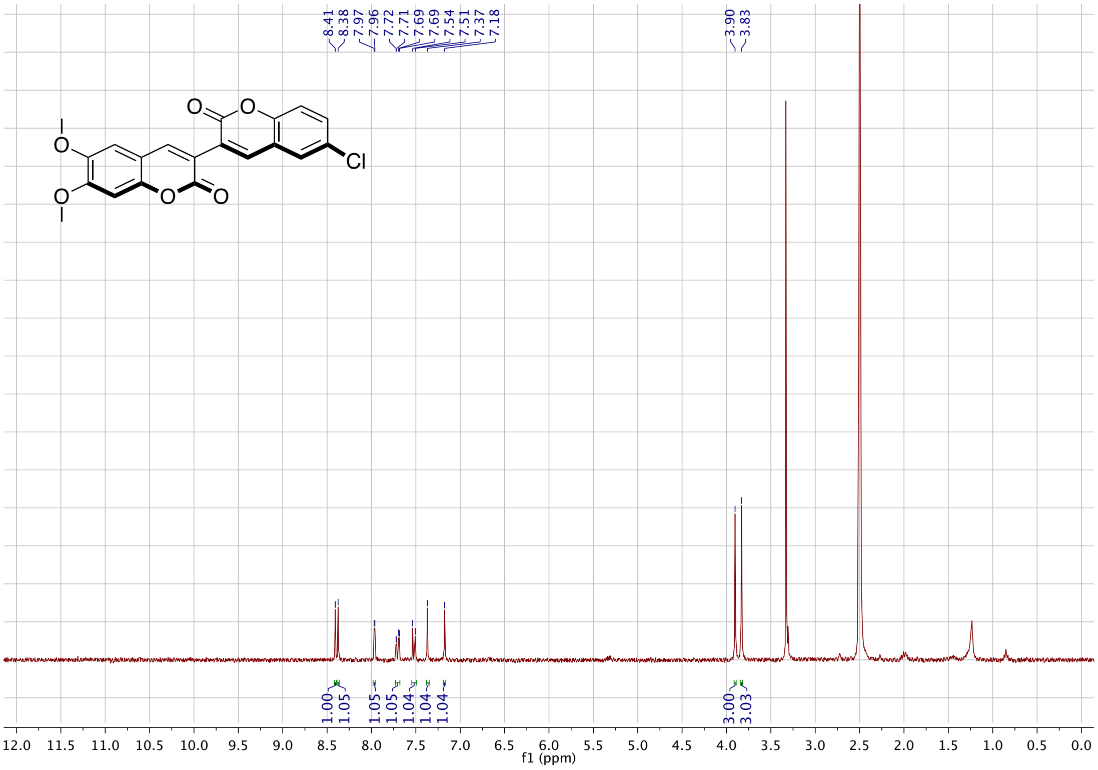

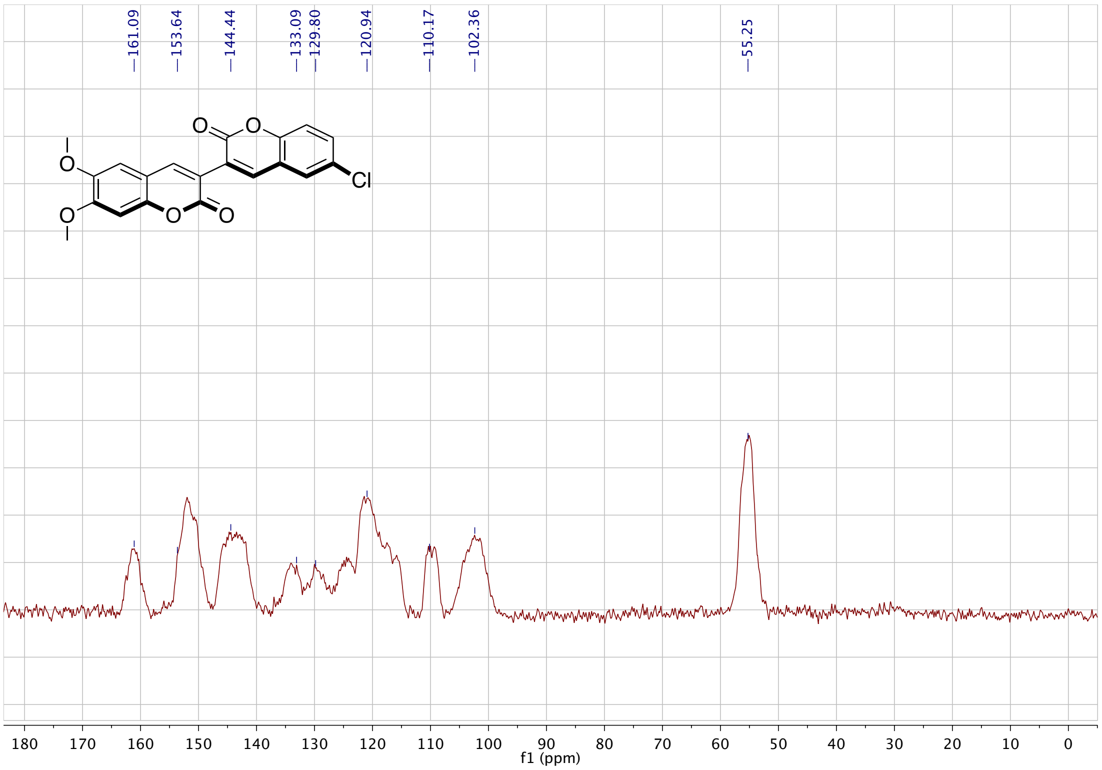

^{1}**H NMR (**700 MHz, $CDCl_3$**)** and ^{13}**C NMR (**175 MHz, $CDCl_3$) for compound **9p.**

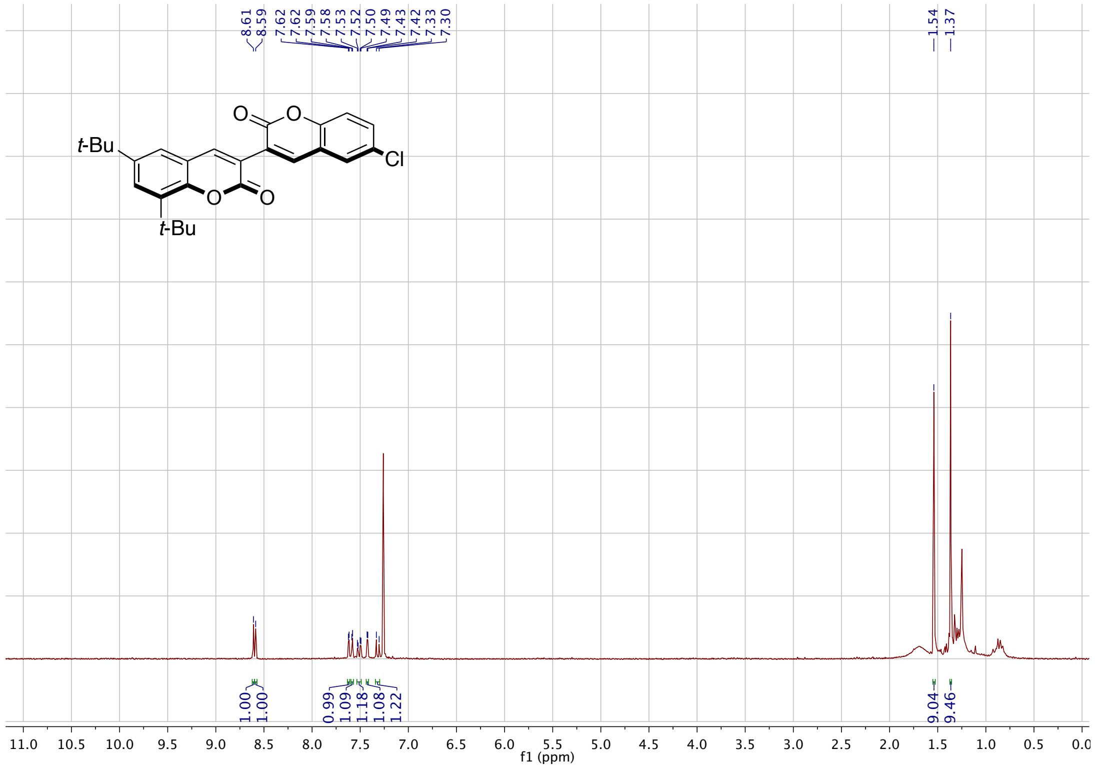

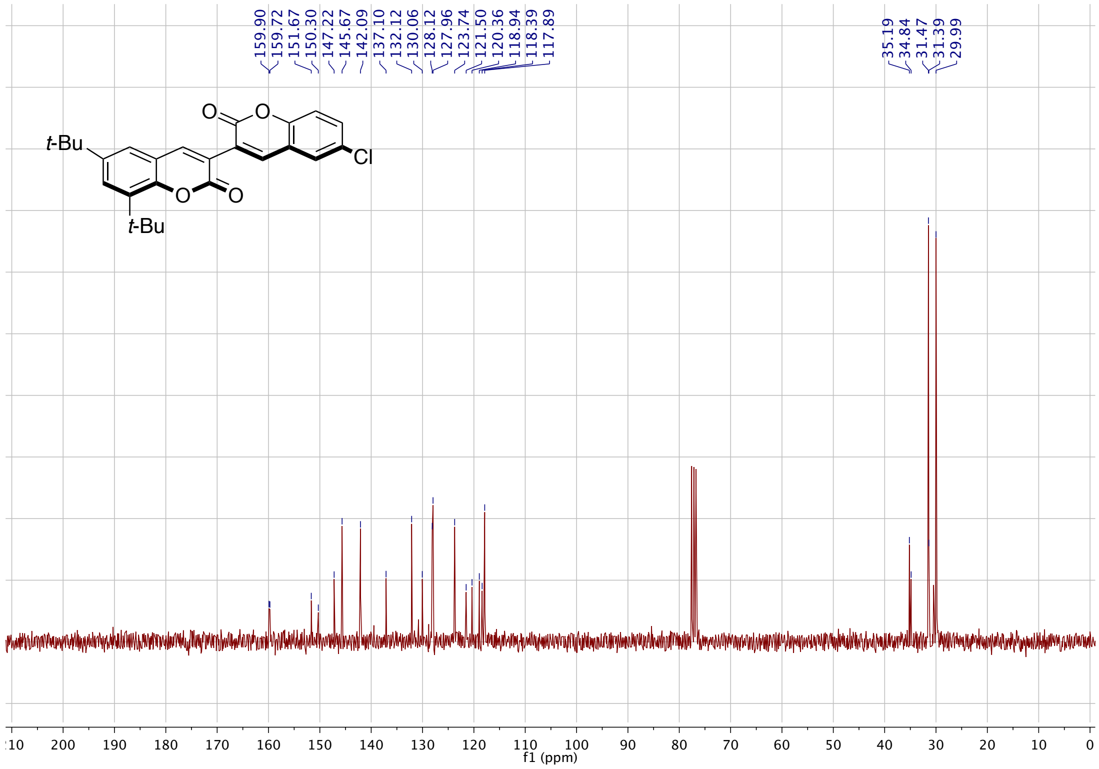

^{1}H NMR (300 MHz, $CDCl_3$) and **^{13}C NMR** (75 MHz, $CDCl_3$) for compound **9q.**

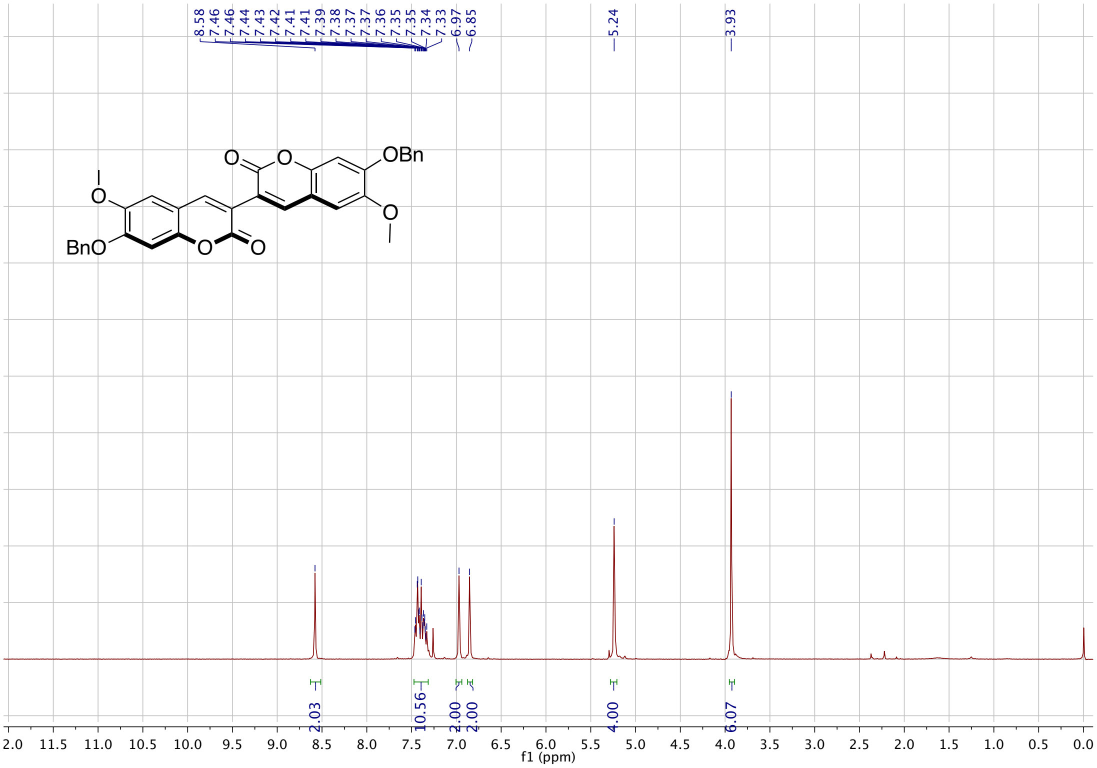

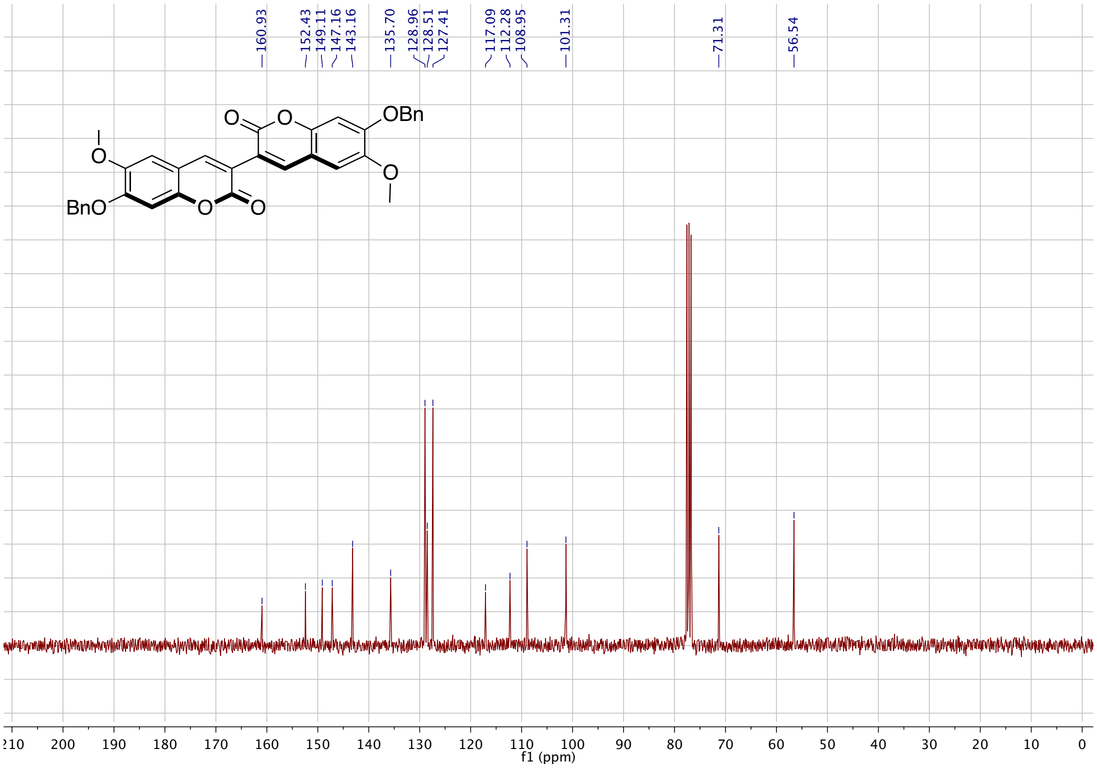

^{1}**H NMR** (300 MHz, $CDCl_3$) and ^{13}**C NMR** (75 MHz, $CDCl_3$) for compound **9r**.

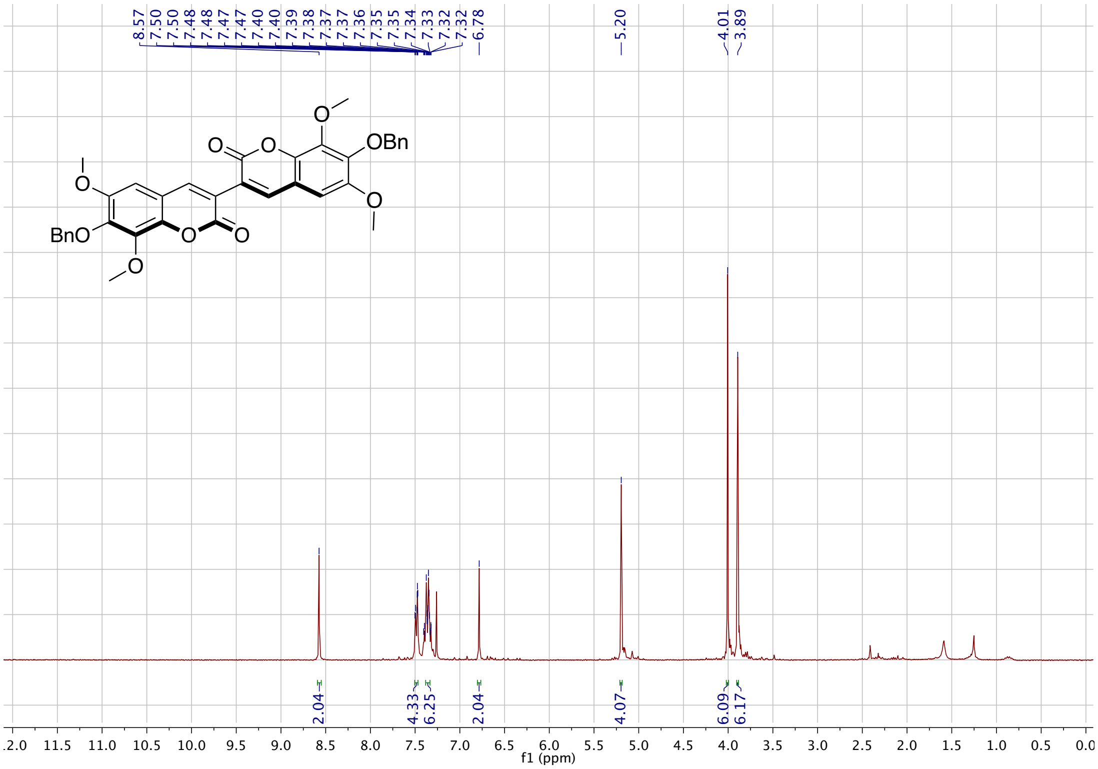

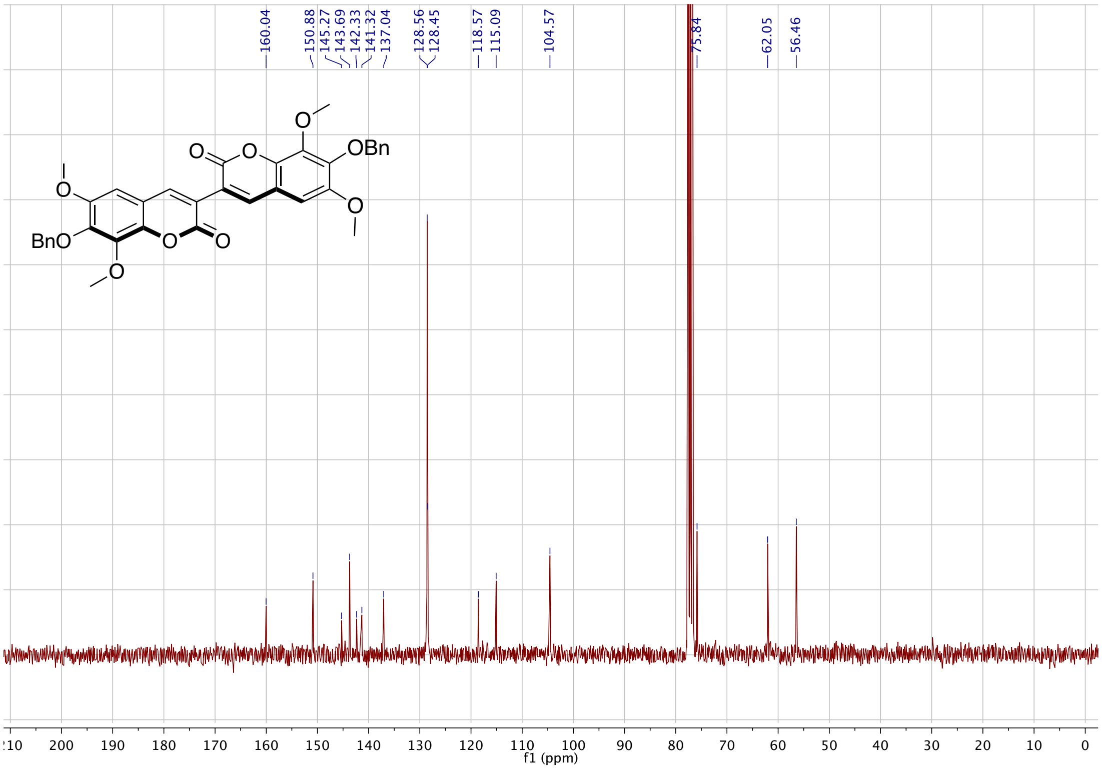

^{1}H NMR (300 MHz, $CDCl_3$) and **^{13}C NMR** (75 MHz, $CDCl_3$) for compound **9s.**

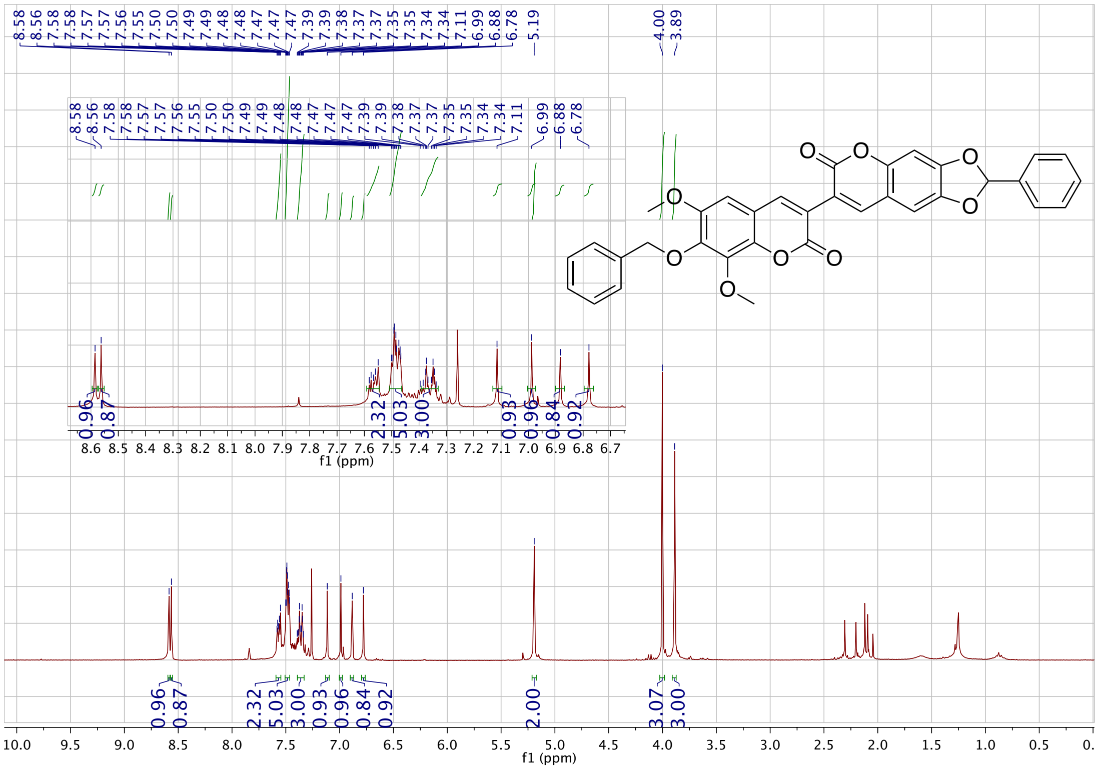

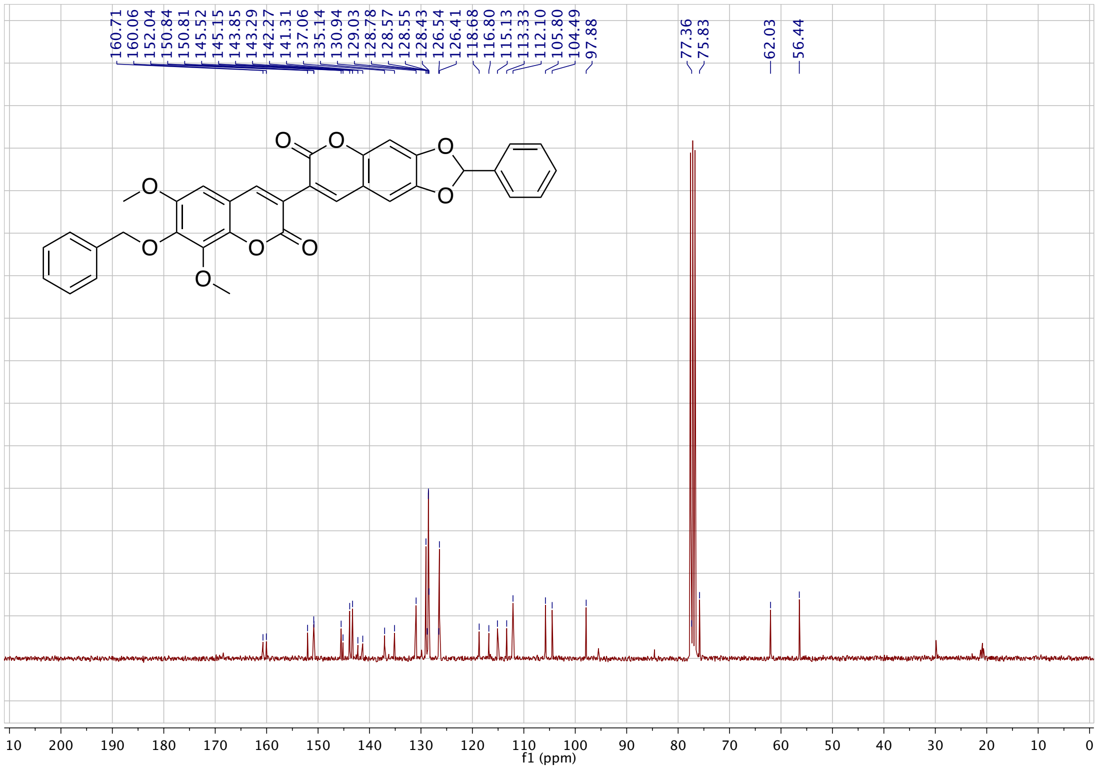

[1]**H NMR (**700 MHz, DMSO-d_6**)** and [13]**C NMR (**175 MHz, DMSO-d_6**)** for compound **Arteminorin C** (1)**.**

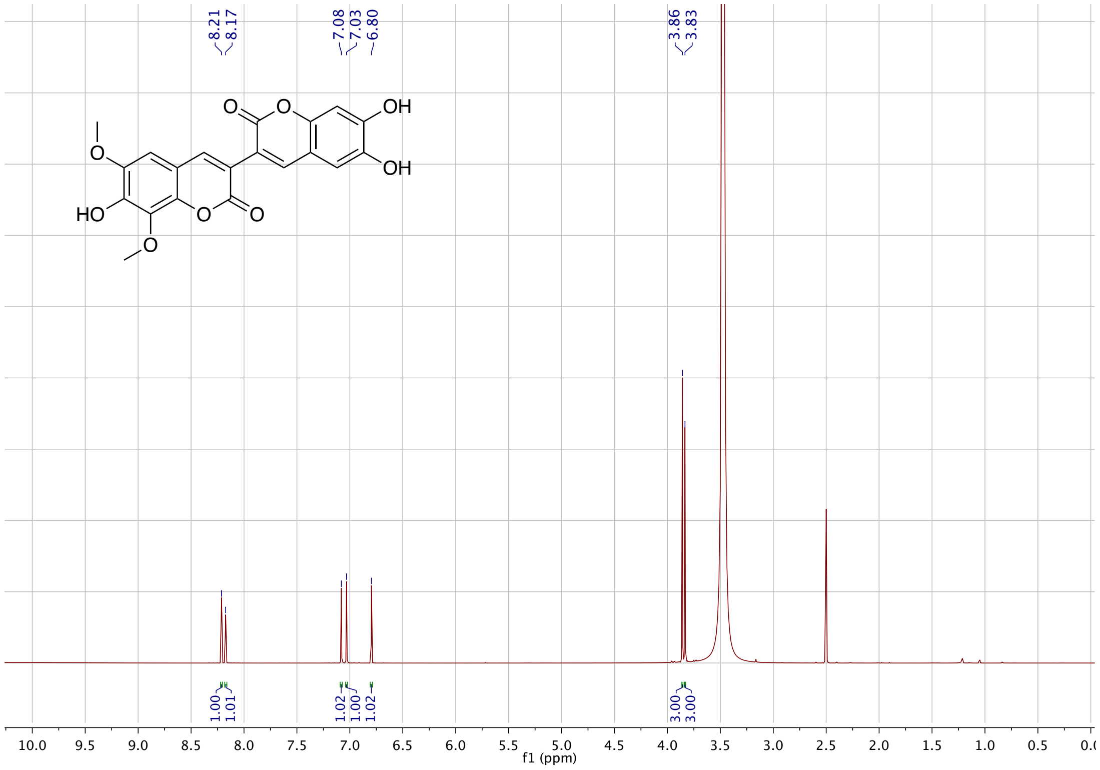

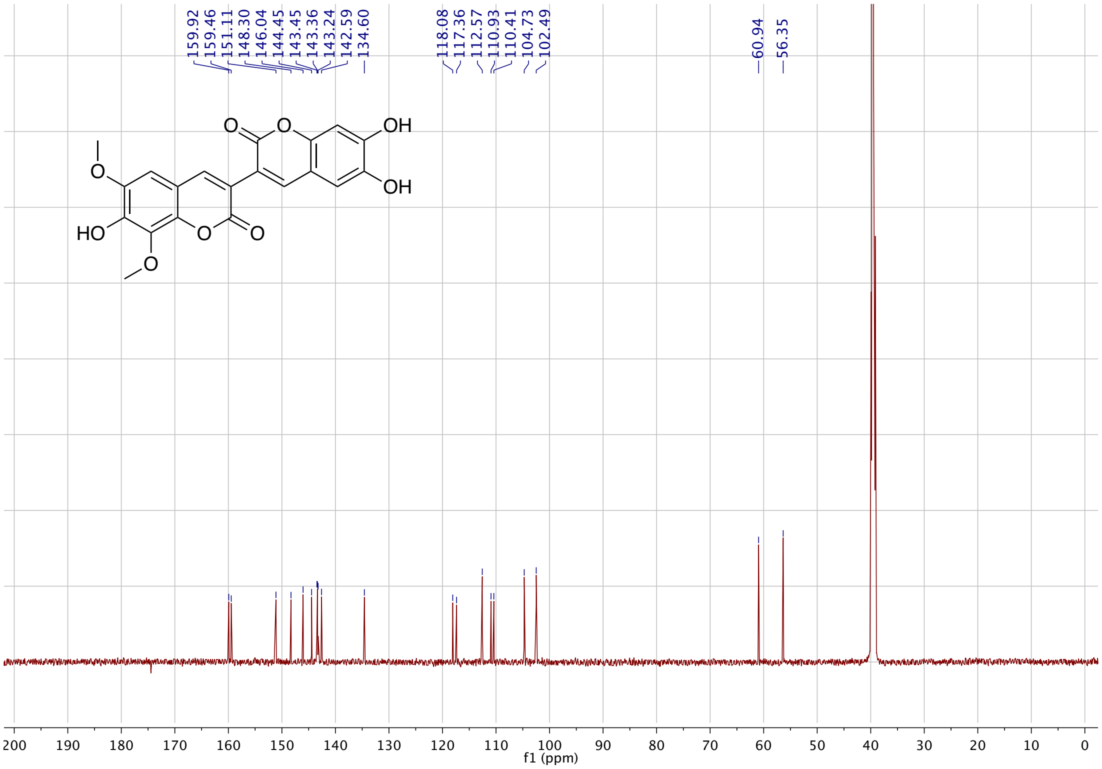

[1]**H NMR (**700 MHz, DMSO-d_6**)** and **^{13}C NMR (**175 MHz, DMSO-d_6**)** for compound **3,3´-biisofraxidin** (2)**.**

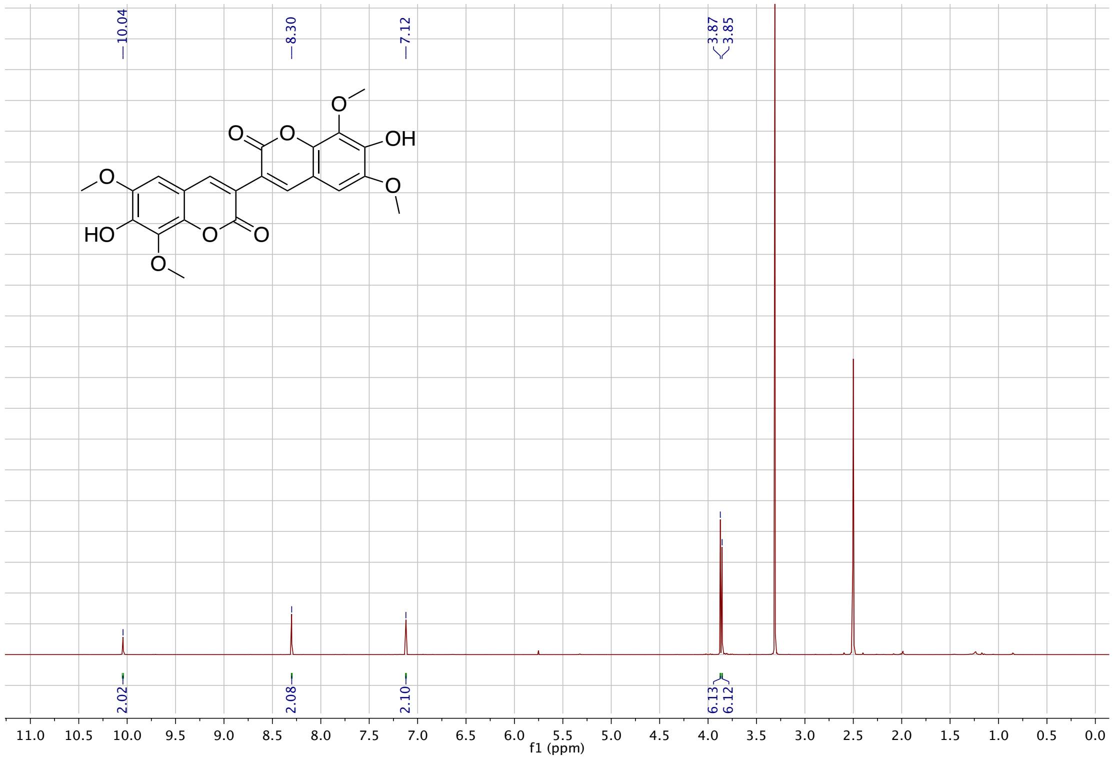

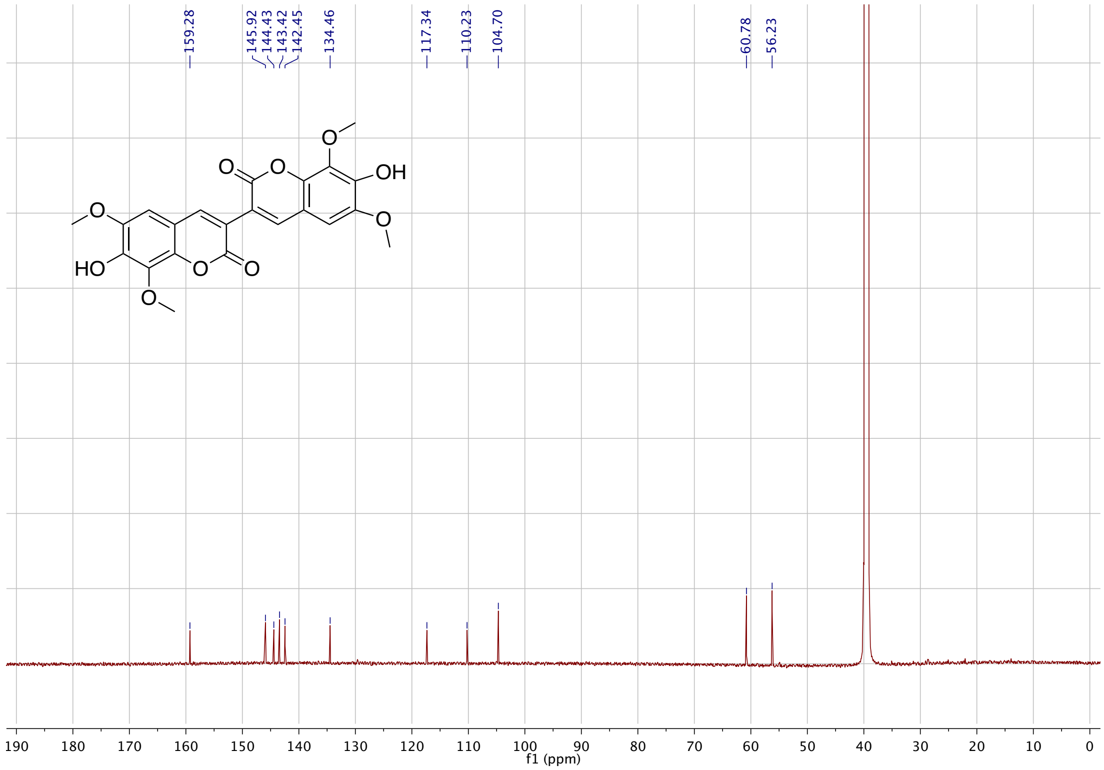

^{1}**H NMR (**700 MHz, DMSO-d_6**)** and ^{13}**C NMR (**175 MHz, DMSO-d_6**)** for compound **Biscopoletin** (3)**.**

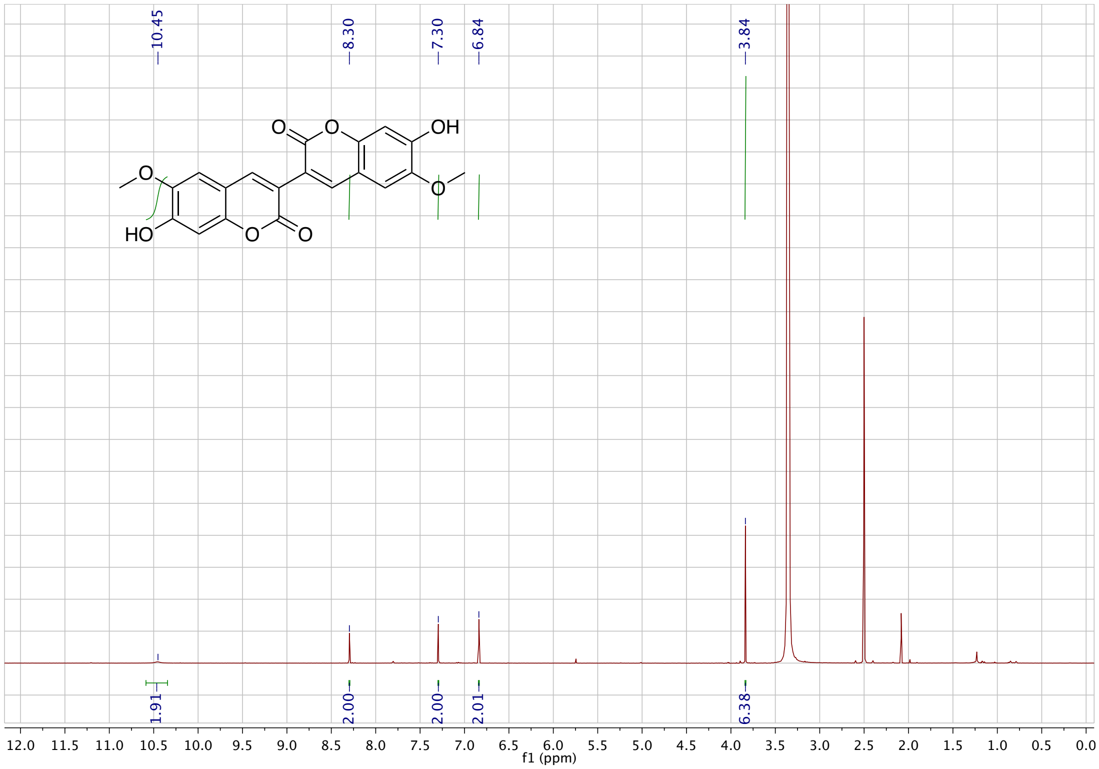

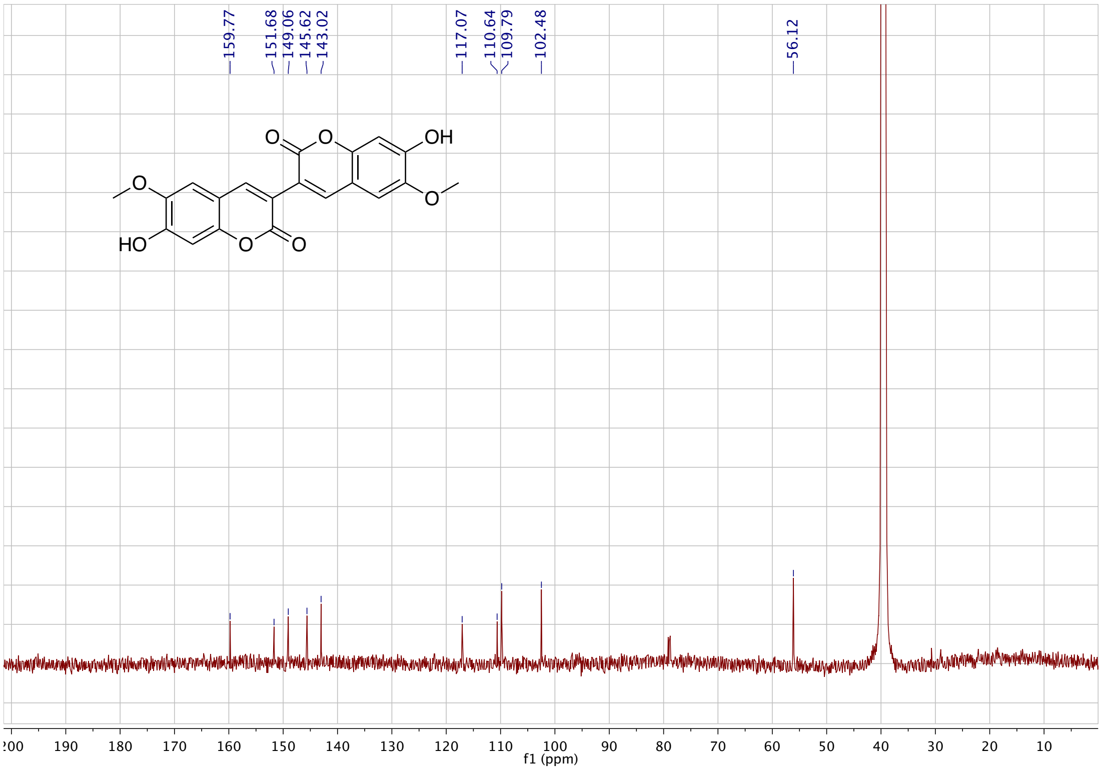

4. X-ray Crystallographic data

4.1 **X-Ray Crystallographic Data for Compound 9a**. Ellipsoid contour % probability: 50%. (CCDC 2416742).

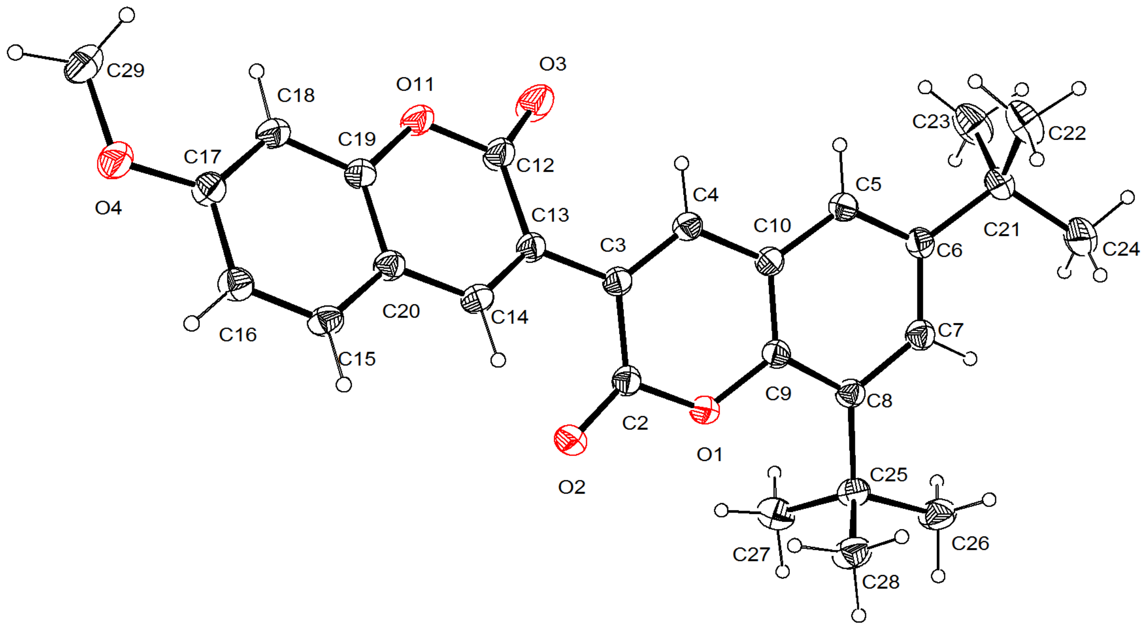

Table 7. Crystal data and structure refinement for **compound 9a**.

Empirical formula	$C_{27} H_{28} O_5$	
Formula weight	432.49	
Temperature	298(2) K	
Wavelength	1.54178 Å	
Crystal system	Orthorhombic	
Space group	Pbcn	
Unit cell dimensions	a = 13.5714(2) Å	α= 90°.
	b = 9.13300(10) Å	β= 90°.
	c = 37.1922(5) Å	γ = 90°.
Volume	4609.88(10) $Å^3$	
Z	8	
Density (calculated)	1.246 Mg/m^3	
Absorption coefficient	0.688 mm^{-1}	
F(000)	1840	
Crystal size	0.421 x 0.228 x 0.100 mm^3	
Theta range for data collection	4.032 to 68.315°.	
Index ranges	-16<=h<=13, -10<=k<=10, -37<=l<=44	

Reflections collected	21953
Independent reflections	4209 [R(int) = 0.0456]
Completeness to theta = 67.679°	99.8 %
Absorption correction	Semi-empirical from equivalents
Max. and min. transmission	0.7531 and 0.6771
Refinement method	Full-matrix least-squares on F^2
Data / restraints / parameters	4209 / 0 / 296
Goodness-of-fit on F^2	1.126
Final R indices [I>2sigma(I)]	R1 = 0.0645, wR2 = 0.1309
R indices (all data)	R1 = 0.0848, wR2 = 0.1434
Largest diff. peak and hole	0.198 and -0.176 e.$Å^{-3}$

5. Absorption and emission in different solvents of bicoumarins.

Table 8. Complete values of λ_{abs} and λ_{em} were obtained in different solvents for compounds **9a to 9p**. (Data of 9h was not obtained due the poor solubility of such compound). All data were collected at $1.5x10^{-5}$ M.

	Toluene			Dioxane			THF			DMF		
Compound	λ_{abs} (nm)	λ_{em} (nm)	Stokes Shift (nm)	λ_{abs} (nm)	λ_{em} (nm)	Stokes Shift (nm)	λ_{abs} (nm)	λ_{em} (nm)	Stokes Shift (nm)	$\Lambda_{A\ abs}$ (nm)	λ_{em} (nm)	Stokes Shift (nm)
**9a**	373	427	54	369	425	56	368	427	59	354	438	84
**9b**	370	426	56	366	423	57	365	426	61	352	443	91
**9c**	373	431	58	371	431	60	370	435	65	356	450	94
**9d**	371	427	56	367	424	57	367	425	58	354	438	84
**9e**	387	435	48	383	434	51	382	441	59	372	437	65
**9f**	377	435	58	372	432	60	372	438	66	361	427	66
**9g**	391	453	62	387	450	63	388	451	63	379	458	79
**9i**	362	417	55	358	415	57	342	417	75	334	439	105
**9j**	392	446	54	386	443	57	385	444	59	377	450	73
**9k**	388	445	57	385	441	56	385	444	59	373	448	75
**9l**	394	438	44	389	436	47	390	464	74	375	467	92
**9m**	361	413	52	357	410	53	355	410	55	340	415	75
**9n**	361	416	55	357	415	58	354	436	82	350	463	113
**9o**	366	455	89	362	461	99	361	472	111	347	508	161
**9p**	375	419	44	387	417	30	368	420	52	355	434	79

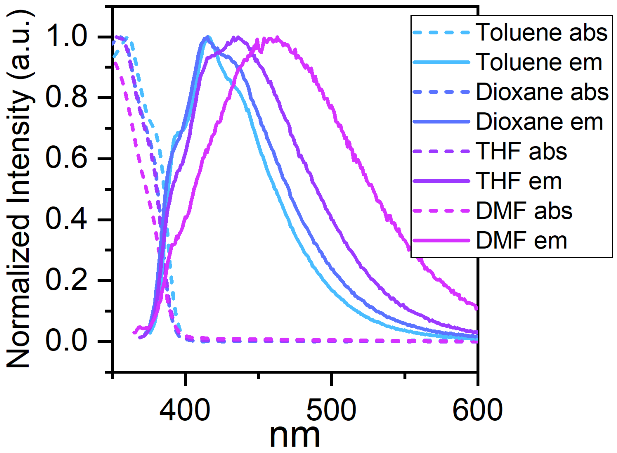

Figure 1. Normalized absorption and emission spectra of **9n** in different dissolvents.

6. References.

(1) He, Z.; Yan, J.; Song, Z.; Ye, F.; Liao, X.; Peng, S.; Ding, L. Chemical Constituents of the Aerial Parts of *Artemisia minor*. *J. Nat. Prod*. **2009**, *72*, 1198–1201.

(2) Wang, K.; Li, D.; Wu, B.; Cao, X. New Cytotoxic Dimeric and Trimeric Coumarins from *Chimonanthus salicifoliu*. *Phytochemistry Letters.* **2016**, *16,* 115–120.

(3) Wu, Q.; Zou, L.; Fu, D. Novel Sesquiterpene and Coumarin Constituents from the Whole Herbs of *Crossostephium chinense*. *J. Asian Nat. Prod. Res.* **2009,** *11*, 85–90.
